# Gut microbiome composition and predicted functions relate to growth and behavior in a Japanese preschool cohort

**DOI:** 10.1038/s41598-026-59018-4

**Published:** 2026-06-26

**Authors:** Shunsuke Ichikawa, Ayaka Shimura, Aoi Kikuchi, Rise Sanda, Kensaku Sasayama, Keiko Nonoue, Hiroko Tamura, Takahiro Kano, Yasuhito Shimada

**Affiliations:** 1 Faculty of Education, Mie University, 1577 Kurimamachiya-Cho Tsu, Mie, 514-8507 Japan; 2 Waseda Institute for Sport Science, Waseda University, 2-579-15 Mikajima Tokorozawa, Saitama, 359-1192 Japan; 3 Sanyo Gakuen University, 1-14-1 Hirai Naka-Ku Okayama, Okayama, 703-8501 Japan; 4 Zebrafish Research Center, Mie University, 1577 Kurimamachiya-Cho Tsu, Mie, 514-8507 Japan; 5 Department of Integrative Pharmacology, Graduate School of Medicine, Mie University, 2-174 Edobashi, Tsu, Mie, 514-8507 Japan; 6 Department of Life Science and Biotechnology, College of Bioscience and Chemistry, Kanazawa Institute of Technology, 3-1 Yatsukaho, Hakusan, Ishikawa, 924-0838 Japan; 7 Genome Biotechnology Laboratory, Kanazawa Institute of Technology, 3-1 Yatsukaho, Hakusan, Ishikawa, 924-0838 Japan

**Keywords:** Preschool children, Gut microbiota, Child behavior checklist, Behavioral problems, Microbial metabolic pathways, Microbiology, Neuroscience

## Abstract

Early childhood is a period of rapid brain maturation and gut microbiome assembly, when emerging behavioral difficulties can shape later mental health and learning trajectories. Microbiota–gut–brain communication has been implicated in neurodevelopment through microbial metabolites and immune signaling. However, most pediatric evidence comes from high-risk or clinically referred cohorts, and gut microbiome–related correlates of typical behavioral variation in community-based preschool children remain poorly defined. In a cross-sectional sample of typically developing Japanese preschool children, we observed exploratory nominal associations between behavioral variation within normative ranges and gut microbiome composition and predicted functions. Internalizing domains showed candidate links with taxa and predicted pathways related to inflammatory potential and nucleotide biosynthesis, whereas somatic complaints and withdrawn behavior showed nominal associations with lower predicted respiratory and fermentative activity. Sleep-related difficulties showed multiple representative nominal pathway-level associations, including pathways related to methyl-donor and heme biosynthesis, while externalizing domains showed candidate links with predicted cell-envelope and carbohydrate-remodeling pathways. In contrast, age, height, and weight tracked expected maturation-related microbiome features, indicating that behavioral associations were not simple proxies of growth. Together, these findings provide an exploratory profile of microbiome–behavior correlations in a low-risk Japanese preschool cohort and highlight pathway-level candidates that may interface with neurodevelopment.

**Supplementary Information:**

The online version contains supplementary material available at https://doi.org/10.1038/s41598-026-59018-4.

## Introduction

Preschool mental health problems are common and impairing rather than transient. Large epidemiological cohorts and a meta-analysis suggest that about 15–20% of children aged 1–7 years meet criteria for at least one mental disorder^1^. Symptoms cluster into internalizing, externalizing, and sleep or regulation problems, with sufficient consistency that preschool adaptations of standard diagnostic nosologies have been proposed^2^. Longitudinal studies show that distinct internalizing, externalizing, and co-occurring profiles are detectable by 18 months to 3 years; persistent co-occurring problems predict the greatest difficulties at school entry; and early symptom clusters, such as sleep problems at 18 months, predict increased internalizing and externalizing problems at 5 years^3,4^. Standardized parent-report instruments are therefore central in research and practice. The Child Behavior Checklist for Ages 1½–5 (CBCL 1½–5) is widely used, provides empirically derived syndrome scales spanning internalizing, externalizing, and sleep-related problems, and shows robust factor structure and construct validity in population-based and clinical samples, including preschool children with autism spectrum disorder^3,5^. Its use in longitudinal cohorts that track trajectories from toddlerhood into later childhood enables internationally comparable phenotyping in the present study^3,5^.

Among candidate biological markers, the gut microbiota and the microbiota–gut–brain axis have gained prominence. Intestinal microbes shape host metabolism, immune activation, and neuroendocrine signaling, and communicate with the brain via short-chain fatty acids, bile acids, tryptophan metabolites, cytokines, and vagal and enteric pathways^6^. In animal models, germ-free rearing, early antibiotic exposure, and colonization with defined microbial communities alter microglial maturation, myelination, hippocampal and prefrontal structure, stress responsivity, and anxiety- and depression-like behaviors, and several effects can be partly normalized by restoring a complex microbiota or administering psychobiotic strains^7,8^. Fecal microbiota transplantation provides further causal evidence: microbiota from individuals with major depression, autism spectrum disorder, or attention-deficit/hyperactivity disorder can induce changes in brain structure, functional connectivity, and emotional or hyperactivity-like phenotypes in recipient mice^8,9^. In adults, population-based metagenomics links neuroactive taxa and pathway-level features, including butyrate-producing genera such as *Faecalibacterium* and *Coprococcus* and microbial modules for γ-aminobutyric acid and dopamine metabolism, with depressive symptoms and health-related quality of life, although effects are modest and heterogeneous across cohorts^10–12^. Together, these findings motivate testing microbiota–behavior links during early childhood, when both the microbiome and neurocognitive systems mature rapidly.

Human cohort studies now extend this evidence into development from pregnancy to middle childhood, but results vary by age, population, and outcome measures. In the Barwon Infant Study, higher maternal third-trimester gut microbiota α-diversity and greater abundances of butyrate-producing Lachnospiraceae and Ruminococcaceae predicted fewer internalizing problems at 2 years of age on the CBCL^13^. In the same setting and other birth cohorts, infant gut microbiota composition and diversity during late infancy forecast CBCL internalizing problems and broader behavioral outcomes at 2–3 years, with taxa such as *Prevotella* and *Bacteroides* often implicated^14^. Longer-term community studies report that microbiota trajectories across the first 3 years relate to problem behavior and executive functions, and that patterns of diversity and composition across the first 14 years associate with internalizing, externalizing, and social anxiety symptoms around puberty^15,16^. In typically developing preschoolers, cross-sectional studies link α-diversity, specific genera, and stool metabolomic profiles to internalizing and related dimensions; cluster-based approaches also identify configurations enriched in *Bifidobacterium* or *Bacteroides* that differ in adaptive skills and social functioning^17,18^. Parallel work in high-risk groups suggests that very-low-birth-weight and preterm infants with early dysbiosis show altered cognitive and CBCL-based behavioral profiles, and that children with autism spectrum disorder or attention-deficit/hyperactivity disorder exhibit distinct gut microbial signatures, some of which transfer autism- or hyperactivity-like behaviors to germ-free mice^8,9,19^. Nevertheless, pediatric studies have largely focused on global internalizing or externalizing scores, categorical diagnoses, or selected high-risk populations, and only a few have systematically related microbial diversity, genus-level composition, and predicted functions to multiple CBCL 1½–5 syndrome scales in non-referred preschool children. Functional resolution is also limited: although recent preschool work integrated 16S profiling with stool metabolomics to define microbiota clusters that differ in behavioral outcomes^17^, predicted metabolic pathways have rarely been tested against specific domains such as anxious/depressed, withdrawn, somatic complaints, sleep problems, attention problems, and aggression. This gap matters because adult studies suggest that pathway-level features show more reproducible associations with depressive symptomatology than individual taxa^10,11^.

Most existing cohorts are from Europe or North America, and Japanese preschool children remain relatively understudied despite cultural and dietary factors that may shape both the gut microbiota and early behavior. In Japanese children aged 3–4 years, temperament assessed with the Children’s Behavior Questionnaire was associated with gut microbiota composition and diversity, and higher negative affectivity was related to lower Faecalibacterium and higher *Eggerthella* and *Flavonifractor*^20^. Another Japanese preschool study reported that children at risk for explicit emotion regulation difficulties showed higher relative abundance of *Actinomyces* and *Sutterella* than non-risk children^21^. Building on this evidence, the present study examined whether gut microbial diversity, genus-level composition, and predicted gut microbiome functions are associated with anthropometric measures and the full set of CBCL 1½–5 syndrome scales in a community sample of Japanese preschool children.

## Methods

### Ethics approval and consent to participate

The study protocol was approved by the Ethics Committee of the Faculty of Education, Mie University (approval No. 2023–02). All methods were performed in accordance with the relevant guidelines and regulations and in accordance with the Declaration of Helsinki. Written informed consent was obtained from the parents or legal guardians of all participating children.

### Participants and stool collection

To examine associations between gut microbiota, physical growth, and behavioral development in early childhood, we collected stool samples from 36 preschool children living in Mie, Okayama, and Shiga Prefectures. Stool samples were collected at home by guardians using a fecal sampling kit containing preservation buffer for gut microbiota analysis (TechnoSuruga Laboratory, Shizuoka, Japan). According to the manufacturer’s instructions, the preservation solution is designed to maintain fecal microbial community profiles for approximately 1 month at room temperature (1–30 °C), and ambient-temperature transport is permitted. Guardians mailed the samples to the laboratory, where they were frozen at − 20 °C immediately upon receipt and stored until DNA extraction. The exact interval from stool collection to laboratory freezing was not recorded for each participant; however, based on the collection and mailing workflow, this interval was expected to be approximately 2–3 days.

### Behavioral assessment of preschool children

Guardians reported each child’s sex, date of birth, and completed the Child Behavior Checklist for Ages 1½–5 (CBCL 1½–5), a parent-report questionnaire within the Achenbach System of Empirically Based Assessment (ASEBA). The CBCL 1½–5 comprises 100 items that are rated 0 (“Not true”), 1 (“Somewhat or sometimes true”), or 2 (“Very true or often true”) and are grouped into seven empirically derived syndrome scales: Emotionally Reactive, Anxious/Depressed, Withdrawn, Somatic Complaints, Attention Problems, Aggressive Behavior, and Sleep Problems. In this study, we used the Japanese version of the CBCL 1½–5, which has demonstrated adequate reliability and validity^22^. A detailed list of questionnaire items is provided in Table S1. Item scores were summed to yield raw scores for each syndrome and the broadband Internalizing, Externalizing, and Total Problems scales. Raw syndrome scores were used both to describe normative, borderline, and clinical ranges and as continuous variables in correlation analyses, in line with Japanese CBCL 1½–5 manuals.

### Microbiome profiling and statistical analysis

Stool DNA was extracted using the NucleoSpin® DNA Stool kit (Macherey‑Nagel, Düren, Germany) with bead‑beating. The V3–V4 region of the bacterial 16S rRNA gene was amplified and sequenced on an Illumina MiSeq platform using paired-end 2 × 300 bp reads. Reads were processed in QIIME 2 (v2024.2) with DADA2 to generate amplicon sequence variants (ASVs), and taxonomy was assigned against the EzBioCloud 16S database. Functional profiles were predicted from ASVs using PICRUSt2 (v2.5.2) to obtain MetaCyc pathway abundances^23^. Associations between anthropometrics, CBCL syndrome scores, and microbiome features (α-diversity indices, genus-level relative abundance, and predicted MetaCyc pathways) were evaluated using Spearman’s rank correlation. To address multiple testing, raw p values from the full genus–trait correlation matrix and the full predicted pathway–trait correlation matrix were adjusted using the Benjamini–Hochberg false discovery rate method. Representative scatterplots were generated for the genus-level and predicted pathway-level associations listed in Tables 1 and 2. To assess whether these representative correlations were driven by individual observations, leave-one-out Spearman correlation analyses were performed by recalculating Spearman’s ρ after excluding one participant at a time. Detailed laboratory procedures and bioinformatic parameters are provided in Supplementary Methods.

**Table 1:** Representative gut microbial genera nominally associated with physical characteristics and CBCL 1½–5 behavioral measures in preschool children.

Trait	Taxonomy	Spearman’s correlation coefficient (ρ)	Significance level (p-value)	Direction
Age	Enterobacteriaceae ;_	0.482	< 0.01	Positive
Age	*Haemophilus*	0.468	< 0.01	Positive
Age	*Hungatella*	-0.428	< 0.01	Negative
Age	*Escherichia*	-0.481	< 0.01	Negative
Age	*Clostridium_g6*	-0.535	< 0.01	Negative
Height	*Coprococcus_g2*	-0.340	< 0.05	Negative
Height	*Caproiciproducens*	-0.355	< 0.05	Negative
Weight	*Caproiciproducens*	-0.337	< 0.05	Negative
Weight	PAC000195_g	-0.340	< 0.05	Negative
Emotionally reactive	*Coprobacter*	0.410	< 0.05	Positive
Anxious/Depressed	*Haemophilus*	0.385	< 0.05	Positive
Anxious/Depressed	*Subdoligranulum*	-0.391	< 0.05	Negative
Somatic complaints	*Ruminococcus_g4*	0.400	< 0.05	Positive
Withdrawn	*Eisenbergiella*	0.546	< 0.01	Positive
Withdrawn	*Barnesiella*	0.517	< 0.01	Positive
Withdrawn	*Fusobacterium*	-0.409	< 0.05	Negative
Sleep problems	PAC000195_g	0.340	< 0.05	Positive
Sleep problems	*Megamonas*	-0.369	< 0.05	Negative
Attention problems	PAC000195_g	0.433	< 0.01	Positive
Aggressive behavior	*Coprobacter*	0.538	< 0.01	Positive
Aggressive behavior	PAC000195_g	0.462	< 0.01	Positive

This table presents representative genus-level nominal associations selected from the full results in Table S5 based on raw p value, mean relative abundance, effect size, and representation of growth-related traits and CBCL domains.

**Table 2:** Representative predicted gut microbiome functions nominally associated with physical and behavioral traits in preschool children.

Trait	Function	Spearman’s correlation coefficient (ρ)	Significance level (p-value)	Direction
Age	L-leucine degradation I	0.571	< 0.01	Positive
Age	adenosylcobalamin biosynthesis I (anaerobic)	0.564	< 0.01	Positive
Age	protocatechuate degradation II (ortho-cleavage pathway)	0.506	< 0.01	Positive
Height	L-arginine biosynthesis III (via N-acetyl-L-citrulline)	0.356	< 0.05	Positive
Height	succinate fermentation to butanoate	-0.486	< 0.01	Negative
Weight	L-arginine biosynthesis III (via N-acetyl-L-citrulline)	0.425	< 0.05	Positive
Weight	succinate fermentation to butanoate	-0.470	< 0.01	Negative
Emotionally reactive	superpathway of pyrimidine deoxyribonucleotides de novo biosynthesis	0.442	< 0.01	Positive
Emotionally reactive	superpathway of histidine, purine, and pyrimidine biosynthesis	0.380	< 0.05	Positive
Emotionally reactive	allantoin degradation IV (anaerobic)	-0.361	< 0.05	Negative
Anxious/depressed	superpathway of pyrimidine deoxyribonucleotides de novo biosynthesis	0.440	< 0.01	Positive
Anxious/depressed	superpathway of histidine, purine, and pyrimidine biosynthesis	0.378	< 0.05	Positive
Somatic complaints	aerobic respiration I (cytochrome c)	-0.401	< 0.05	Negative
Somatic complaints	cob(II)yrinate a,c-diamide biosynthesis I (early cobalt insertion)	-0.395	< 0.05	Negative
Somatic complaints	L-tyrosine degradation I	-0.387	< 0.05	Negative
Withdrawn	superpathway of 2,3-butanediol biosynthesis	-0.433	< 0.01	Negative
Withdrawn	heterolactic fermentation	-0.409	< 0.05	Negative
Withdrawn	Bifidobacterium shunt	-0.406	< 0.05	Negative
Sleep problems	L-methionine biosynthesis III	0.473	< 0.01	Positive
Sleep problems	L-methionine biosynthesis I	0.470	< 0.01	Positive
Sleep problems	superpathway of S-adenosyl-L-methionine biosynthesis	0.463	< 0.01	Positive
Sleep problems	superpathway of heme b biosynthesis from uroporphyrinogen-III	0.463	< 0.01	Positive
Attention problems	GDP-D-glycero-α-D-manno-heptose biosynthesis	0.434	< 0.01	Positive
Attention problems	superpathway of fucose and rhamnose degradation	-0.497	< 0.01	Negative
Attention problems	L-fucose degradation I	-0.435	< 0.01	Negative
Aggressive behavior	superpathway of heme b biosynthesis from uroporphyrinogen-III	0.461	< 0.01	Positive
Aggressive behavior	tRNA processing	0.404	< 0.05	Positive
Aggressive behavior	ADP-L-glycero-β-D-manno-heptose biosynthesis	0.379	< 0.05	Positive

This table presents representative predicted pathway-level nominal associations selected from the full results in Table S6 based on raw p value, mean relative frequency, effect size, and representation of the main functional themes for each trait.

As a sensitivity analysis for potential antibiotic-related confounding, parental-reported medication history was reviewed, systemic antibacterial agents including oral antibiotics were coded as systemic antibiotic exposure, and the main Spearman correlation analyses shown in Tables 1 and 2 were repeated after excluding children with reported systemic antibiotic exposure.

## Results

### Physical characteristics and behavioral domains measured by CBCL 1½–5

**Fig. 1:**
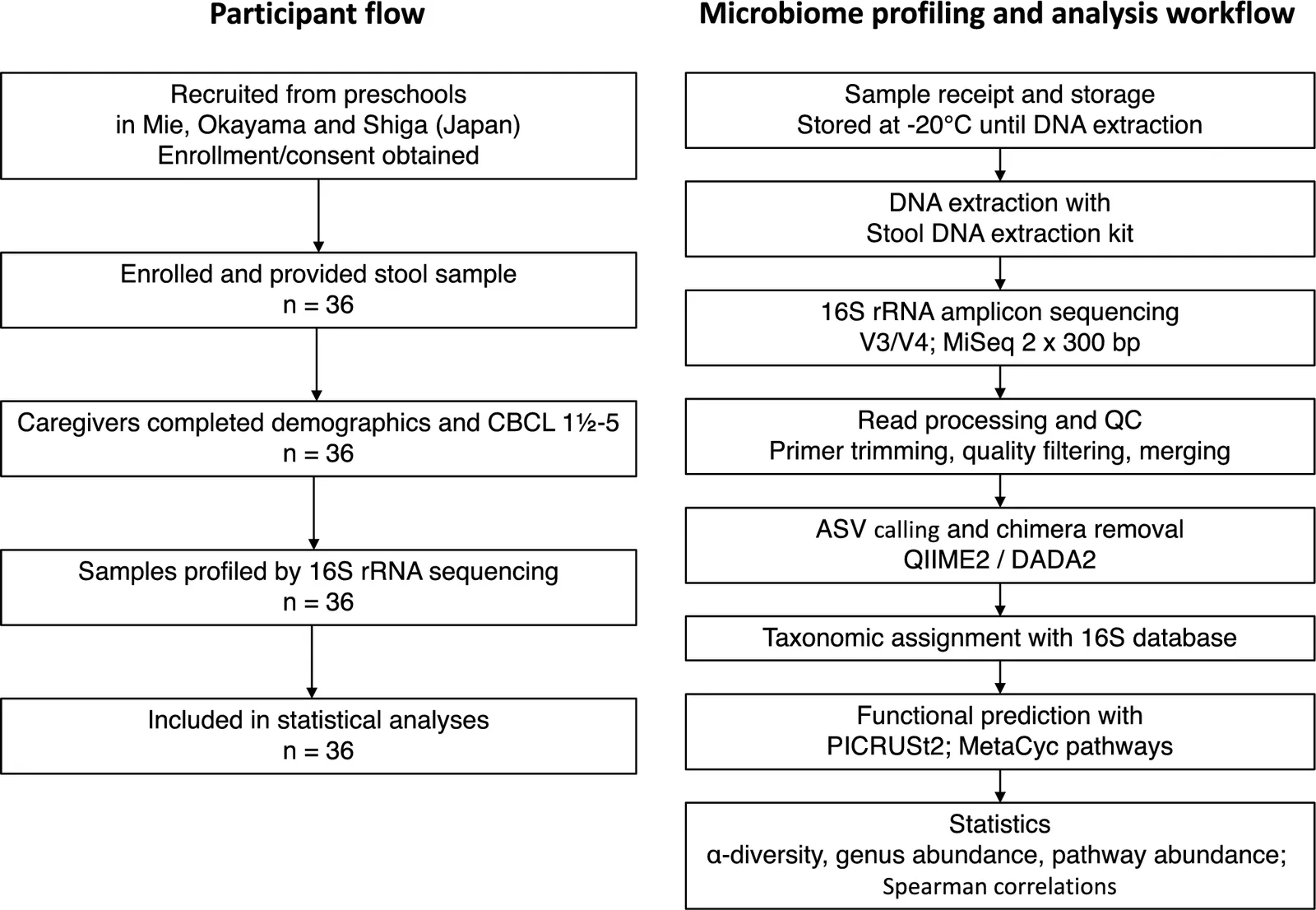
Participant flow diagram and microbiome analysis workflow.

A total of 36 children completed the CBCL 1½–5 (Fig. 1, Table S2). Twenty were boys and sixteen were girls. The mean age was 48.0 ± 8.2 months. Mean body size was 99.0 ± 5.2 cm in height (range 86.5–112.0 cm) and 15.6 ± 1.8 kg in weight (range 12.5–21.0 kg). Most children scored within the normative range across CBCL domains. Borderline elevations were most common for Internalizing Problems (7/36, 19.4%) and Sleep Problems (5/36, 13.9%). Clinically significant scores were observed most frequently for Externalizing Problems (6/36, 16.7%), followed by Total Problems (4/36, 11.1%). No participant met the clinical threshold for Anxious/Depressed, Withdrawn, or Attention Problems (Table S2).

**Fig. 2:**
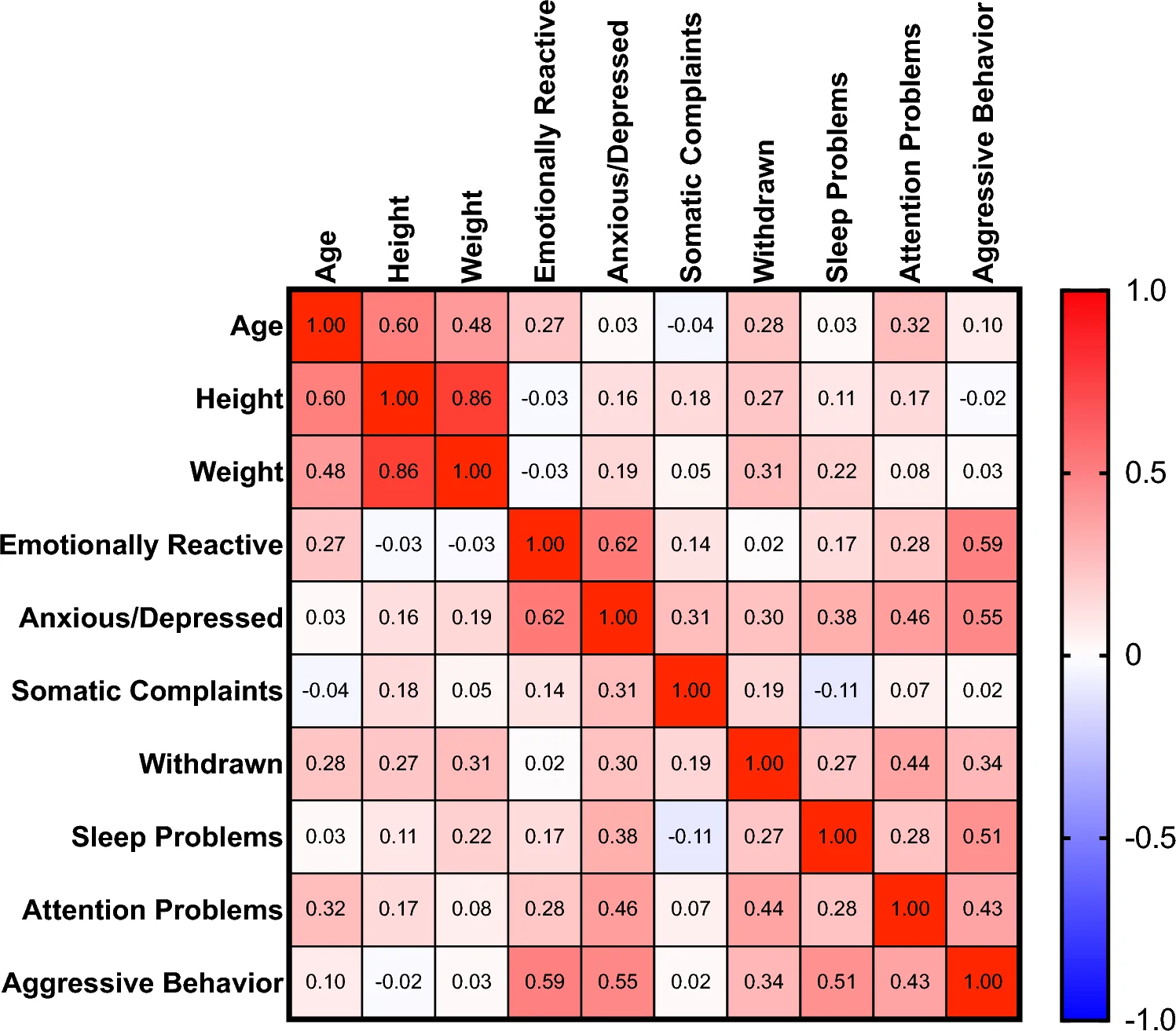
Correlation matrix of physical characteristics and CBCL 1½–5 syndrome scores in preschool children.

We used Spearman’s rank correlations to examine associations among age, height, weight, and CBCL domains (Fig. 2). Height and weight were strongly correlated (ρ = 0.86, p < 0.001). Age correlated positively with height (ρ = 0.60, p < 0.001) and with weight (ρ = 0.48, p = 0.003). Aggressive Behavior correlated with Emotionally Reactive (ρ = 0.59, p < 0.001) and with Anxious/Depressed (ρ = 0.55, p = 0.001), suggesting co-occurrence of aggression, emotional dysregulation, and internalizing symptoms. Emotionally Reactive correlated with Anxious/Depressed (ρ = 0.62, p < 0.001). Anxious/Depressed also correlated with Attention Problems (ρ = 0.46, p = 0.005) and Sleep Problems (ρ = 0.38, p = 0.023), suggesting shared underlying mechanisms among internalizing, attentional, and sleep domains. Withdrawn correlated with Attention Problems (ρ = 0.44, p = 0.007) and Aggressive Behavior (ρ = 0.34, p = 0.040), indicating that social withdrawal could co-occur with both attentional and externalizing features. In contrast, the Somatic Complaints scale did not demonstrate any statistically significant correlations with physical measures or other behavioral domains. Overall, CBCL domains were interrelated, whereas associations between growth indices and behavioral traits were comparatively modest.

### Nominal association between attention problems and gut microbiota α-diversity

α-Diversity indices were not associated with age, height, or weight. At the nominal p < 0.05 level, Attention Problems correlated positively with observed features (ρ = 0.34, p = 0.046); Chao1 richness (ρ = 0.34, p = 0.043); and Faith’s phylogenetic diversity (ρ = 0.39, p = 0.018) (Table S3). No other syndrome scale correlated with α-diversity indices, including Emotionally Reactive, Anxious/Depressed, Somatic Complaints, Withdrawn, Sleep Problems, and Aggressive Behavior. These effects were modest and should be interpreted cautiously given the sample size, but they suggest that within-sample richness and phylogenetic diversity may align more closely with attention-related variation than with other behavioral domains or growth indicators in this cohort.

### Associations between gut microbiota and behavioral and physical development in preschool children

In total, 362 genera were identified across 36 amplicon-based microbiome profiles (Table S4). Spearman’s rank correlations between genus-level relative abundance and 10 traits yielded 128 nominal associations at raw p < 0.05 (Table S5). However, none of these genus-level associations remained significant after Benjamini–Hochberg false discovery rate correction across the full set of genus–trait tests. Therefore, the genus-level findings are presented as exploratory nominal associations rather than FDR-supported discoveries. Table 1 presents representative genus-level nominal associations selected from Table S5. We prioritized taxa with mean relative abundance ≥ 0.10% and relatively large effect sizes (|ρ|≥ 0.40), while also including additional nominal associations with smaller effect sizes to provide representative coverage of growth-related traits and CBCL domains. Representative scatterplots, full raw p values, and leave-one-out Spearman’s ρ ranges are provided in Fig. S1 to allow visual assessment of whether the displayed associations reflected broad sample-level trends or were driven by individual observations.

### Age

With age, *Escherichia* decreased (mean = 2.1%, ρ = –0.48, p < 0.01), and *Clostridium*_g6 also decreased (0.4%, ρ = –0.54, p < 0.01). In contrast, *Haemophilus* increased (1.3%, ρ = 0.47, p < 0.01), Enterobacteriaceae ;_ increased (0.14%, ρ = 0.48, p < 0.01), and *Hungatella* decreased (0.32%, ρ = –0.43, p < 0.01) (Table 1).

### Height and weight

Height correlated negatively with *Coprococcus*_g2 (1.71%, ρ = –0.34, p < 0.05) and *Caproiciproducens* (0.40%, ρ = –0.36, p < 0.05). Both taxa have been described as producers of butyrate and hexanoate from complex polysaccharides^24^. Weight correlated negatively with *Caproiciproducens* (0.40%, ρ = –0.34, p < 0.05) and with PAC000195_g (0.14%, ρ = –0.34, p < 0.05). These correlations do not establish causality in this cross-sectional cohort, but they suggest lower relative abundance of these fiber-fermenting taxa in taller and heavier children.

### Anxious/depressed

Anxious/Depressed scores were positively associated with *Haemophilus* (1.26%, ρ = 0.39, p < 0.05) and negatively with *Subdoligranulum* (0.96%, ρ = –0.39, p < 0.05). *Haemophilus* can utilize host-derived sialic acids and produce lipooligosaccharides that activate Toll-like receptor signaling^25,26^, whereas *Subdoligranulum* has been implicated in supporting mucosal barrier integrity^11,27^. This reciprocal pattern is compatible with greater inflammatory potential and reduced barrier support in children with higher Anxious/Depressed scores, although these functional inferences remain speculative without direct measurement of host inflammatory markers or microbial metabolites.

### Somatic complaints

Somatic Complaints were positively associated with *Ruminococcus*_g4 (1.91%, ρ = 0.40, p < 0.05), a taxon that ferments resistant starch to acetate and formate^28^. Formate has been proposed to influence sensory pathways involved in visceral pain^29^, but the mechanism underlying this association remains unclear.

### Withdrawn

Withdrawn showed the strongest single effect size among the trait–taxon correlations. *Eisenbergiella* (0.63%, ρ = 0.55, p < 0.01) and *Barnesiella* (0.14%, ρ = 0.52, p < 0.01) were enriched in children with higher scores. *Eisenbergiella* can generate amino-acid-derived butyrate, and *Barnesiella* harbors bile-salt hydrolases and has been linked to modulation of host immunity and social behavior^30^, suggesting potential relevance of amino-acid- and bile-acid-related microbial pathways for social withdrawal in early childhood. *Fusobacterium*, a succinate and putrescine producer, was depleted (0.23%, ρ = –0.41, p < 0.05), supporting the view that withdrawal-related variation may involve opposing shifts across microbial metabolic routes rather than uniform changes in overall short-chain fatty acid output.

### Sleep problems

Sleep Problems were inversely correlated with *Megamonas*, a propionate producer (1.69%, ρ = –0.37, p < 0.05), and positively correlated with PAC000195_g (0.14%, ρ = 0.34, p < 0.05). These contrasting associations raise the possibility that differences in the balance between propionate and mixed short-chain fatty acid production relate to variation in sleep regulation or circadian rhythms, although targeted metabolomic data would be needed to test this hypothesis.

### Emotionally reactive, attention problems, and aggressive behavior

Aggressive Behavior showed the highest correlations with *Coprobacter* (0.16%, ρ = 0.54, p < 0.01), and the Lachnospiraceae taxon PAC000195_g (0.14%, ρ = 0.46, p < 0.01). *Coprobacter* was also positively associated with Emotionally Reactive (0.16%, ρ = 0.41, p < 0.05) but not with Attention Problems, whereas PAC000195_g was positively associated with Attention Problems (0.14%, ρ = 0.43, p < 0.01) but not with Emotionally Reactive. *Coprobacter* belongs to a Barnesiellaceae lineage that often encodes bile salt hydrolase homologs, suggesting capacity to deconjugate primary bile acids. Deconjugated bile acids can cross the blood–brain barrier and engage central bile acid receptors in limbic circuits, and bile acid signaling has been linked to anxiety- and depression-like behaviors in rodent models^31^. These features provide one plausible route through which *Coprobacter*-enriched communities might be linked to heightened emotional lability and aggression, although such causal pathways were not tested here. PAC000195_g, in contrast, carries an extensive repertoire of carbohydrate esterases, suggesting a capacity to liberate small neuroactive metabolites from complex plant polysaccharides, but this mechanism and its potential relevance to inattention and impulsivity remain to be directly demonstrated in humans.

### Associations between gut microbiome functions and behavioral and physical development in preschool children

PICRUSt2 inference yielded 411 non-redundant MetaCyc pathways across the 36 fecal microbiome profiles. Spearman correlations between predicted pathway relative abundance and 10 traits identified 230 nominal associations at raw p < 0.05 (Table S6). However, none of the predicted pathway-level associations remained significant after Benjamini–Hochberg false discovery rate correction across the full set of pathway–trait tests. We therefore present these results as exploratory nominal pathway-level associations. Table 2 presents representative predicted pathway-level nominal associations selected from Table S6. We prioritized pathways with mean relative frequency ≥ 0.0010% and at least moderate effect sizes (|ρ|≥ 0.35), while selecting representative pathways that captured the main functional themes for each trait. Representative scatterplots, full raw p values, and leave-one-out Spearman’s ρ ranges are provided in Fig. S2 to allow visual assessment of whether the displayed associations reflected broad sample-level trends or were driven by individual observations.

### Age

Older preschool children showed increased predicted capacities for branched-chain amino-acid catabolism and cofactor production. The strongest signal was L-leucine degradation I (LEU-DEG2-PWY) (0.0043% mean relative frequency, ρ = 0.57, p < 0.01), followed by adenosylcobalamin biosynthesis I (PWY-5507) (0.0190%, ρ = 0.56, p < 0.01). Leucine degradation yields acetyl-CoA and acetoacetate, whereas adenosylcobalamin biosynthesis suggests increased predicted capacity for vitamin B_12_ production. Together, these patterns are consistent with enhanced microbial energy harvest and cobalamin-dependent metabolism with age. Protocatechuate degradation II (PROTOCATECHUATE-ORTHO-CLEAVAGE-PWY) (0.0019%, ρ = 0.51, p < 0.01) also increased with age, indicating a parallel expansion of polyphenol breakdown as diets diversify.

### Height and weight

Height was positively linked to amino-acid anabolism rather than fermentation. L-arginine biosynthesis III via N-acetyl-L-citrulline (PWY-5154), correlated positively (0.360%, ρ = 0.36, p < 0.05), whereas succinate fermentation to butanoate (PWY-5677) was inversely related (0.0322%, ρ = –0.49, p < 0.01). Taller children therefore tended to host microbiota with higher predicted capacity for arginine biosynthesis and lower inferred reliance on reductive succinate pathways. Body weight reproduced the height pattern for arginine biosynthesis (0.360%, ρ = 0.43, p < 0.05), and for succinate fermentation to butanoate (0.0322%, ρ = –0.47, p < 0.01). These associations suggest coordinated adjustments in predicted amino-acid and energy metabolism along gradients of somatic growth in this cohort.

### Emotionally reactive

Higher Emotionally Reactive scores coincided with enrichment of pathways related to nucleotide biosynthesis pathways. The top pathway was superpathway of pyrimidine deoxyribonucleotides de novo biosynthesis (PWY-7211) (0.287%, ρ = 0.44, p < 0.01), followed by superpathway of histidine, purine, and pyrimidine biosynthesis (PRPP-PWY) (0.315%, ρ = 0.38, p < 0.05). These associations were accompanied by a negative link to allantoin degradation IV (PWY0-41) (0.0078%, ρ = –0.36, p < 0.05). Together, this pattern is consistent with microbiota enriched in de novo nucleotide production and altered nitrogen salvage in children with higher emotional reactivity, but the functional implications for host neurotransmitter precursors remain speculative without direct metabolomic data.

### Anxious/depressed

Anxious/Depressed scores showed a similar functional profile, with positive correlations to superpathway of pyrimidine deoxyribonucleotides de novo biosynthesis (PWY-7211) (0.287%, ρ = 0.44, p < 0.01) and superpathway of histidine, purine, and pyrimidine biosynthesis (PRPP-PWY) (0.315%, ρ = 0.38, p < 0.05). No negatively correlated high-frequency pathways surpassed the thresholds, suggesting that enhanced predicted de novo nucleotide synthesis was the dominant shift accompanying anxious traits in this sample.

### Somatic complaints

Somatic Complaints were inversely associated with aerobic respiration I (cytochrome c) (PWY-3781) (0.0402%, ρ = –0.40, p < 0.05), cob(II)yrinate a,c-diamide biosynthesis I (early cobalt insertion) (PWY-7377) (0.145%, ρ = –0.40, p < 0.05), and L-tyrosine degradation I (TYRFUMCAT-PWY) (0.0014%, ρ = –0.39, p < 0.05). These signals indicate reduced predicted respiratory capacity and aromatic amino-acid catabolism in children reporting more somatic symptoms.

### Withdrawn

Withdrawn was most strongly associated with lower predicted fermentative capacity. Three high-frequency fermentation routes were inversely correlated, including superpathway of 2,3-butanediol biosynthesis (PWY-6396) (0.0151%, ρ = –0.43, p < 0.01), heterolactic fermentation (P122-PWY) (0.0818%, ρ = –0.41, p < 0.05), and the *Bifidobacterium* shunt (P124-PWY) (0.0976%, ρ = –0.41, p < 0.05). This pattern suggests reduced representation of classical bifidobacterial and lactic fermentative pathways in children with higher withdrawn scores.

### Sleep problems

Sleep Problems showed the largest number of representative nominal pathway-level associations, dominated by sulfur-containing amino-acid and heme biosynthesis. Positive correlations spanned L-methionine biosynthesis III (HSERMETANA-PWY) (0.313%, ρ = 0.47, p < 0.01), L-methionine biosynthesis I (HOMOSER-METSYN-PWY) (0.283%, ρ = 0.47, p < 0.01), superpathway of S-adenosyl-L-methionine biosynthesis (MET-SAM-PWY) (0.372%, ρ = 0.46, p < 0.01), and superpathway of heme *b* biosynthesis from uroporphyrinogen-III (PWY0-1415) (0.0442%, ρ = 0.46, p < 0.01), suggesting a potential link between microbial methyl-group turnover, heme production, and sleep quality in preschool children.

### Attention problems

Higher Attention Problems scores were nominally associated with lower predicted fucose and rhamnose catabolism: superpathway of fucose and rhamnose degradation (FUC-RHAMCAT-PWY) (0.211%, ρ = –0.50, p < 0.01) and L-fucose degradation I (FUCCAT-PWY) (0.157%, ρ = –0.44, p < 0.01) were both negatively associated. Conversely, GDP-D-glycero-α-D-manno-heptose biosynthesis (PWY-6478) (0.0931%, ρ = 0.43, p < 0.01) was enriched. Together, these shifts may reflect altered microbial handling of host-derived glycans and increased heptose-nucleotide sugar biosynthesis, suggesting changes in bacterial cell-surface glycoconjugate metabolism in children with higher attention problem scores.

### Aggressive behavior

Aggressive Behavior showed nominal positive correlations with predicted pathways related to porphyrin metabolism and core RNA handling. Representative pathways included superpathway of heme *b* biosynthesis from uroporphyrinogen-III (PWY0-1415) (0.0442%, ρ = 0.46, p < 0.01), tRNA processing (PWY0-1479) (0.0981%, ρ = 0.40, p < 0.05), and ADP-L-glycero-β-D-manno-heptose biosynthesis (PWY0-1241) (0.0736%, ρ = 0.38, p < 0.05). These nominal associations are compatible with, but do not establish, differences in predicted redox-related, RNA-processing, and cell-envelope-associated metabolism in children with higher aggression scores.

### Sensitivity analysis excluding children with reported systemic antibiotic exposure

Parental-reported medication history was reviewed to identify systemic antibiotic exposure. Because some entries included non-antibacterial medications or topical agents, only systemic antibacterial agents, including oral antibiotics, were coded as systemic antibiotic exposure. Six of the 36 children were classified as having systemic antibiotic exposure (Table S2). To assess whether these children drove the main microbiome associations, we repeated the Spearman correlation analyses for the associations shown in Tables 1 and 2 after excluding these 6 children.

For the representative genus-level nominal associations shown in Table 1, all 21 associations retained the same direction in the remaining 30 children, and 15 of 21 remained nominally significant at p < 0.05. For the representative predicted MetaCyc pathway-level nominal associations shown in Table 2, all 28 associations retained the same direction, and 23 of 28 remained nominally significant at raw p < 0.05. These findings suggest that the representative nominal associations were not primarily driven by children with reported systemic antibiotic exposure, although they should not be interpreted as FDR-supported discoveries.

## Discussion

### Gut microbiota along physical growth trajectories

Age, height, and weight were strongly intercorrelated, but their correlations with CBCL domain scores were modest (Fig. 2), suggesting that behavioral variation was not a simple proxy for somatic development. Somatic indices nevertheless tracked canonical microbiota maturation and predicted functions. The decline of Escherichia and other facultative anaerobes with age, together with increasing representation of strictly anaerobic genera, mirrors cohort data describing the transition toward a more stable, adult-like microbiota during the preschool years^32^. Age-related enrichment of leucine degradation and adenosylcobalamin biosynthesis suggests increasing microbial contributions to branched-chain amino-acid catabolism and vitamin B_12_ availability as diets diversify. Height and weight were positively associated with arginine biosynthesis and inversely associated with succinate-to-butanoate fermentation, pointing to a shift away from reductive succinate pathways in larger children. The weak coupling between growth indices and CBCL traits supports the interpretation that the domain-specific signatures below are unlikely to be simple proxies of maturation.

### Overview of microbiota–behavior associations in a community sample

In this cross-sectional study of typically developing Japanese preschool children, we observed nominal genus-level and predicted pathway-level correlation patterns between gut microbiome features and multiple CBCL 1½–5 domains. These associations did not survive FDR correction and should therefore be interpreted as exploratory and hypothesis-generating. Nevertheless, representative associations retained their direction in leave-one-out analyses, suggesting that the displayed patterns were not driven solely by any single participant (Fig. S1 and S2). Within the limits of this exploratory analysis, variation in internalizing, externalizing, and sleep-related difficulties showed candidate links with distinct taxonomic and predicted metabolic features. This extends early-life microbiota–gut–brain research beyond high-risk or clinically referred cohorts and is broadly consistent with longitudinal links between infant microbiota and later internalizing and externalizing problems, executive function, and temperament^14,16,32^. In our data, anxious/depressed symptoms and emotional reactivity clustered with taxa and pathways suggestive of higher inflammatory potential and greater biosynthetic activity, whereas somatic complaints and withdrawn behavior aligned with reduced respiratory and fermentative activity. This pattern resonates with preschool studies implicating microbial metabolic output, including short-chain fatty acid profiles, rather than diversity alone^17,18^.

### Attention and aggression: distinct externalizing–microbiota relationships

Attention problem scores were positively associated with α-diversity indices, including observed features, Chao1 richness, and Faith’s phylogenetic diversity. Case–control studies in children and adolescents with clinically diagnosed attention-deficit/hyperactivity disorder (ADHD) variably report reduced α-diversity or no consistent differences^33,34^. Our community sample captures dimensional variation rather than categorical diagnoses and suggests that higher richness can co-occur with more pronounced attentional difficulties in the preschool period. A similar direction has been reported in another Japanese preschool cohort, where higher gut microbiota diversity was associated with greater impulsivity^20^. These patterns motivate longitudinal cohorts that integrate dietary and medication histories to clarify whether α-diversity reflects transient development, confounding, or early risk.

Taxonomic and functional profiles further linked attention problems and aggressive behavior to genera with plausible neuroactive potential. *Coprobacter* and the Lachnospiraceae taxon PAC000195_g, which correlated with aggressive behavior and attention problems respectively, encode bile-salt hydrolases and carbohydrate esterases, suggesting an enhanced capacity to remodel host bile acids and complex polysaccharides. Such remodeling may increase unconjugated bile acids and other small products that influence limbic excitability and prefrontal control circuits, consistent with animal-model evidence linking bile acid and microbial metabolite signaling to hyperactivity and impulsivity^8,35^. Aggressive Behavior was associated with ADP-L-glycero-β-D-manno-heptose biosynthesis (PWY0-1241). This pathway generates ADP-heptose, an inner-core lipopolysaccharide precursor that can activate intracellular innate immune signaling and elevate inflammatory tone^36^.

### Microbial signatures of internalizing difficulties in preschool children

Internalizing traits showed a complementary microbial profile. Anxious/depressed scores were positively associated with *Haemophilus* and negatively associated with *Subdoligranulum*, a sentinel butyrate producer linked to epithelial barrier support and inversely associated with depressive symptoms in adults^11,37^. *Haemophilus* can exploit host sialic acids and produce lipooligosaccharides that activate Toll-like receptor 4, promoting mucosal inflammation and systemic immune activation^25,38^. The reciprocal shift therefore supports a model in which higher anxious/depressed scores align with higher inflammatory potential and reduced barrier-supportive capacity. This interpretation is consistent with Japanese preschool findings linking emotion-regulation risk to higher *Actinomyces* and *Sutterella*^21^. Functionally, these traits aligned with enrichment of de novo pyrimidine and broader nucleotide biosynthesis pathways, compatible with more biosynthetically active communities that may influence host tryptophan, purine, and pyrimidine pools relevant to stress-response circuitry^35,39^.

Somatic complaints were associated with reduced inferred aerobic respiration and tyrosine degradation, suggesting lower respiratory flexibility and diminished aromatic amino-acid catabolism. Because aromatic amino-acid-derived metabolites, including tyrosine-derived amines, have been linked to visceral pain hypersensitivity and autonomic regulation, reduced microbial engagement with these substrates may contribute to altered interoceptive signaling^40^. Withdrawn behavior, in contrast, was associated with enrichment of *Eisenbergiella* and *Barnesiella* and depletion of *Fusobacterium*, together with reduced representation of classical fermentative pathways such as the *Bifidobacterium* shunt and heterolactic fermentation. These findings are consistent with reports linking lower bifidobacterial activity and altered amino-acid fermentation to social withdrawal and suggest that shifts in amino-acid-derived short-chain fatty acids may be relevant for social engagement in early childhood^41^.

### Microbiome functional pathways associated with sleep problems

Sleep problems showed the broadest functional footprint, with positive correlations to microbial methionine and S-adenosyl-methionine biosynthesis and to heme production pathways. These processes regulate methyl-group availability and redox-active cofactor synthesis, which can influence circadian clock machinery, melatonin production, and oxidative stress responses. Recent studies link sleep duration and efficiency in preschoolers to microbiota profiles and to microbial genes involved in amino-acid and cofactor metabolism^42,43^. Our data add that sleep disturbance aligns with enrichment of sulfur-containing amino-acid biosynthesis and porphyrin pathways, supporting pathway-level candidates for future mechanistic and interventional work.

### Methodological considerations and future directions

These findings should be interpreted as associative rather than causal. The modest sample size limits statistical power and may reduce generalizability beyond Japanese community-dwelling preschool children, and the cross-sectional design cannot determine whether microbiome differences precede behavioral traits, reflect shared environments, or arise through bidirectional feedback. Detailed information on diet, probiotic or prebiotic use, household environment, socioeconomic factors, and other lifestyle variables was not comprehensively collected and therefore could not be included as covariates in adjusted models. Because these factors can influence both gut microbiome composition and child behavioral development, residual confounding cannot be excluded. Larger longitudinal cohorts with repeated sampling and direct multi-omic measurements will be required to test directionality and mechanism.

The pathway-level findings should also be interpreted with caution because microbial functions were inferred from 16S rRNA gene profiles using PICRUSt2 rather than measured directly by whole-metagenome shotgun sequencing. PICRUSt2 provides a useful framework for estimating community functional potential from marker-gene data, but its predictions depend on reference genome coverage, phylogenetic placement, strain-level gene-content variation, primer coverage, and pathway annotation^23^. Therefore, the MetaCyc pathway associations reported here should be regarded as exploratory, hypothesis-generating signals rather than direct evidence of pathway activity.

Across domains, signals converged on microbial capacities related to nucleotide synthesis, amino-acid and methyl-group metabolism, bile-acid and glycan transformation, and heptose-containing lipopolysaccharide biosynthesis. These pathways plausibly interface with neurodevelopment by shaping metabolite availability, epithelial and immune tone, and neuroendocrine signaling relevant to emotional regulation, attention, and sleep. Future work that combines repeated stool sampling with detailed diet and medication histories and concurrent measurement of relevant metabolites and host markers can test whether microbial methyl-donor metabolism, bile-acid remodeling, and glycan utilization mediate changes in sleep and behavior over time. If replicated, these signatures could support microbiome-informed prevention studies in early childhood, including pragmatic trials of dietary modulation and targeted prebiotic or probiotic approaches, with outcomes anchored to standardized behavioral scales and objective sleep measures. Over the longer term, microbiome-based markers could complement psychosocial and educational strategies by adding a biologically grounded component to early risk monitoring and support.

## Supplementary Information

Supplementary Information 1.

Supplementary Information 2.

Supplementary Information 3.

Supplementary Information 4.

Supplementary Information 5.

Supplementary Information 6.

Supplementary Information 7.

Supplementary Information 8.

Supplementary Information 9.

## Data Availability

Raw 16S rRNA gene sequencing data and associated metadata have been deposited in the NCBI Sequence Read Archive (SRA) under accession number PRJNA1392820. Processed feature tables, taxonomic assignments, and analysis scripts are available from the corresponding author upon reasonable request.

## Abbreviations

CBCL 1½–5: Child behavior checklist for ages 1½–5
ASEBA: Achenbach system of empirically based assessment
ASV: Amplicon sequence variant
PICRUSt2: Phylogenetic investigation of communities by reconstruction of unobserved states 2
QIIME 2: Quantitative insights into microbial ecology 2
