# Supplementary material for: Gut microbiome composition and predicted functions relate to growth and behavior in a Japanese preschool cohort
Source: Sci Rep. 2026 Jun 26;16:28604. doi: 10.1038/s41598-026-59018-4 (PMC13575191; doi:10.1038/s41598-026-59018-4)
Supplement: Supplementary file 1 [file 41598_2026_59018_MOESM1_ESM.pdf]

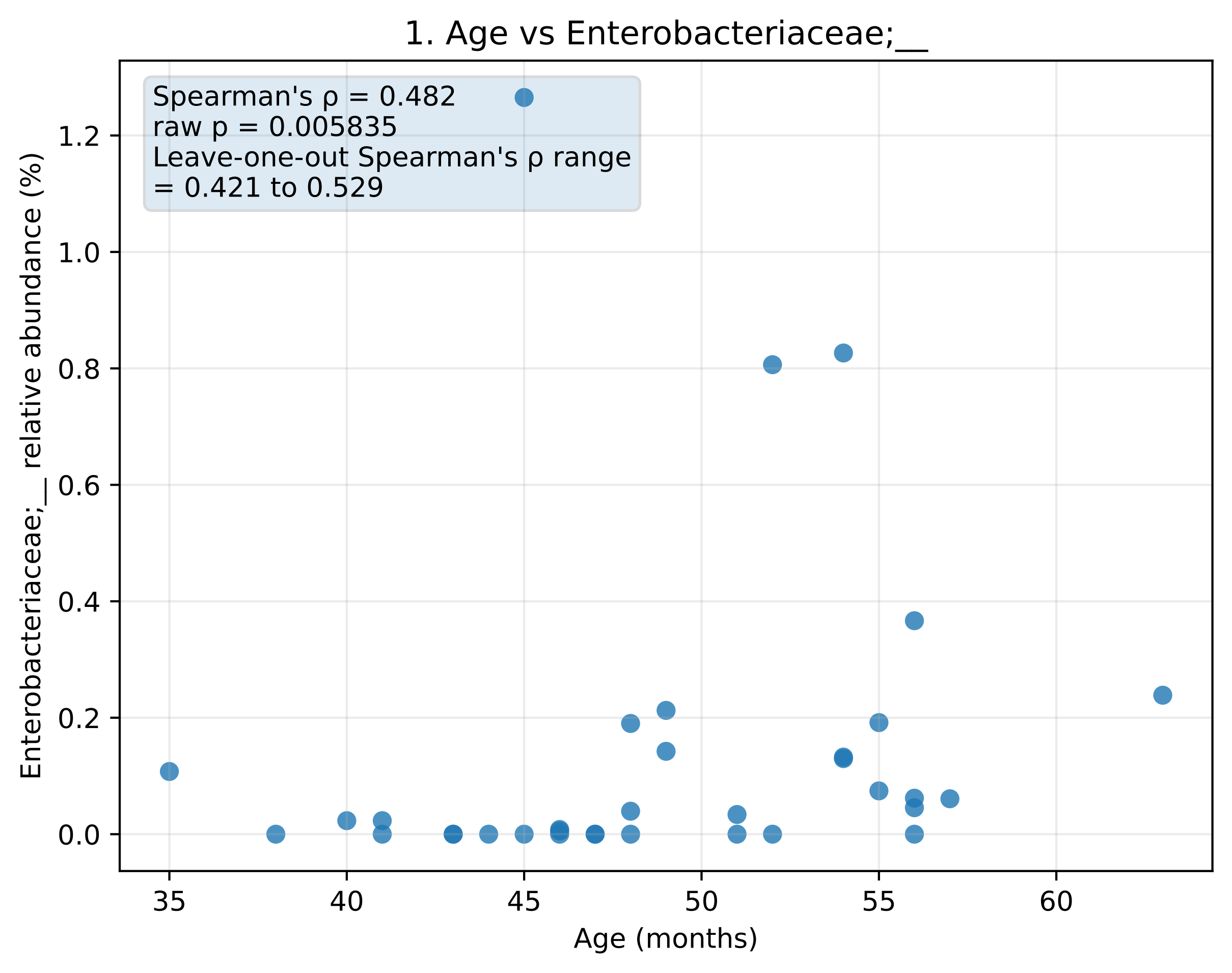
1. Age vs Enterobacteriaceae;__
Spearman's ρ = 0.482
raw p = 0.005835
Leave-one-out Spearman's ρ range
= 0.421 to 0.529
Enterobacteriaceae;__ relative abundance (%)
Age (months)
0.0
0.2
0.4
0.6
0.8
1.0
1.2
35
40
45
50
55
60

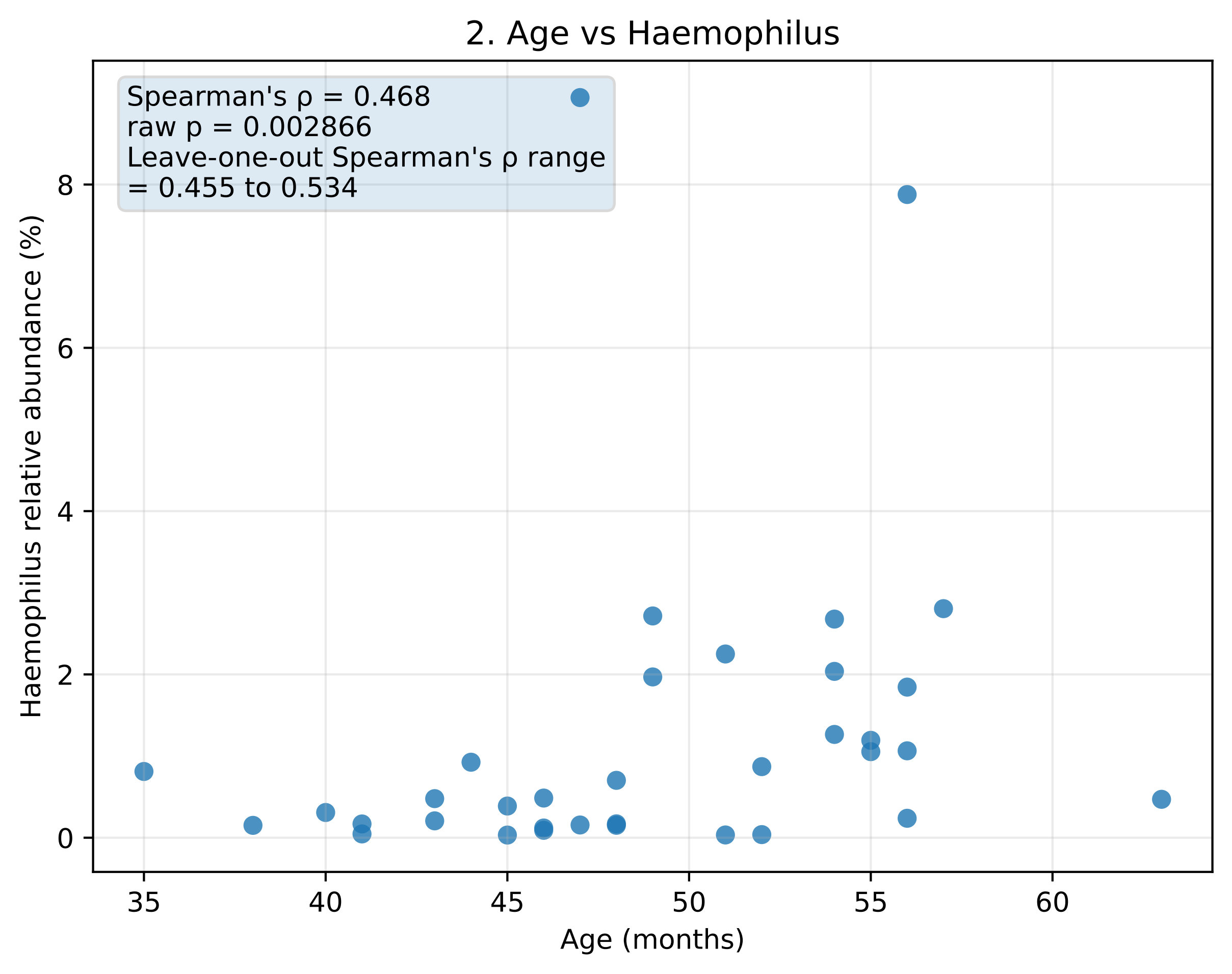
2. Age vs Haemophilus
Spearman's ρ = 0.468
raw p = 0.002866
Leave-one-out Spearman's ρ range
= 0.455 to 0.534
Haemophilus relative abundance (%)
Age (months)
0
2
4
6
8
35
40
45
50
55
60

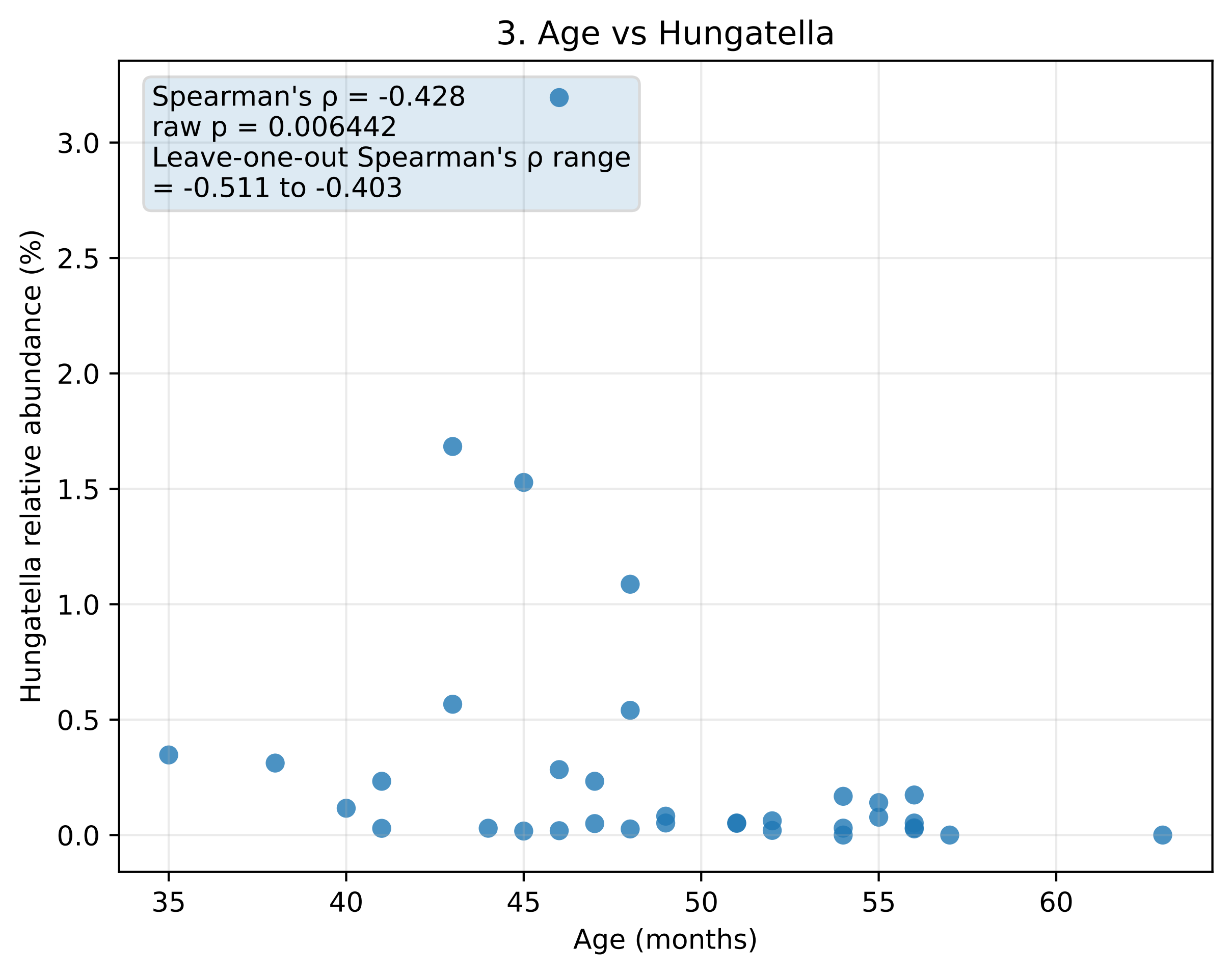
3. Age vs Hungatella
Spearman's ρ = -0.428
raw p = 0.006442
Leave-one-out Spearman's ρ range
= -0.511 to -0.403
Hungatella relative abundance (%)
Age (months)
0.0
0.5
1.0
1.5
2.0
2.5
3.0
35
40
45
50
55
60

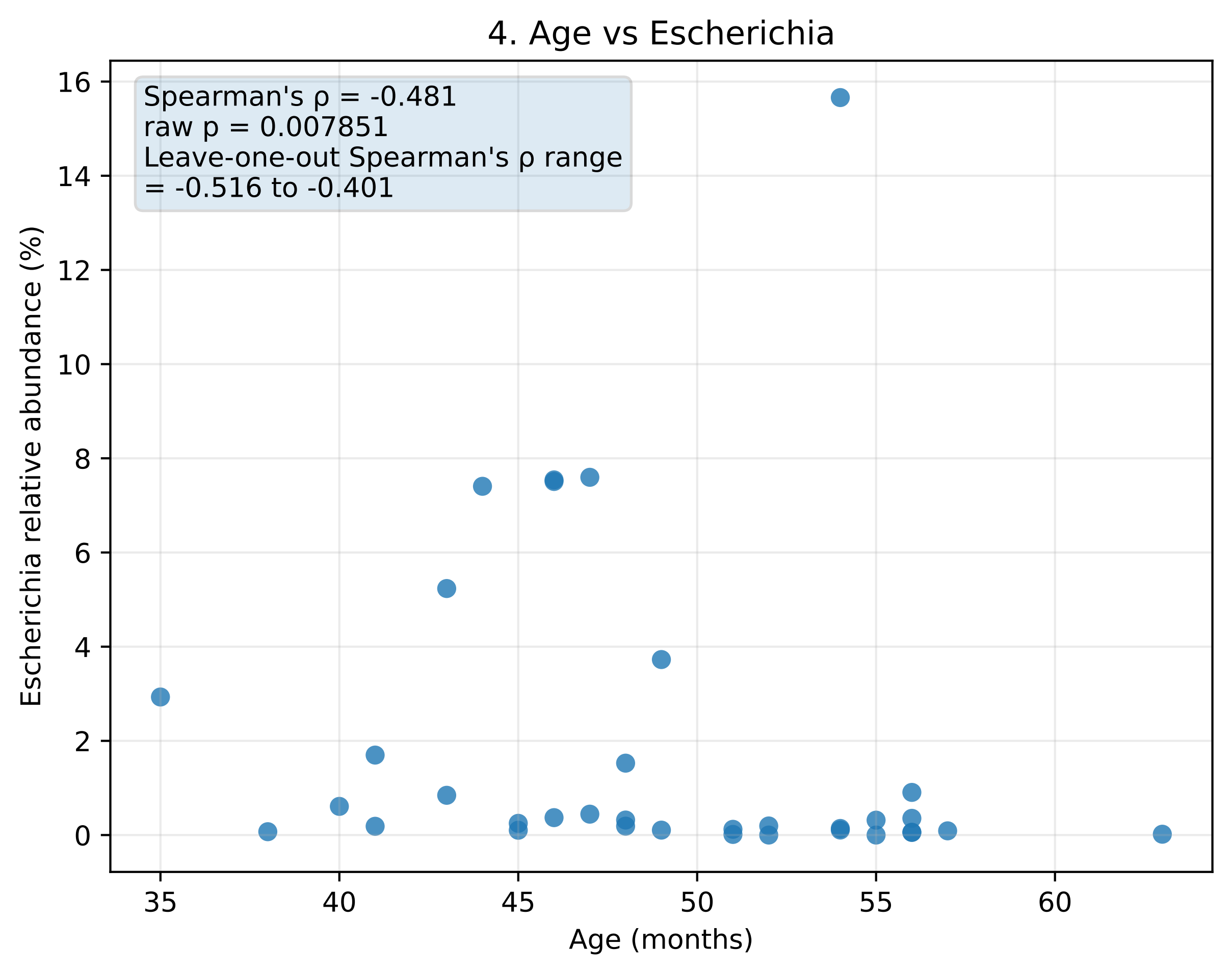
4. Age vs Escherichia
Spearman's ρ = -0.481
raw p = 0.007851
Leave-one-out Spearman's ρ range
= -0.516 to -0.401
Escherichia relative abundance (%)
Age (months)
0
2
4
6
8
10
12
14
16
35
40
45
50
55
60

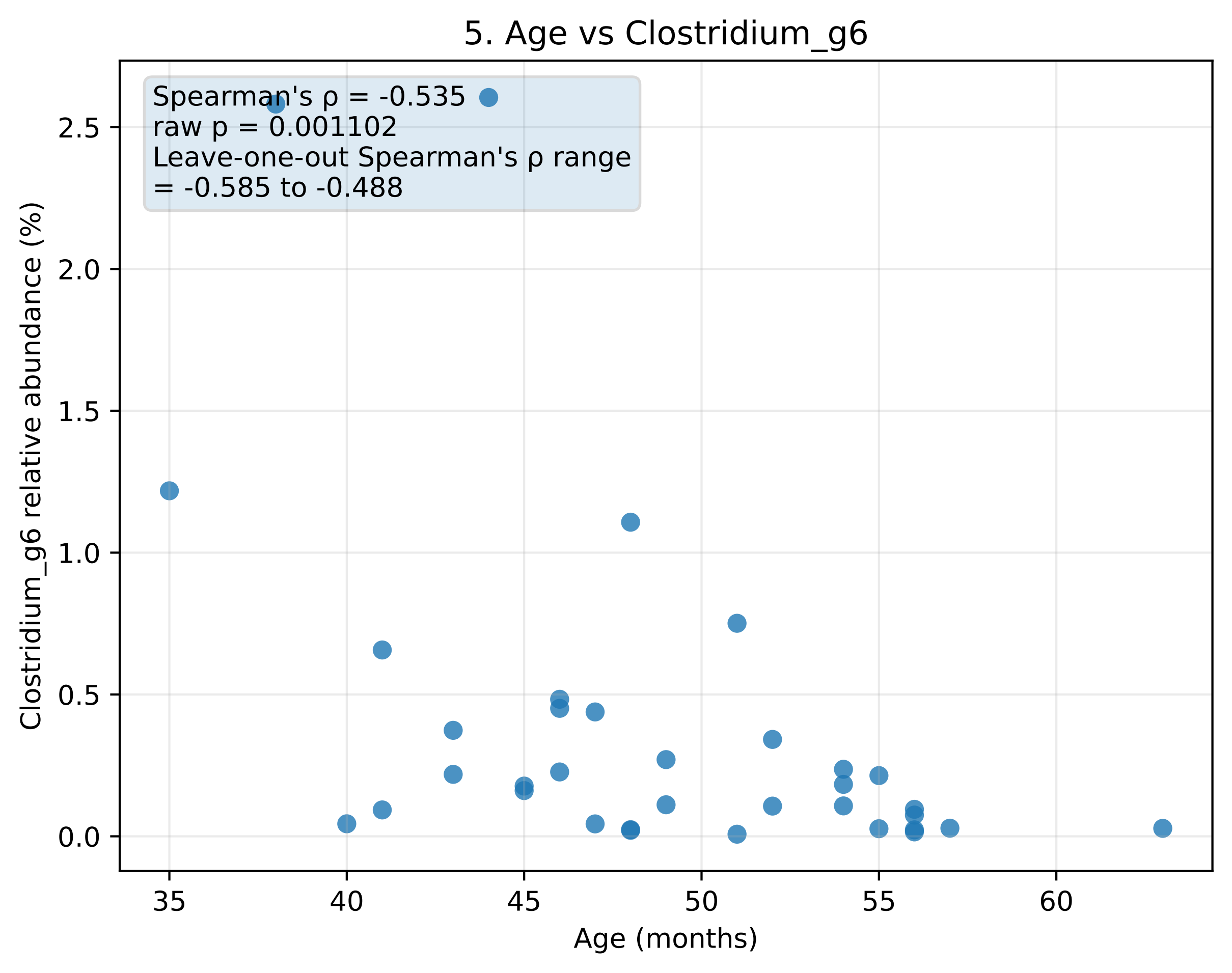
5. Age vs Clostridium_g6
Spearman's ρ = -0.535
raw p = 0.001102
Leave-one-out Spearman's ρ range
= -0.585 to -0.488
Clostridium_g6 relative abundance (%)
0.0
0.5
1.0
1.5
2.0
2.5
35
40
45
50
55
60
Age (months)

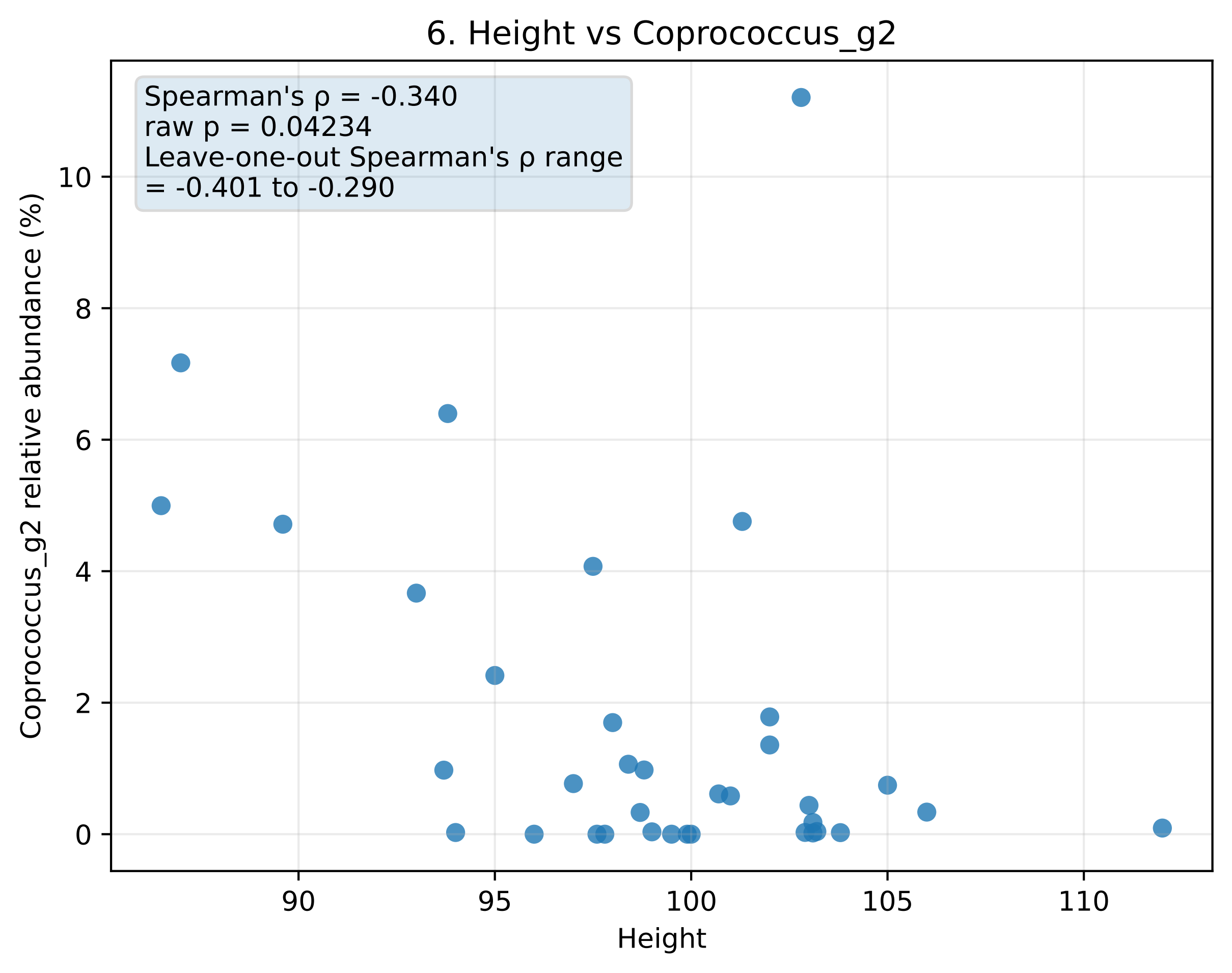
6. Height vs Coprococcus_g2
Spearman's ρ = -0.340
raw p = 0.04234
Leave-one-out Spearman's ρ range
= -0.401 to -0.290
Coprococcus_g2 relative abundance (%)
0
2
4
6
8
10
90
95
100
105
110
Height

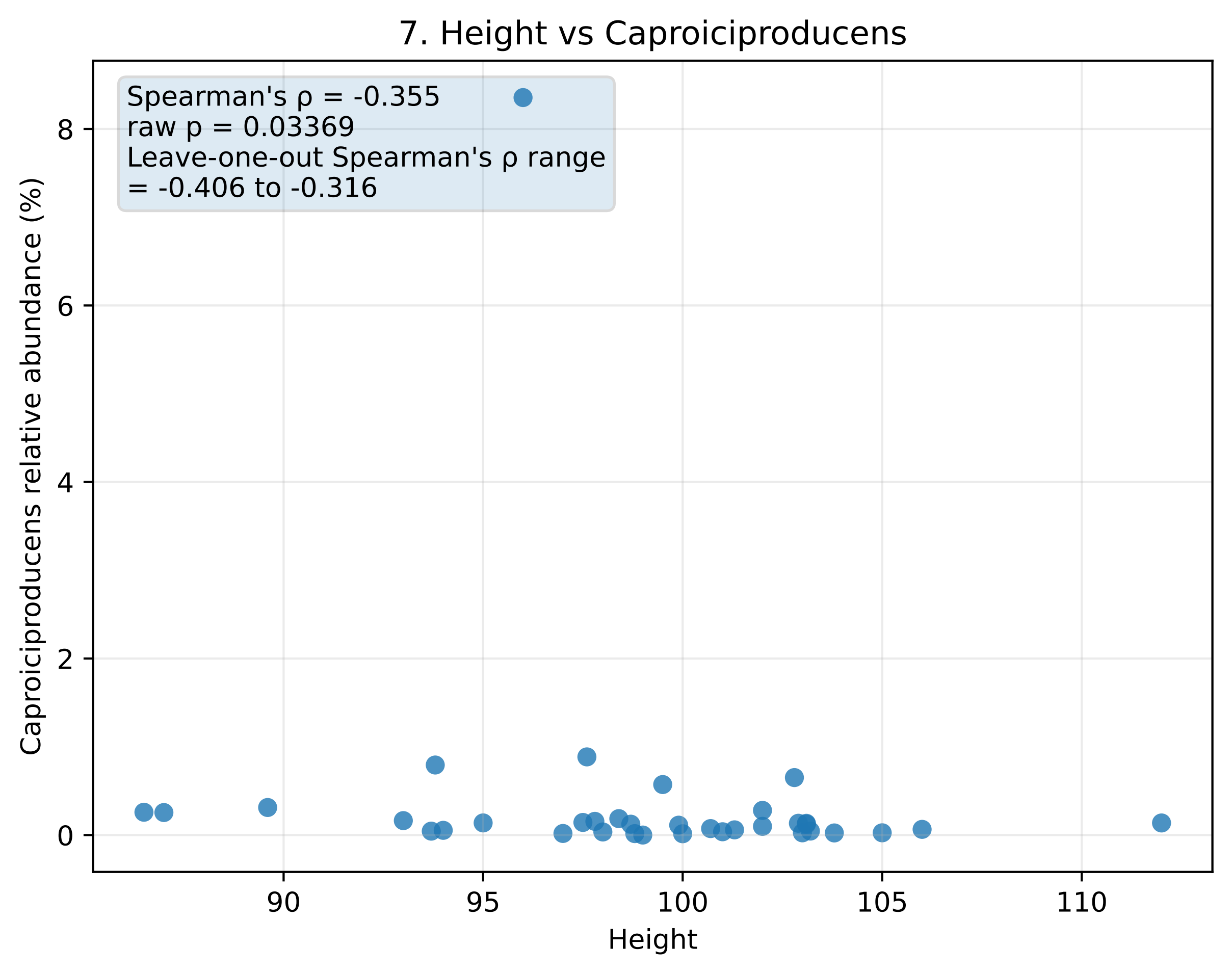
7. Height vs Caproiciproducens
Spearman's ρ = -0.355
raw p = 0.03369
Leave-one-out Spearman's ρ range
= -0.406 to -0.316
Caproiciproducens relative abundance (%)
Height
0
2
4
6
8
90
95
100
105
110

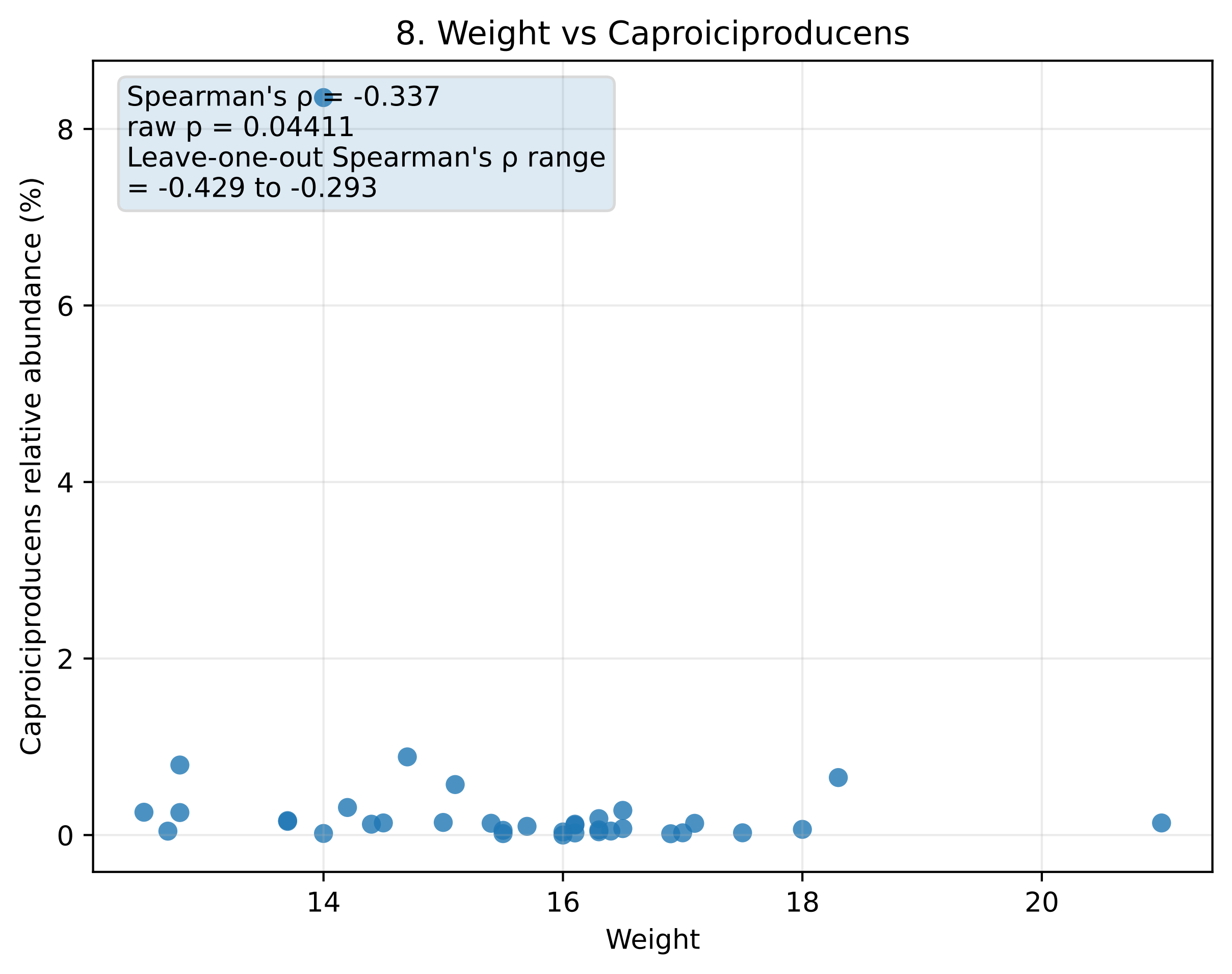
8. Weight vs Caproiciproducens
Spearman's ρ = -0.337
raw p = 0.04411
Leave-one-out Spearman's ρ range
= -0.429 to -0.293
Caproiciproducens relative abundance (%)
0
2
4
6
8
14
16
18
20
Weight

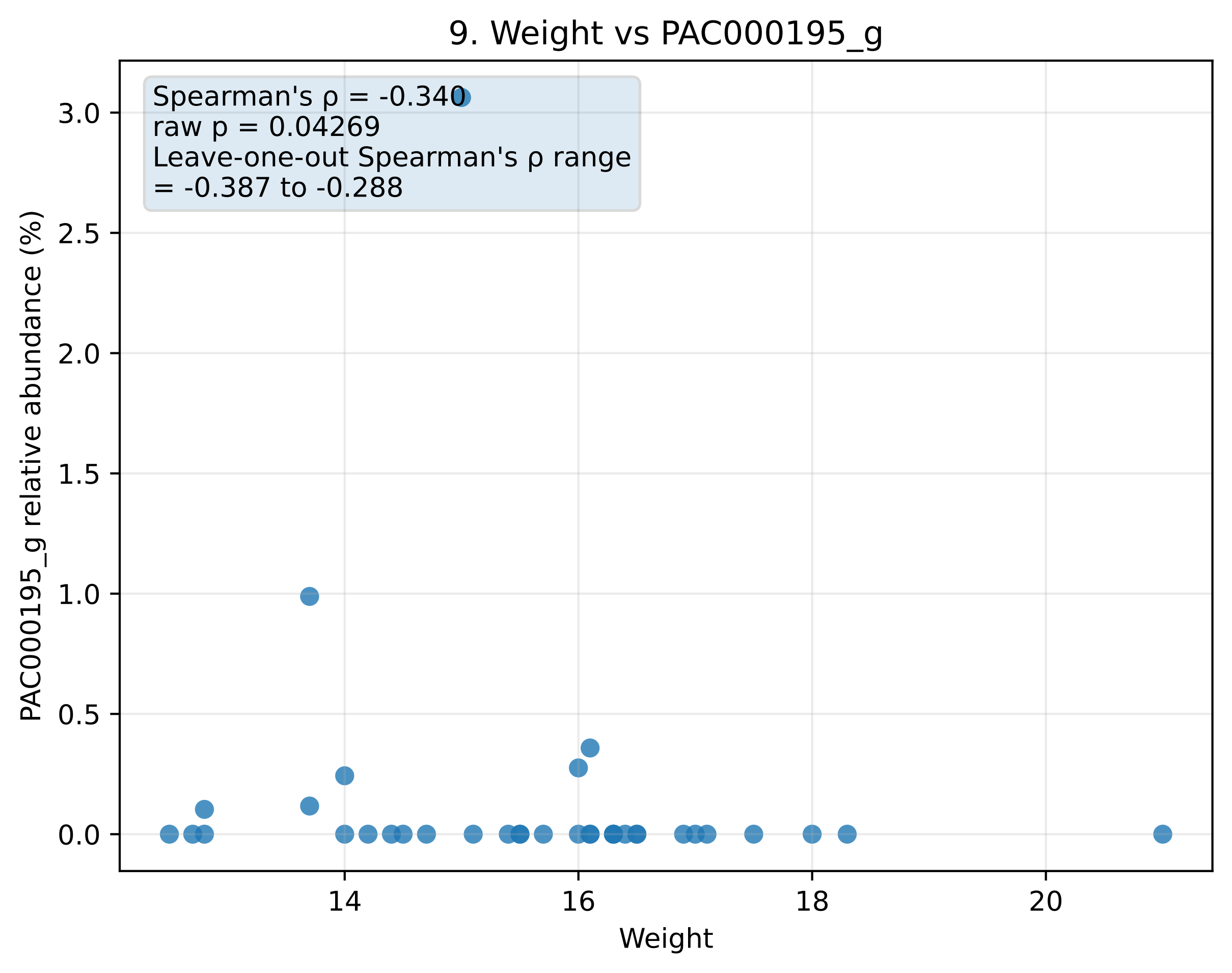
9. Weight vs PAC000195_g
Spearman's ρ = -0.340
raw p = 0.04269
Leave-one-out Spearman's ρ range
= -0.387 to -0.288
3.0
2.5
2.0
1.5
1.0
0.5
0.0
PAC000195_g relative abundance (%)
14
16
18
20
Weight

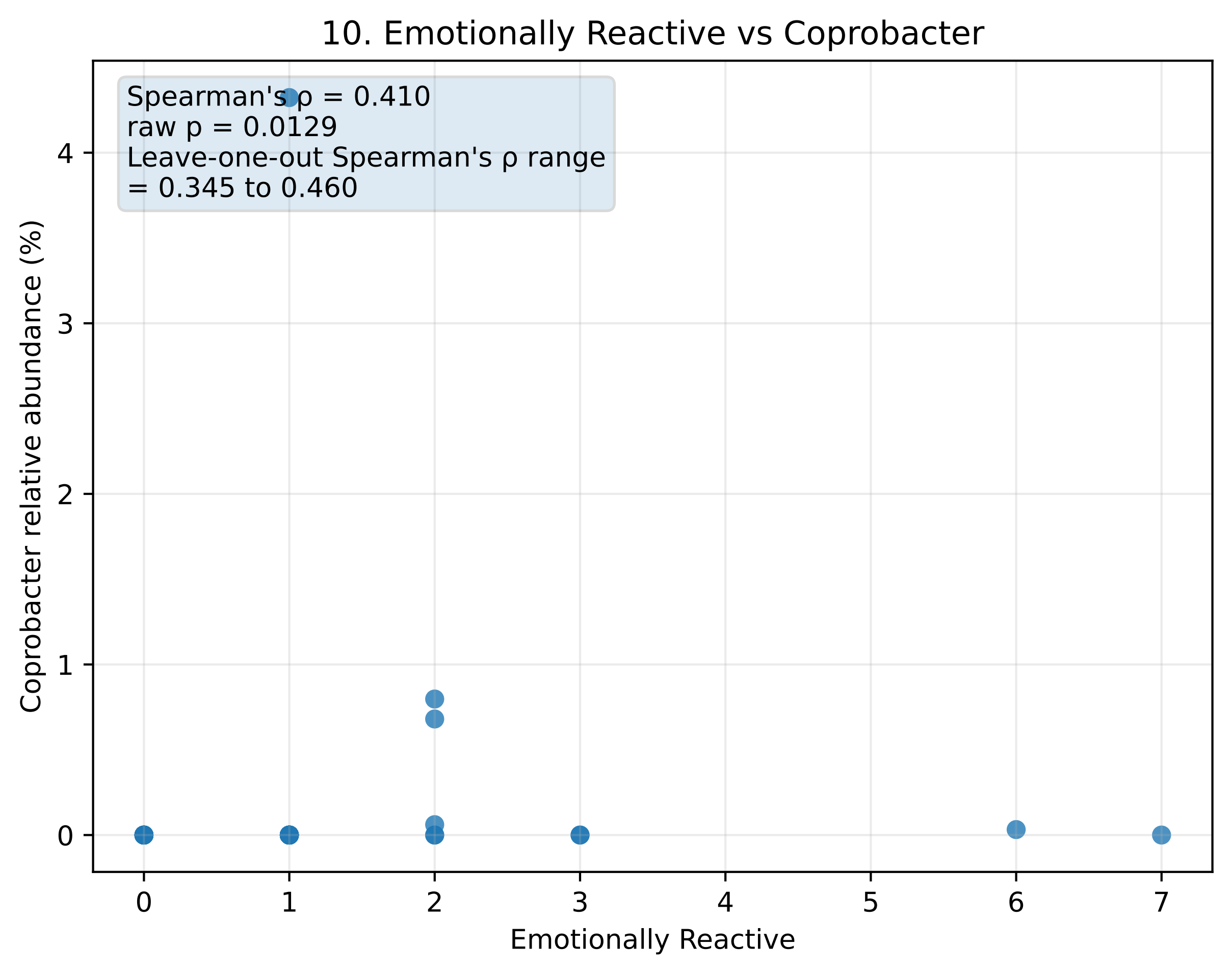
10. Emotionally Reactive vs Coprobacter
Spearman's ρ = 0.410
raw p = 0.0129
Leave-one-out Spearman's ρ range
= 0.345 to 0.460
Coprobacter relative abundance (%)
Emotionally Reactive
0
1
2
3
4
5
6
7
0
1
2
3
4

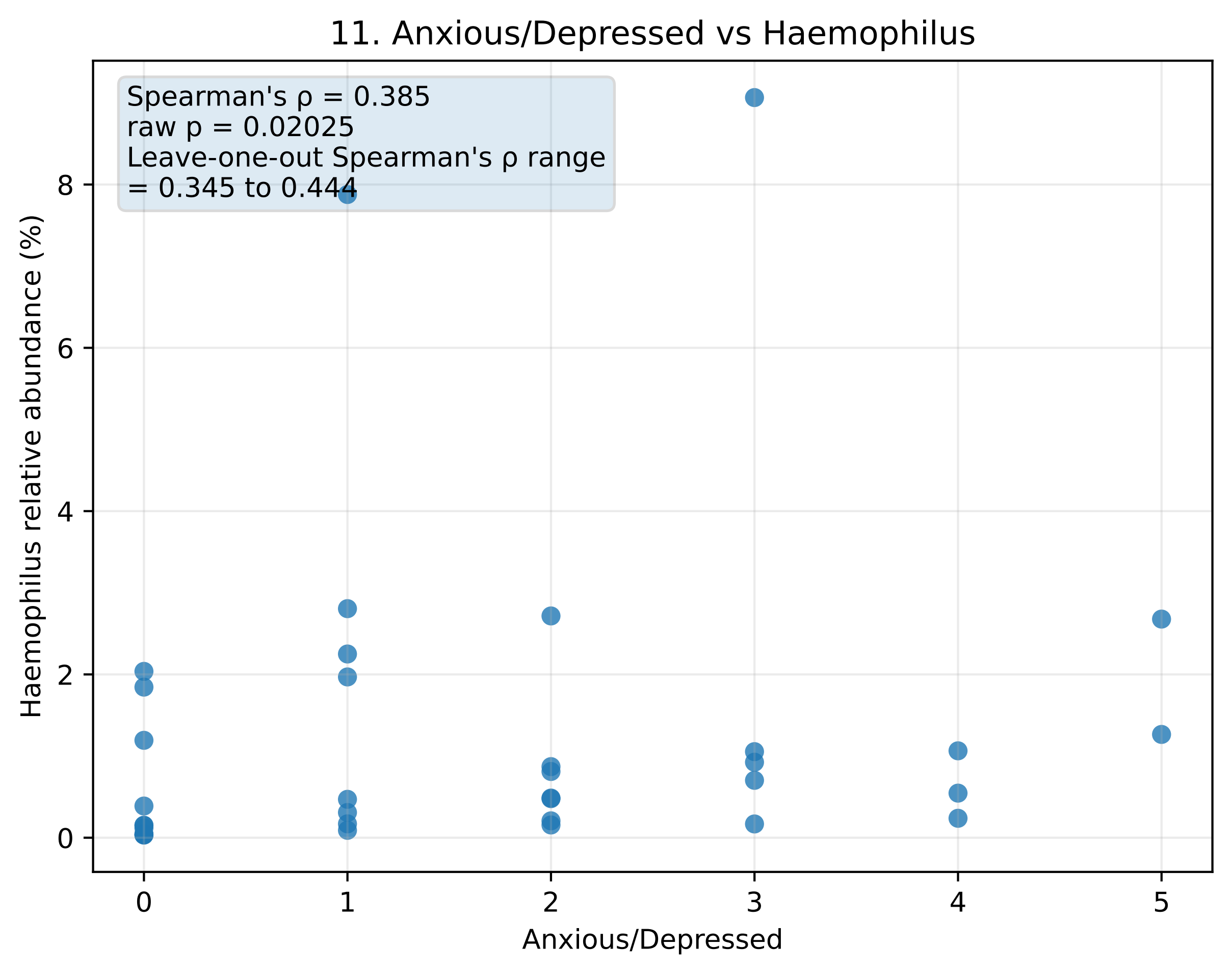
11. Anxious/Depressed vs Haemophilus
Spearman's ρ = 0.385
raw p = 0.02025
Leave-one-out Spearman's ρ range
= 0.345 to 0.444
Haemophilus relative abundance (%)
Anxious/Depressed
0
2
4
6
8
0
1
2
3
4
5

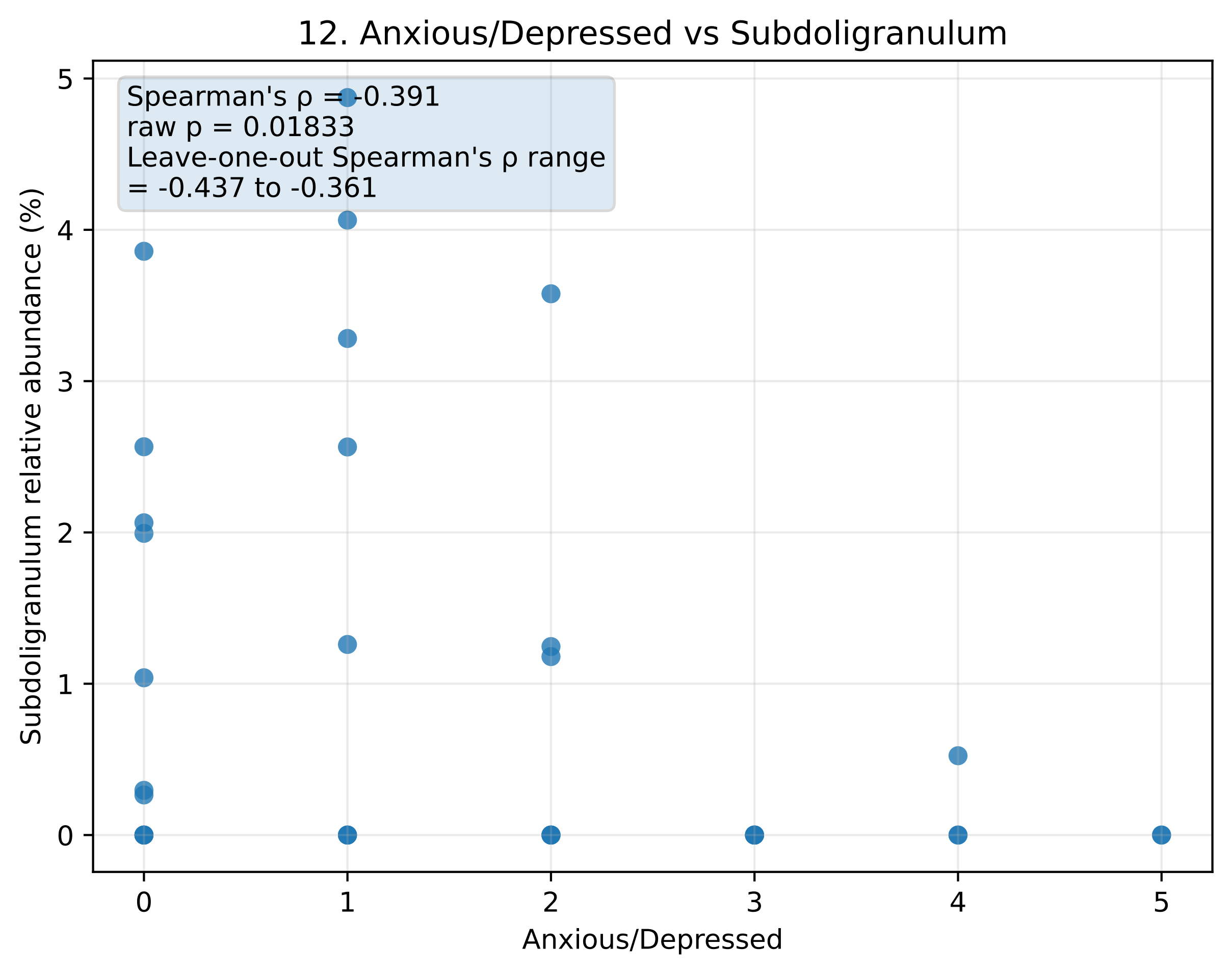
12. Anxious/Depressed vs Subdoligranulum
Spearman's ρ = -0.391
raw p = 0.01833
Leave-one-out Spearman's ρ range
= -0.437 to -0.361
Subdoligranulum relative abundance (%)
Anxious/Depressed
0
1
2
3
4
5

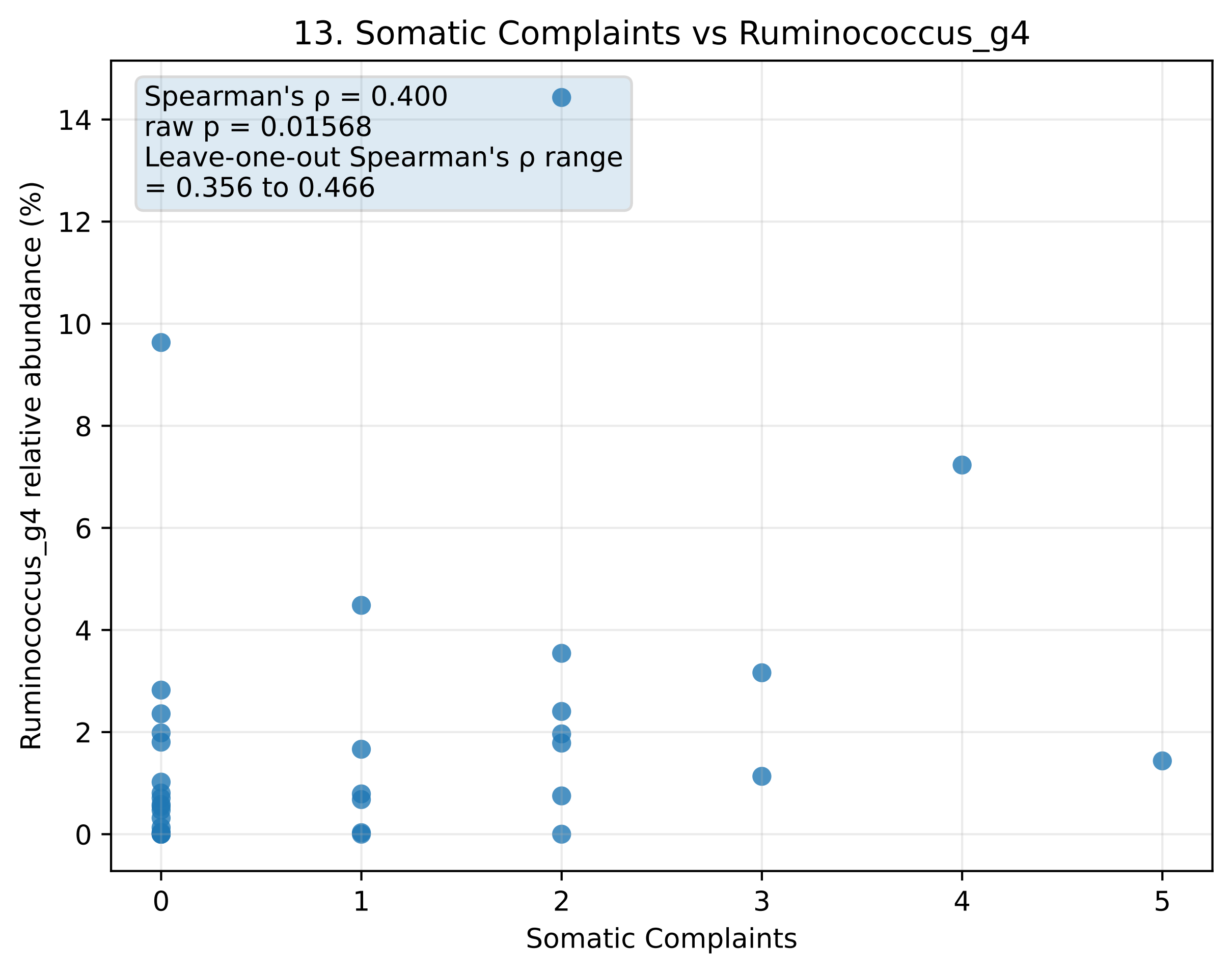
13. Somatic Complaints vs Ruminococcus_g4
Spearman's ρ = 0.400
raw p = 0.01568
Leave-one-out Spearman's ρ range
= 0.356 to 0.466
Ruminococcus_g4 relative abundance (%)
Somatic Complaints
0
2
4
6
8
10
12
14
0
1
2
3
4
5

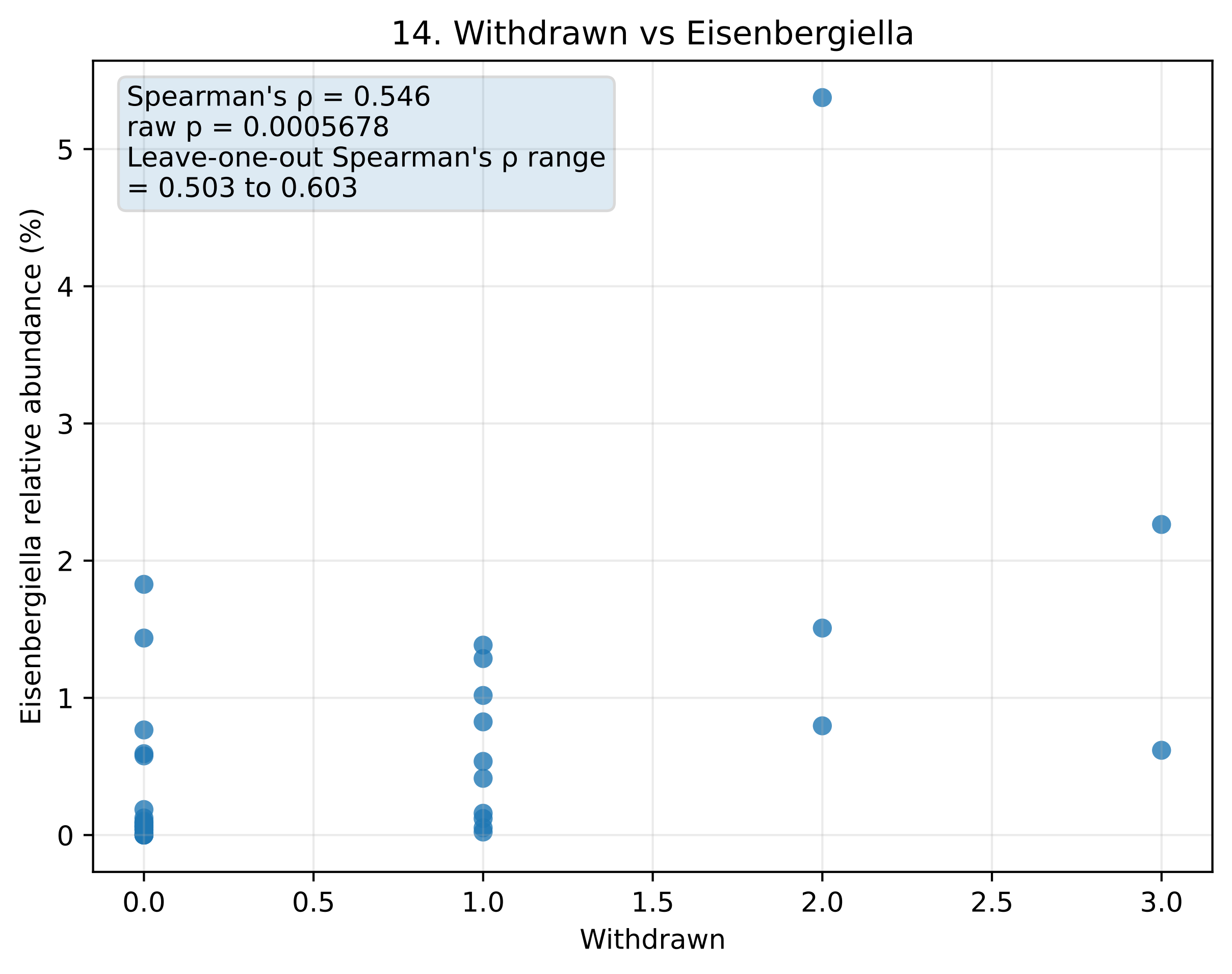
14. Withdrawn vs Eisenbergiella
Spearman's ρ = 0.546
raw p = 0.0005678
Leave-one-out Spearman's ρ range
= 0.503 to 0.603
Eisenbergiella relative abundance (%)
Withdrawn
0
1
2
3
4
5
0.0
0.5
1.0
1.5
2.0
2.5
3.0

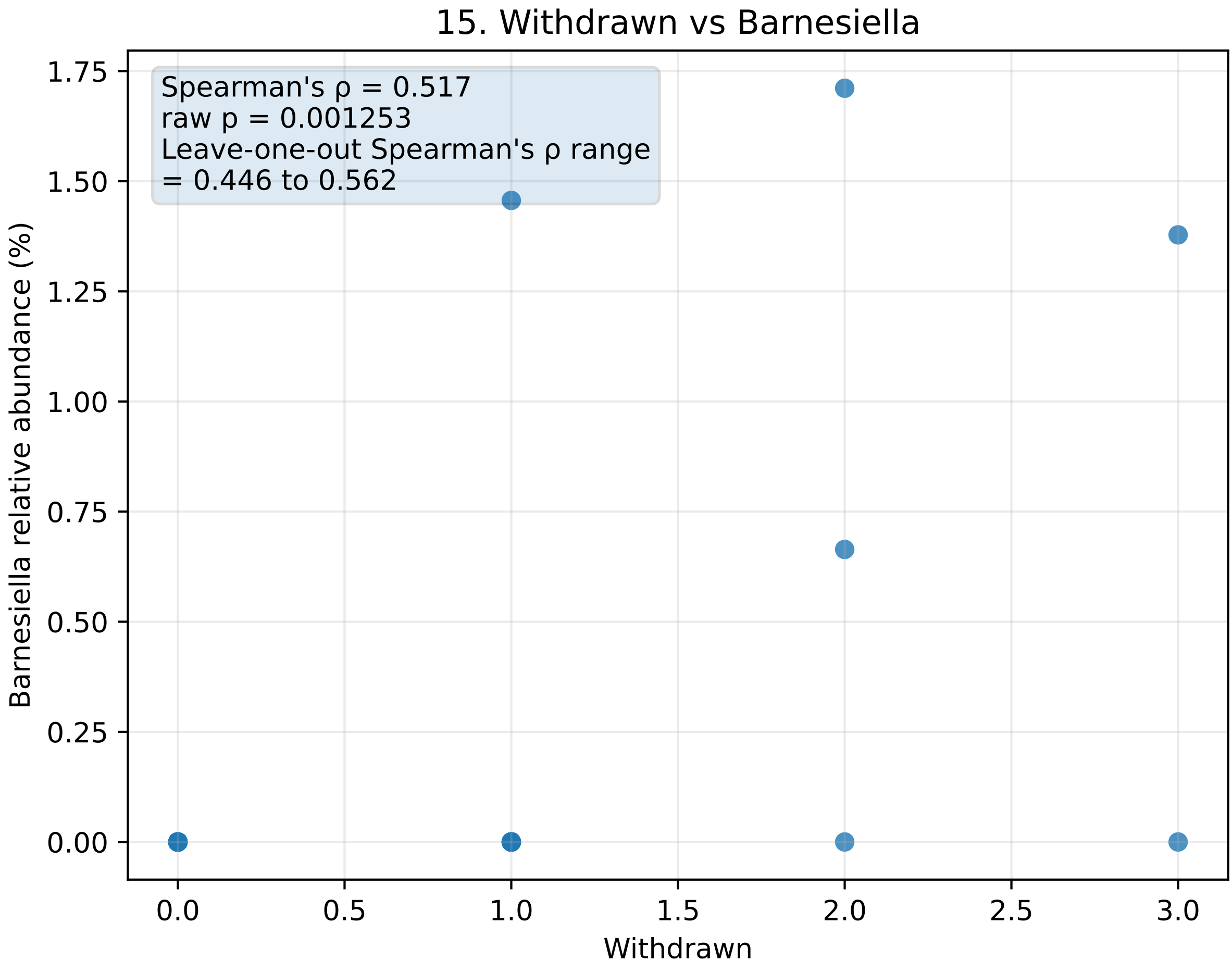
15. Withdrawn vs Barnesiella
Spearman's ρ = 0.517
raw p = 0.001253
Leave-one-out Spearman's ρ range
= 0.446 to 0.562
Barnesiella relative abundance (%)
1.75
1.50
1.25
1.00
0.75
0.50
0.25
0.00
0.0
0.5
1.0
1.5
2.0
2.5
3.0
Withdrawn

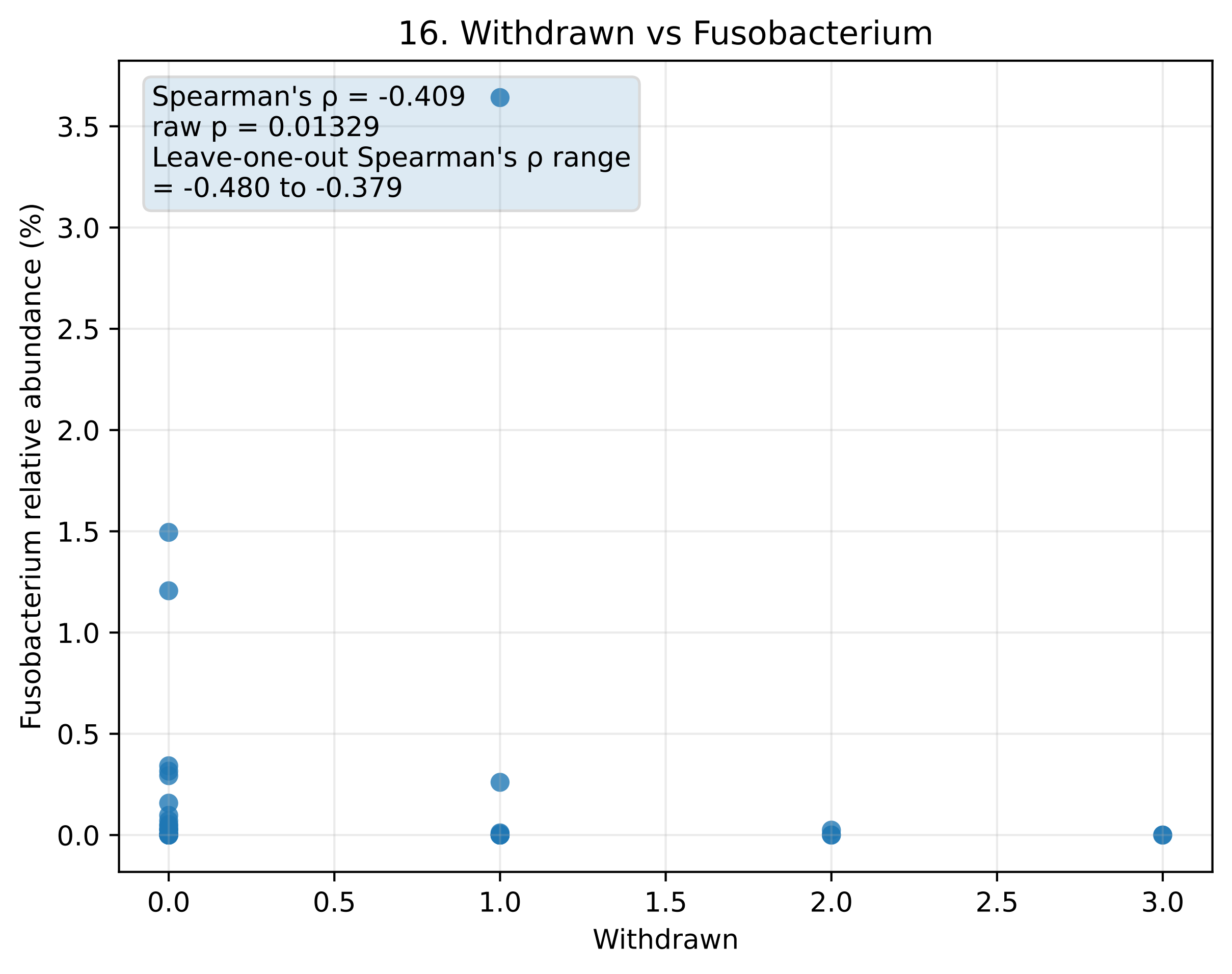
16. Withdrawn vs Fusobacterium
Spearman's ρ = -0.409
raw p = 0.01329
Leave-one-out Spearman's ρ range
= -0.480 to -0.379
Fusobacterium relative abundance (%)
Withdrawn
0.0
0.5
1.0
1.5
2.0
2.5
3.0
3.5

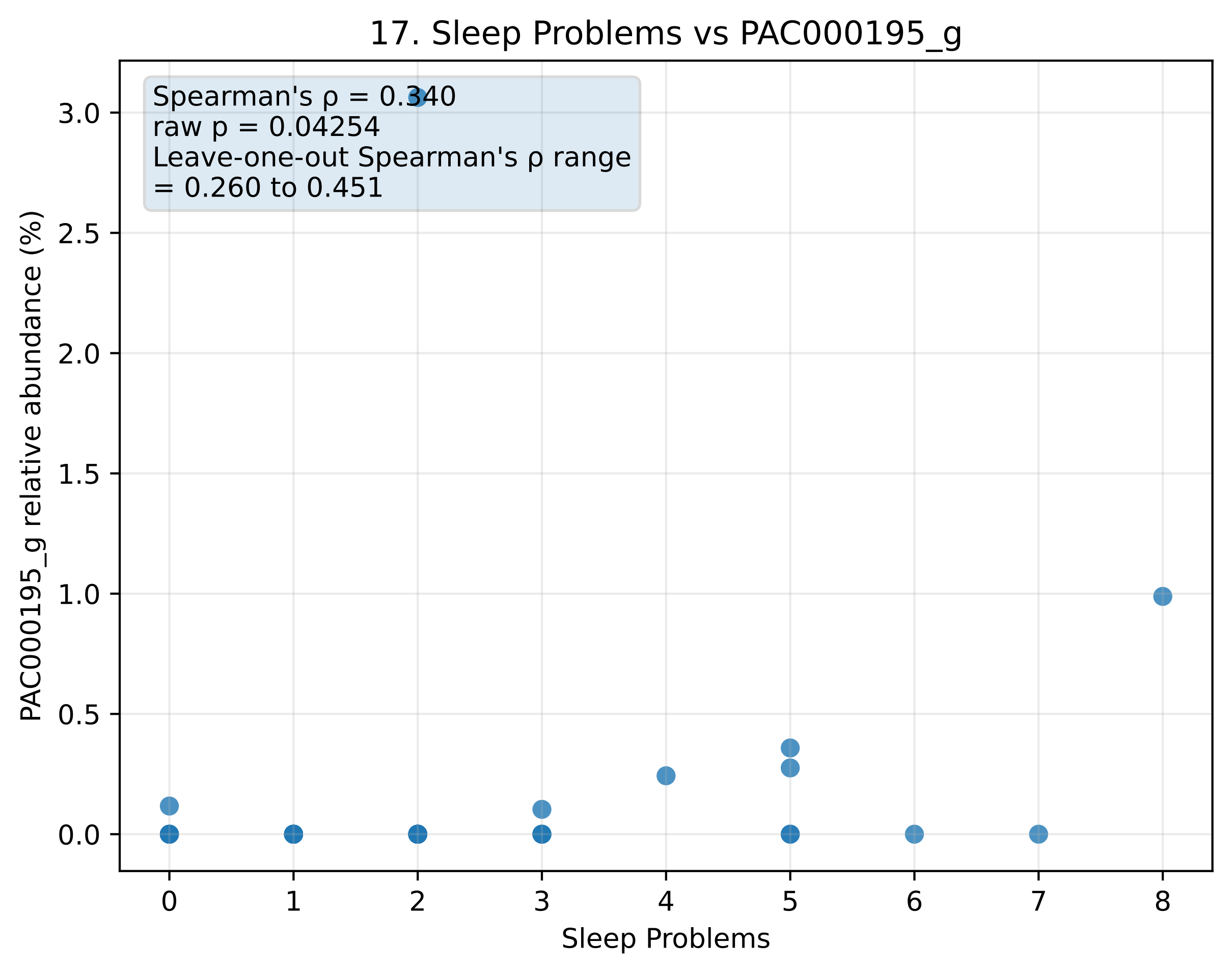
17. Sleep Problems vs PAC000195_g
Spearman's ρ = 0.340
raw p = 0.04254
Leave-one-out Spearman's ρ range
= 0.260 to 0.451
PAC000195_g relative abundance (%)
Sleep Problems
3.0
2.5
2.0
1.5
1.0
0.5
0.0
0
1
2
3
4
5
6
7
8

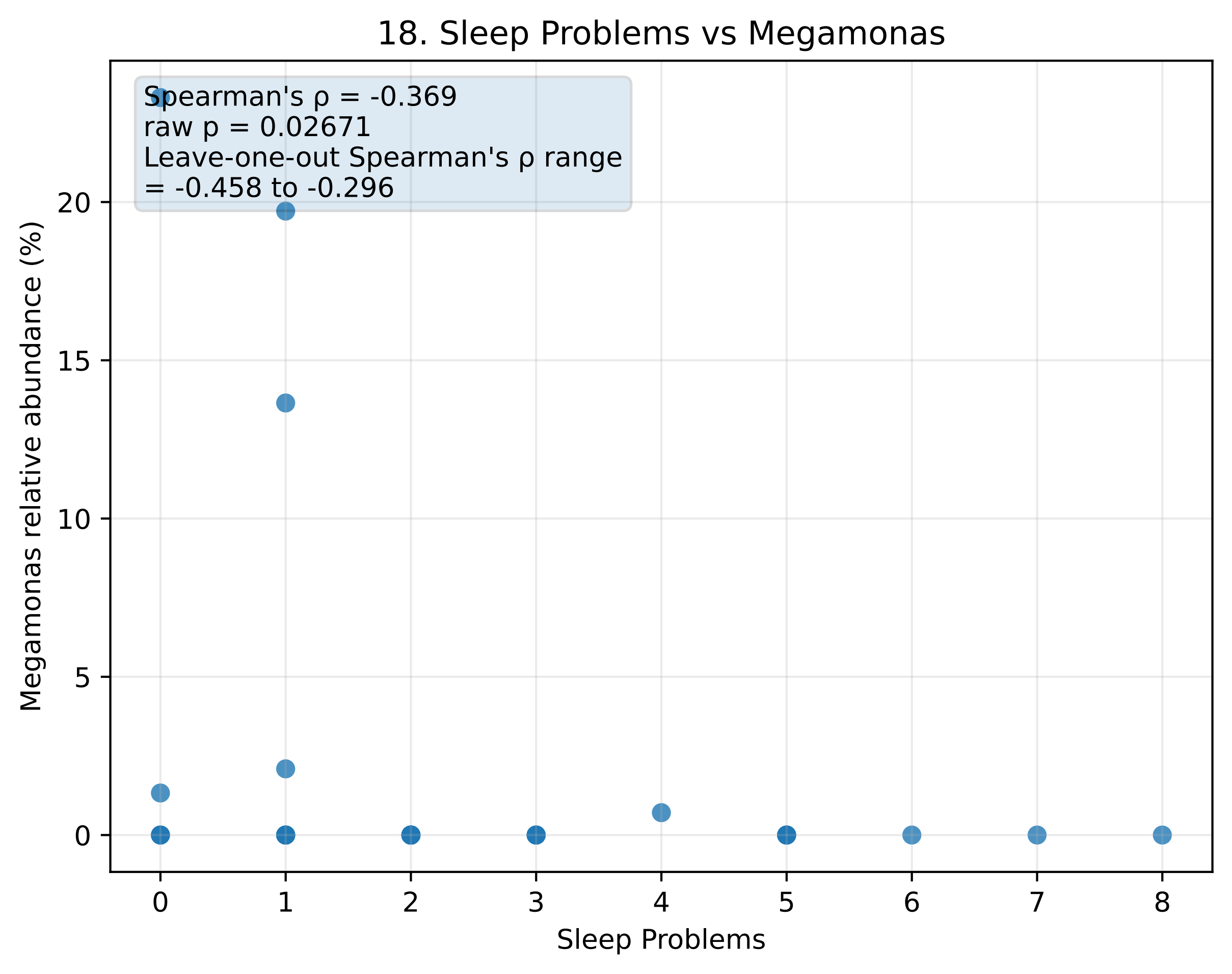
18. Sleep Problems vs Megamonas
Spearman's ρ = -0.369
raw p = 0.02671
Leave-one-out Spearman's ρ range
= -0.458 to -0.296
Megamonas relative abundance (%)
0
5
10
15
20
Sleep Problems
0
1
2
3
4
5
6
7
8

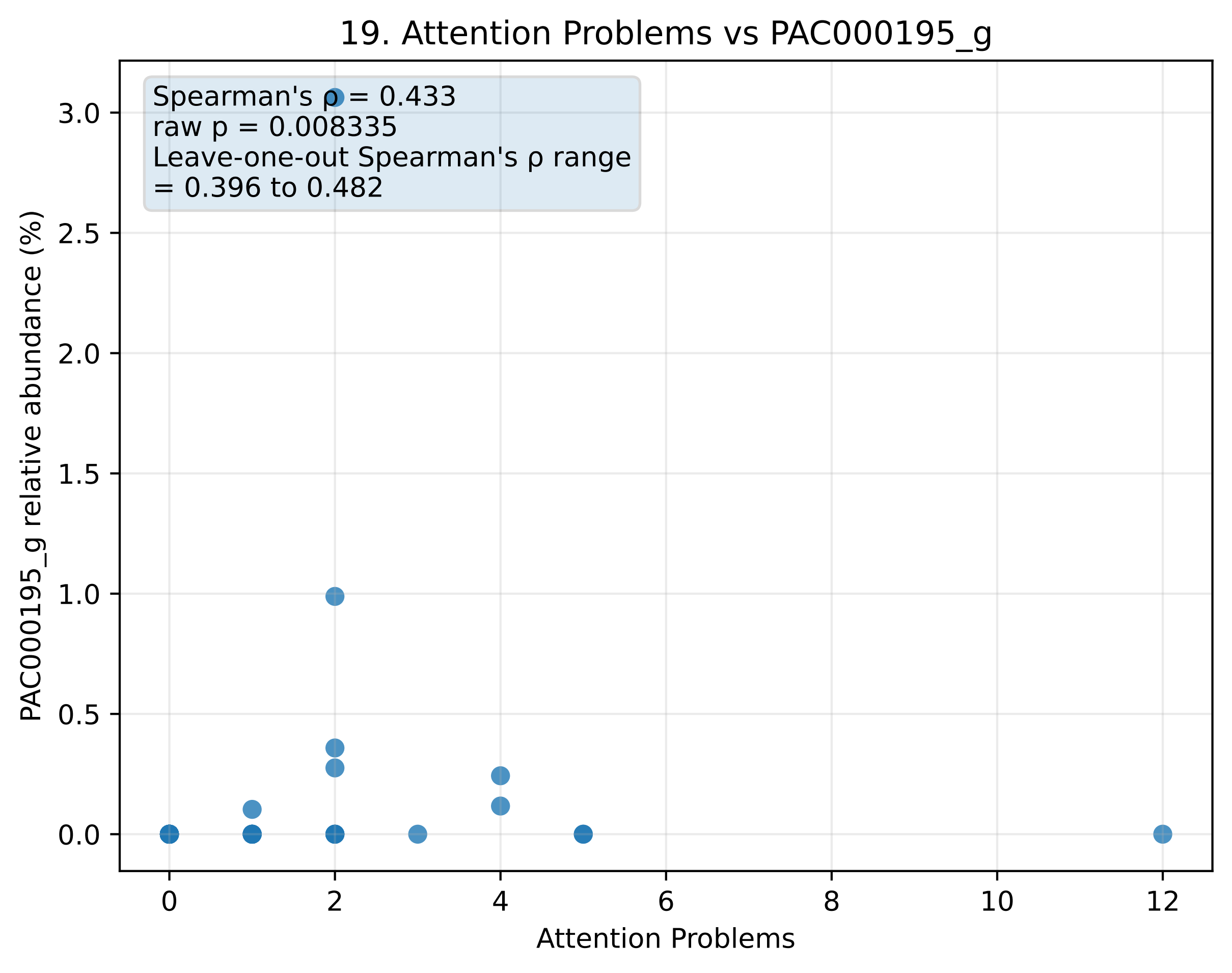
19. Attention Problems vs PAC000195_g
Spearman's ρ = 0.433
raw p = 0.008335
Leave-one-out Spearman's ρ range
= 0.396 to 0.482
PAC000195_g relative abundance (%)
3.0
2.5
2.0
1.5
1.0
0.5
0.0
0
2
4
6
8
10
12
Attention Problems

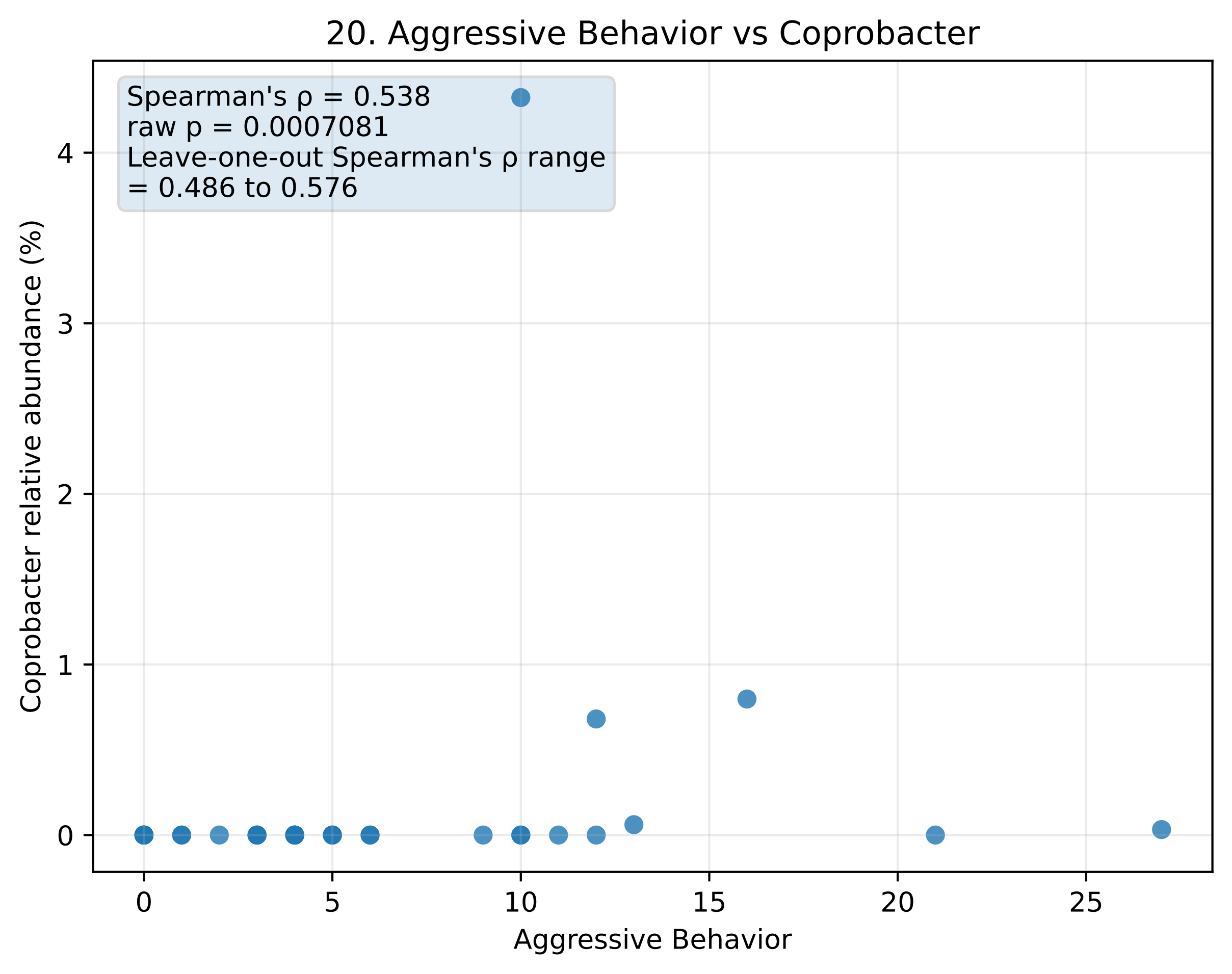
20. Aggressive Behavior vs Coprobacter
Spearman's ρ = 0.538
raw p = 0.0007081
Leave-one-out Spearman's ρ range
= 0.486 to 0.576
Coprobacter relative abundance (%)
Aggressive Behavior
0
1
2
3
4
0
5
10
15
20
25

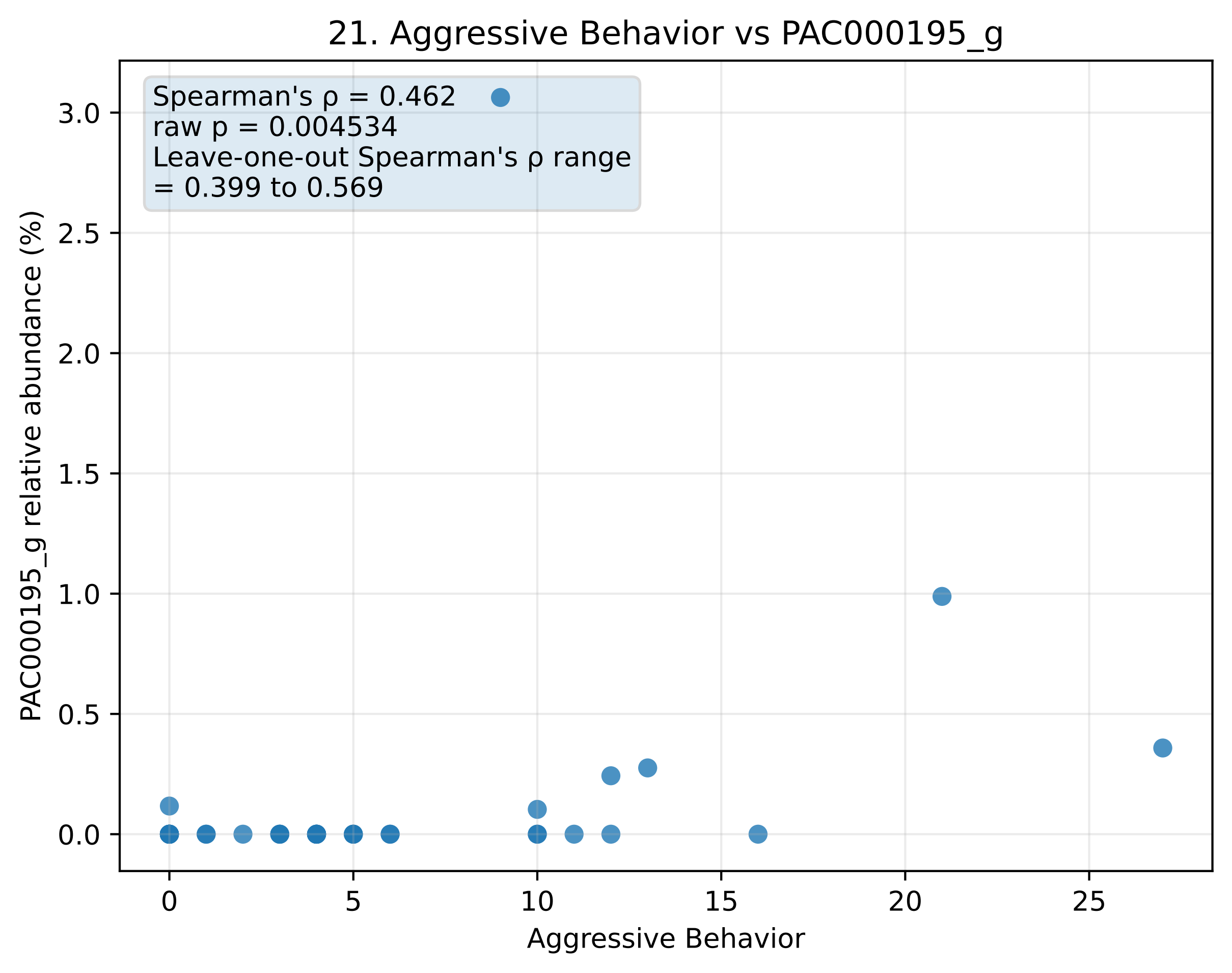
21. Aggressive Behavior vs PAC000195_g
Spearman's ρ = 0.462
raw p = 0.004534
Leave-one-out Spearman's ρ range
= 0.399 to 0.569
PAC000195_g relative abundance (%)
3.0
2.5
2.0
1.5
1.0
0.5
0.0
0
5
10
15
20
25
Aggressive Behavior
