# Supplementary material for: Gut microbiome composition and predicted functions relate to growth and behavior in a Japanese preschool cohort
Source: Sci Rep. 2026 Jun 26;16:28604. doi: 10.1038/s41598-026-59018-4 (PMC13575191; doi:10.1038/s41598-026-59018-4)
Supplement: Supplementary file 2 [file 41598_2026_59018_MOESM2_ESM.pdf]

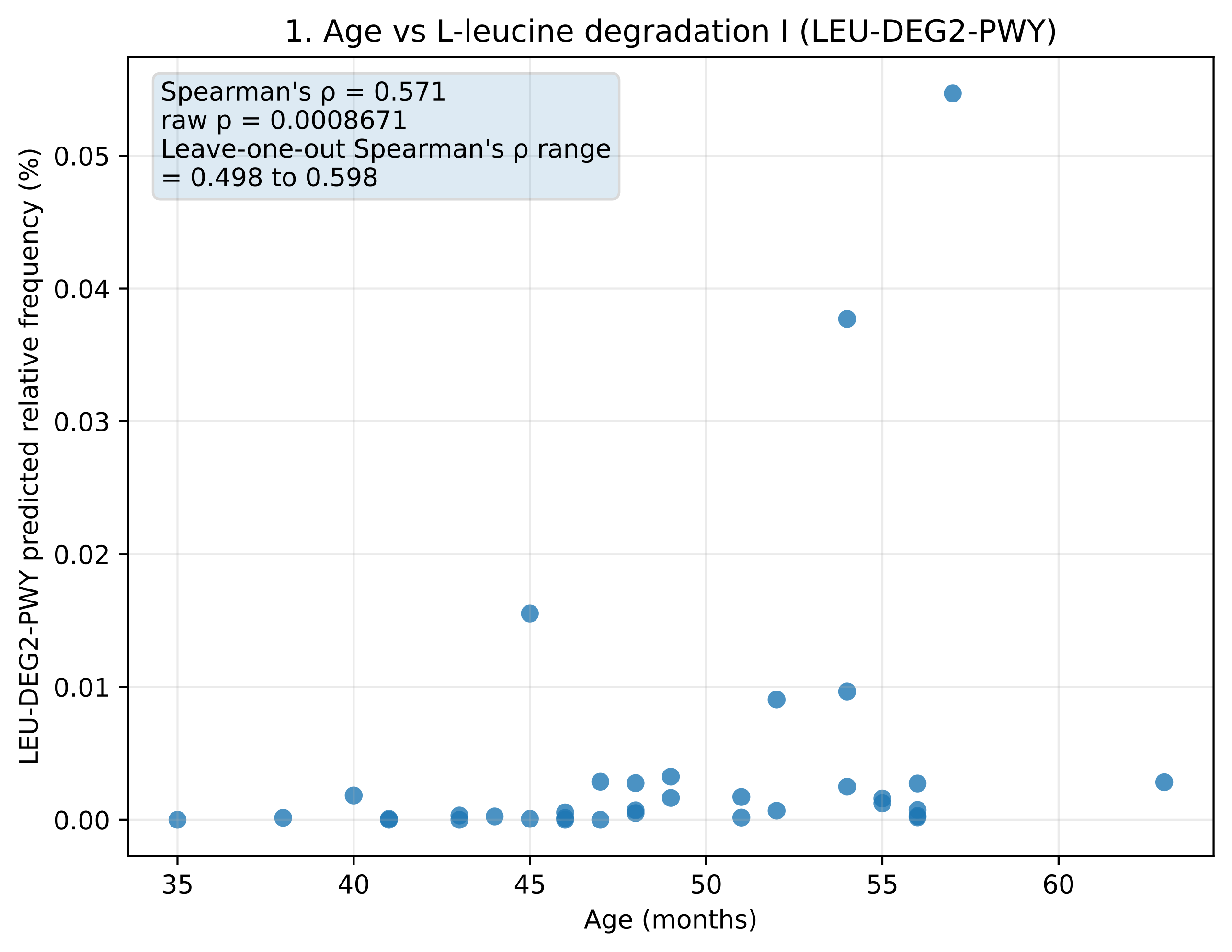
1. Age vs L-leucine degradation I (LEU-DEG2-PWY)
Spearman's ρ = 0.571
raw p = 0.0008671
Leave-one-out Spearman's ρ range
= 0.498 to 0.598
LEU-DEG2-PWY predicted relative frequency (%)
0.00
0.01
0.02
0.03
0.04
0.05
35
40
45
50
55
60
Age (months)

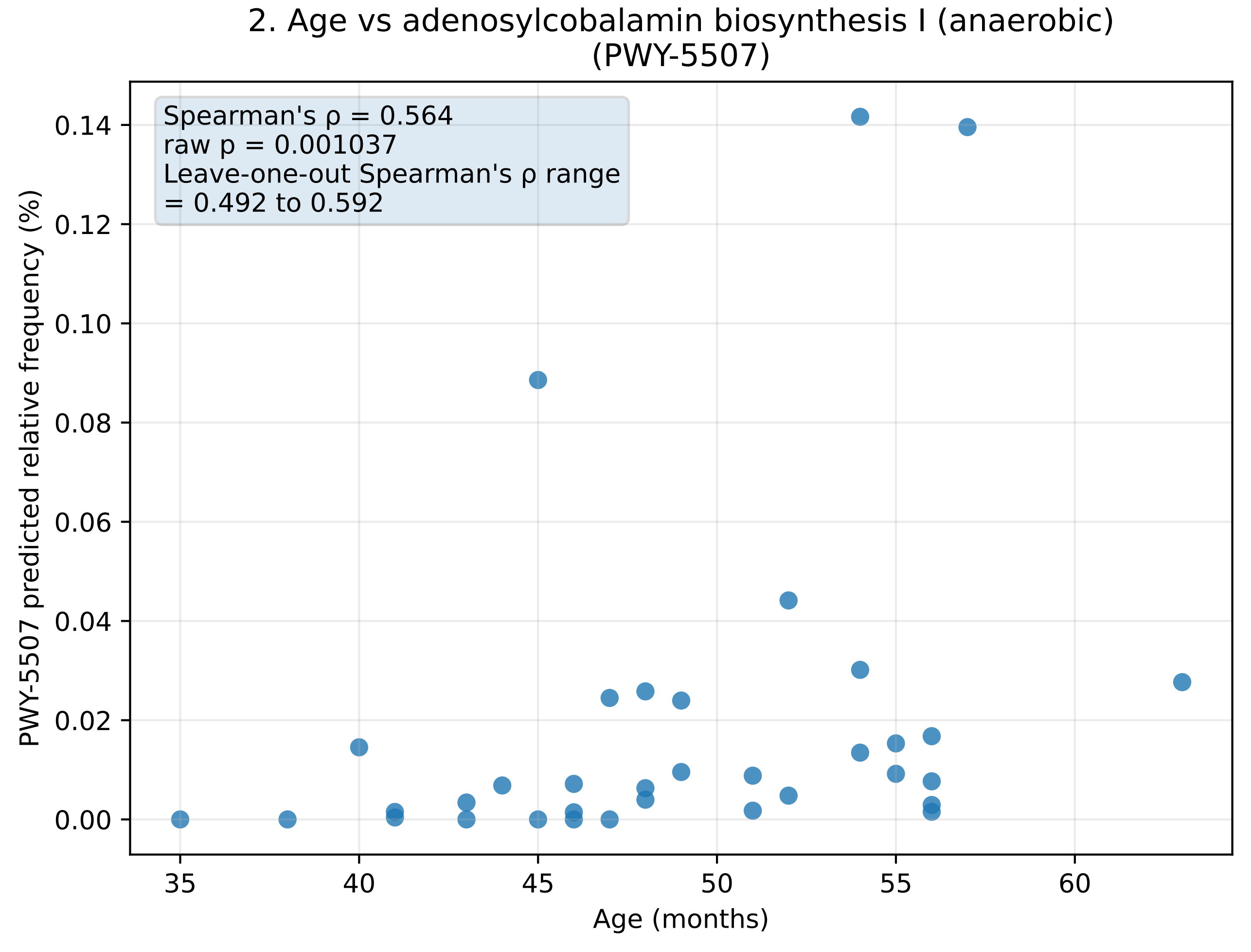
2. Age vs adenosylcobalamin biosynthesis I (anaerobic)
(PWY-5507)
Spearman's ρ = 0.564
raw p = 0.001037
Leave-one-out Spearman's ρ range
= 0.492 to 0.592
PWY-5507 predicted relative frequency (%)
0.00
0.02
0.04
0.06
0.08
0.10
0.12
0.14
35
40
45
50
55
60
Age (months)

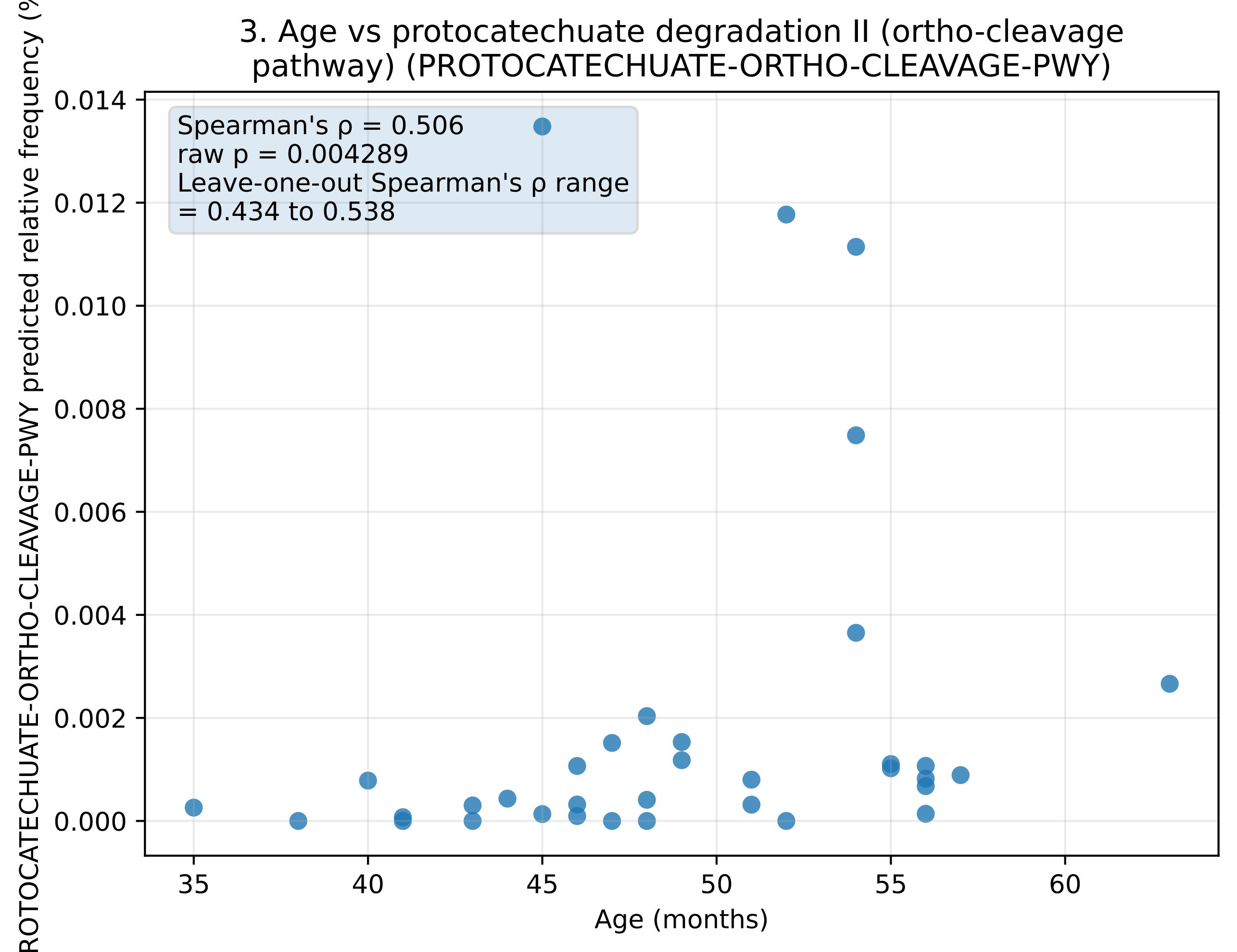

3. Age vs protocatechuate degradation II (ortho-cleavage pathway) (PROTOCATECHUATE-ORTHO-CLEAVAGE-PWY)
Spearman's ρ = 0.506
raw p = 0.004289
Leave-one-out Spearman's ρ range = 0.434 to 0.538
ROTOCATECHUATE-ORTHO-CLEAVAGE-PWY predicted relative frequency (%
Age (months)
0.014
0.012
0.010
0.008
0.006
0.004
0.002
0.000
35
40
45
50
55
60

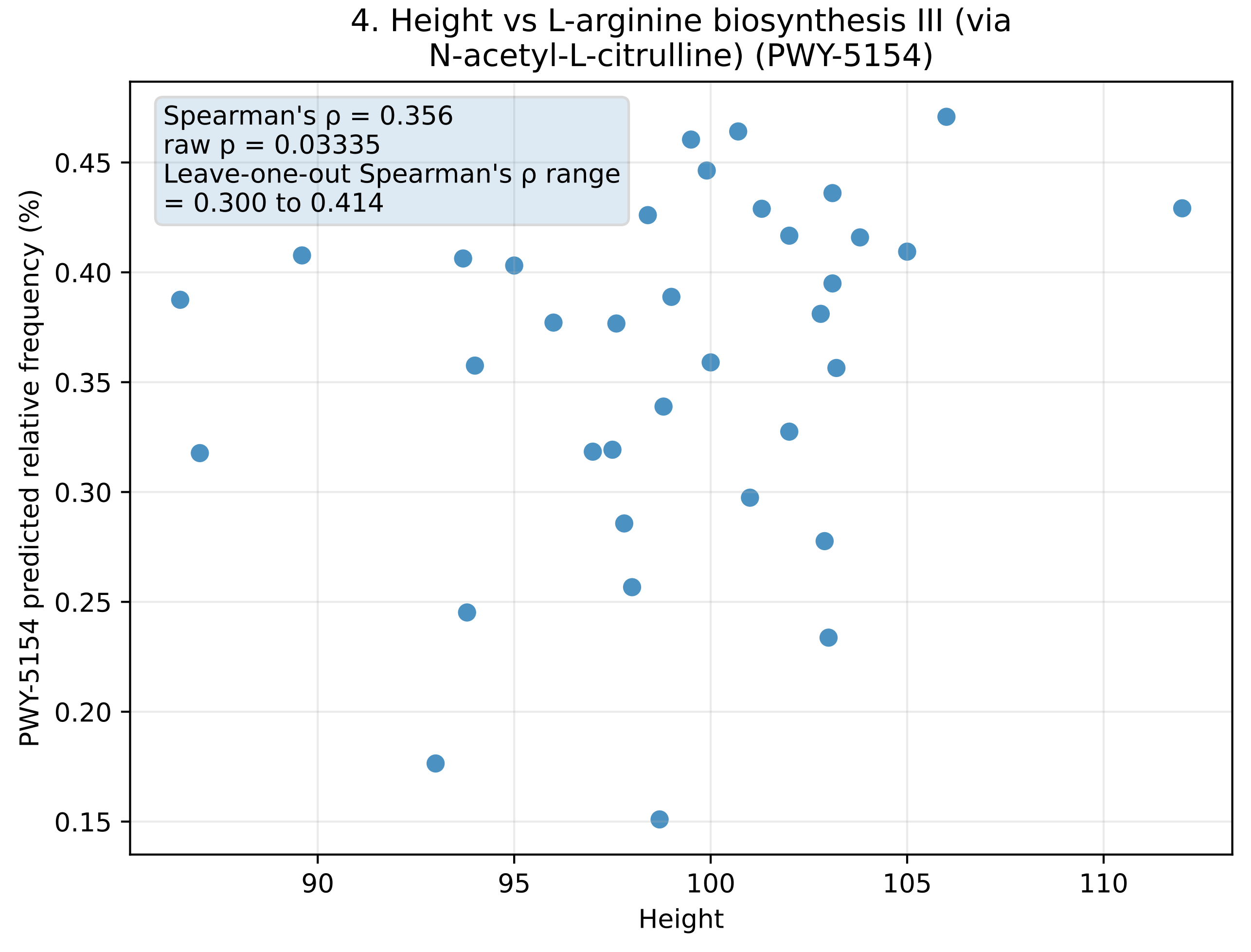
4. Height vs L-arginine biosynthesis III (via N-acetyl-L-citrulline) (PWY-5154)
Spearman's ρ = 0.356
raw p = 0.03335
Leave-one-out Spearman's ρ range = 0.300 to 0.414
PWY-5154 predicted relative frequency (%)
0.15
0.20
0.25
0.30
0.35
0.40
0.45
90
95
100
105
110
Height

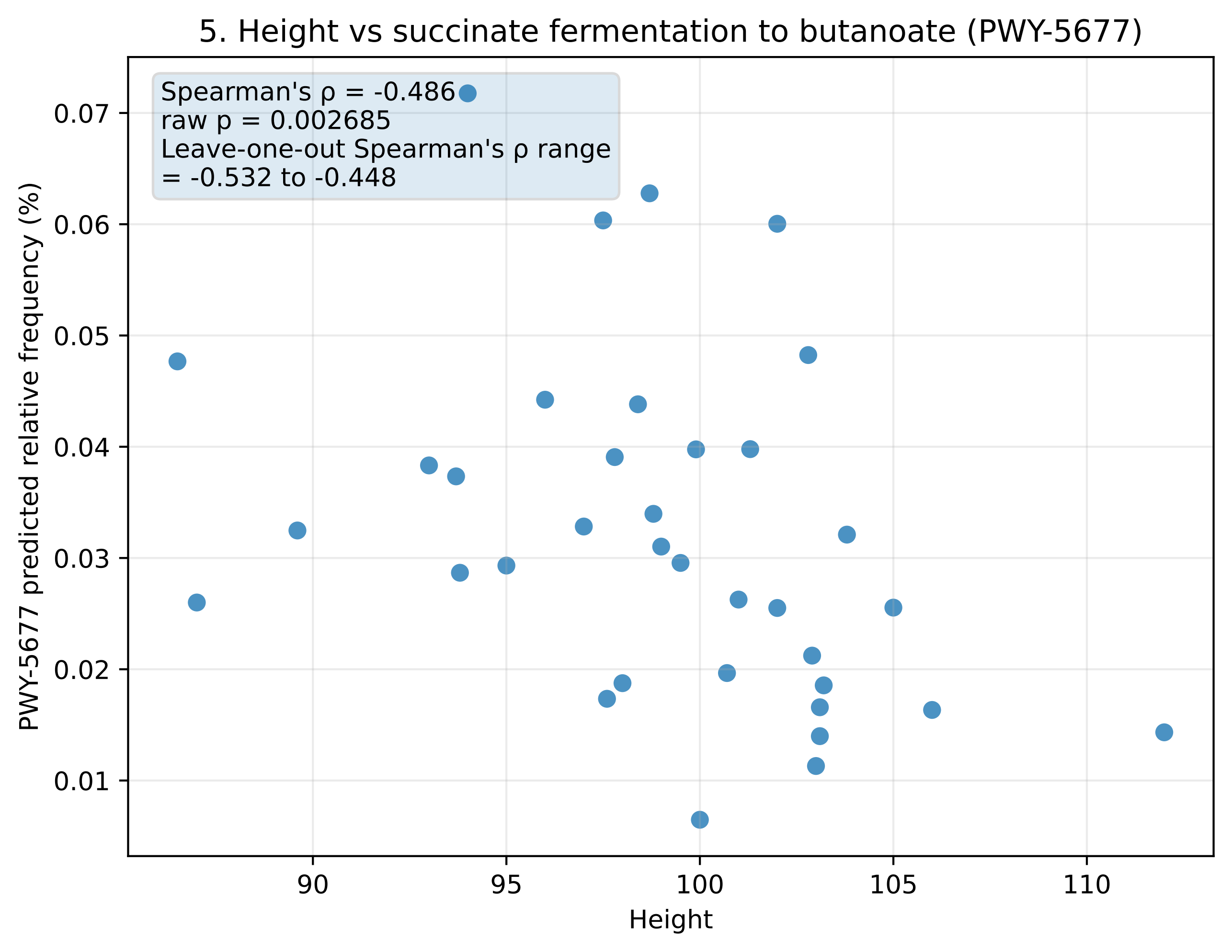
5. Height vs succinate fermentation to butanoate (PWY-5677)
Spearman's ρ = -0.486
raw p = 0.002685
Leave-one-out Spearman's ρ range
= -0.532 to -0.448
0.07
0.06
0.05
0.04
0.03
0.02
0.01
PWY-5677 predicted relative frequency (%)
90
95
100
105
110
Height

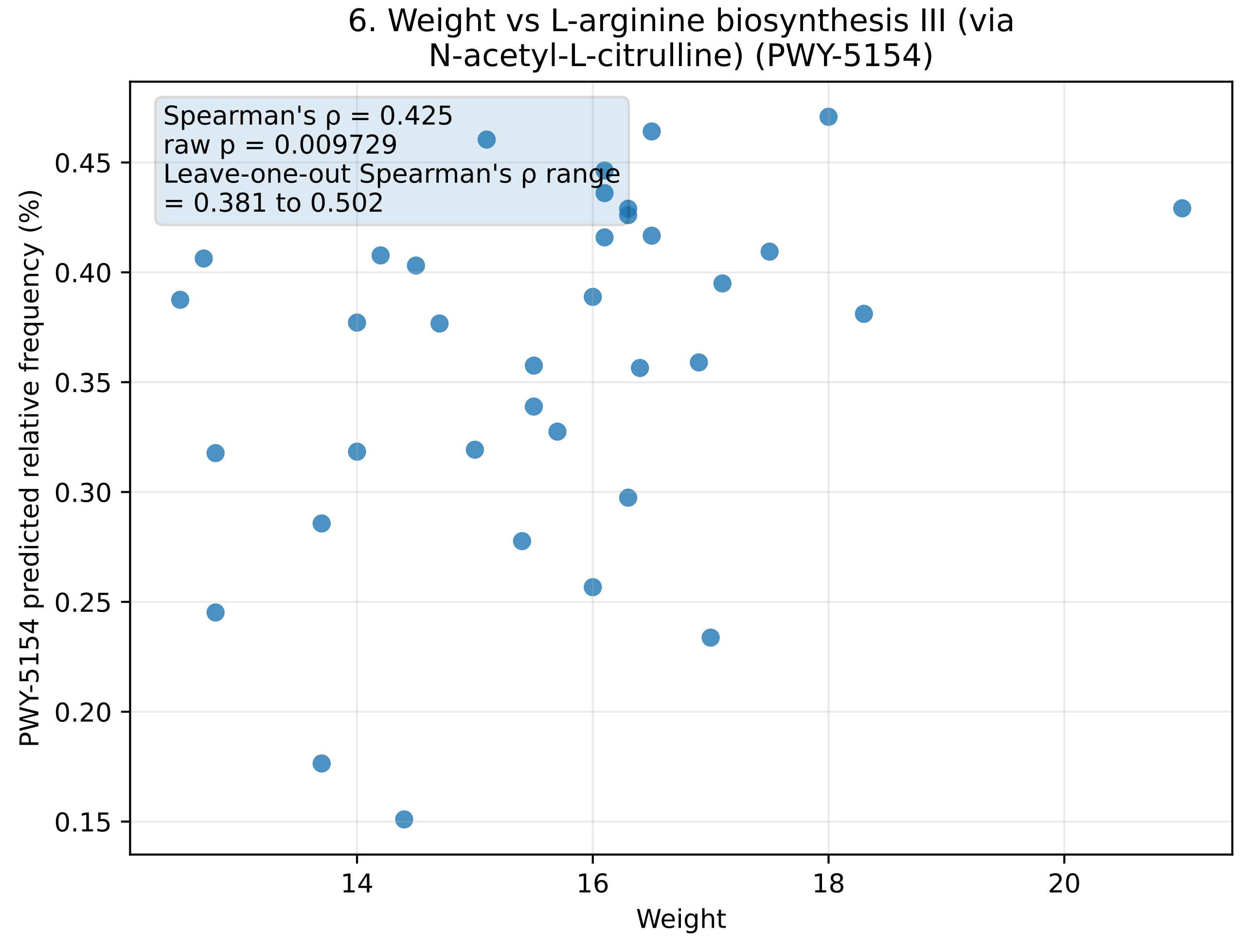
6. Weight vs L-arginine biosynthesis III (via N-acetyl-L-citrulline) (PWY-5154)
Spearman's ρ = 0.425
raw p = 0.009729
Leave-one-out Spearman's ρ range = 0.381 to 0.502
PWY-5154 predicted relative frequency (%)
0.45
0.40
0.35
0.30
0.25
0.20
0.15
14
16
18
20
Weight

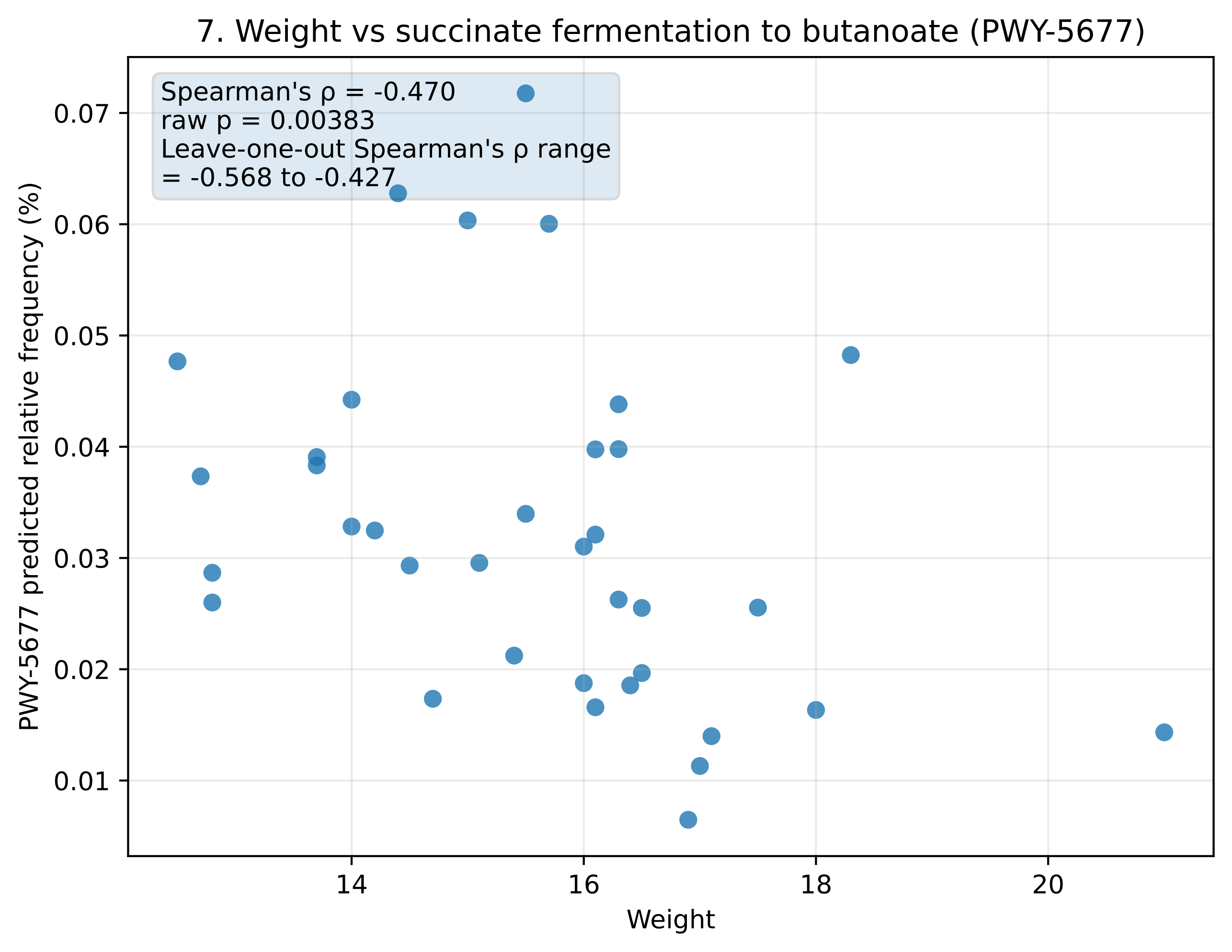
7. Weight vs succinate fermentation to butanoate (PWY-5677)
Spearman's ρ = -0.470
raw p = 0.00383
Leave-one-out Spearman's ρ range
= -0.568 to -0.427
PWY-5677 predicted relative frequency (%)
Weight
0.01
0.02
0.03
0.04
0.05
0.06
0.07
14
16
18
20

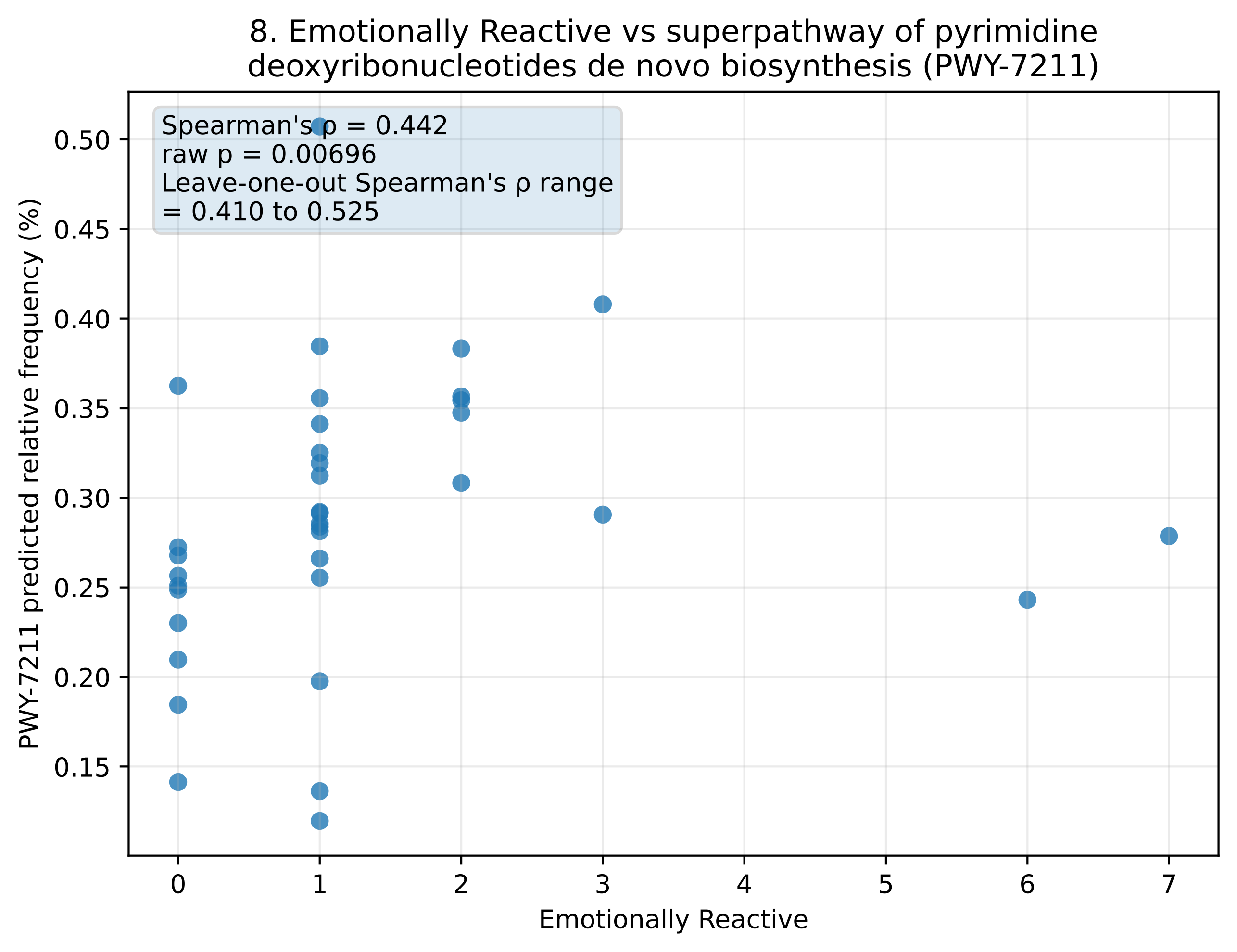
8. Emotionally Reactive vs superpathway of pyrimidine deoxyribonucleotides de novo biosynthesis (PWY-7211)
Spearman's ρ = 0.442
raw p = 0.00696
Leave-one-out Spearman's ρ range = 0.410 to 0.525
PWY-7211 predicted relative frequency (%)
0.50
0.45
0.40
0.35
0.30
0.25
0.20
0.15
0
1
2
3
4
5
6
7
Emotionally Reactive

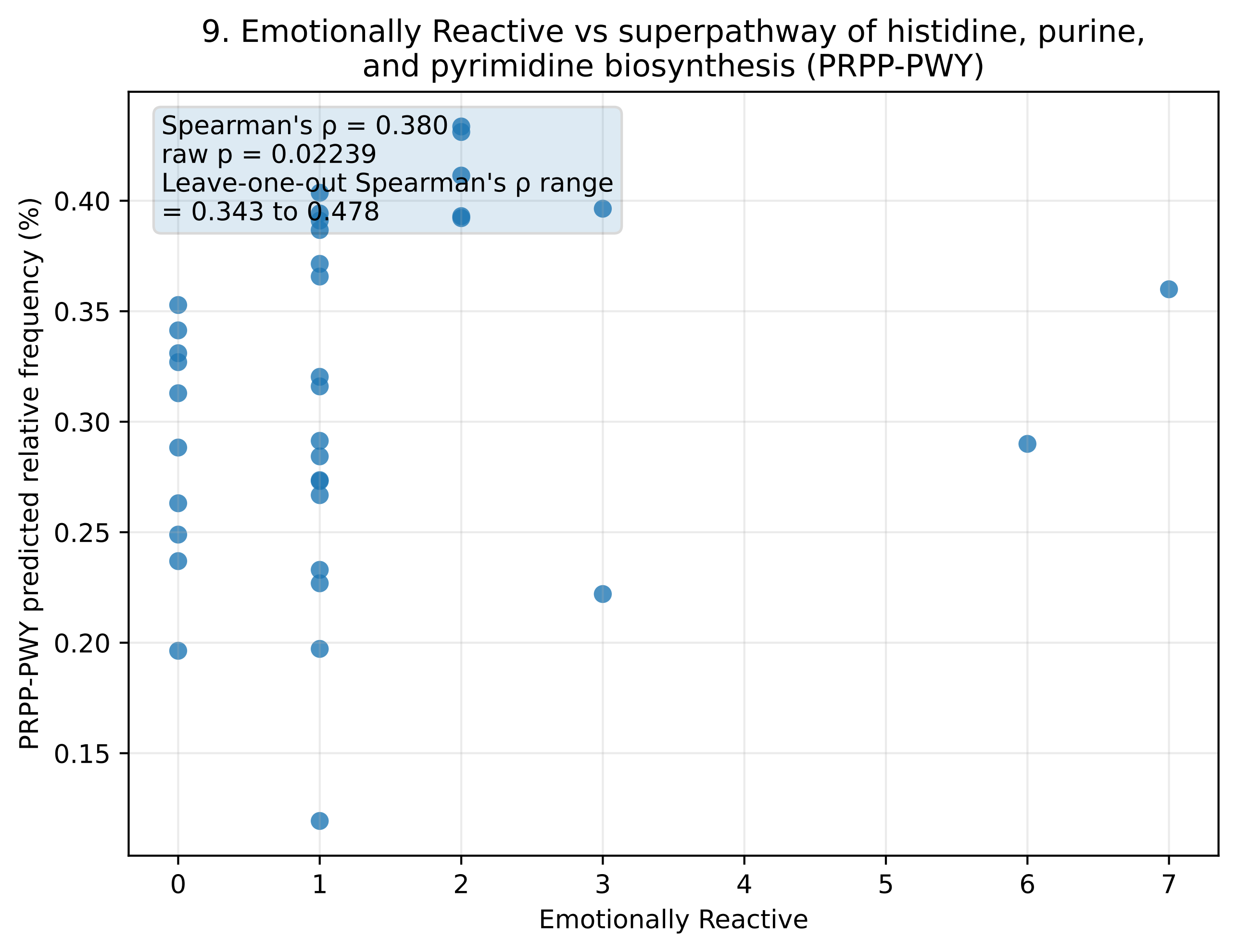
9. Emotionally Reactive vs superpathway of histidine, purine, and pyrimidine biosynthesis (PRPP-PWY)
Spearman's ρ = 0.380
raw p = 0.02239
Leave-one-out Spearman's ρ range = 0.343 to 0.478
PRPP-PWY predicted relative frequency (%)
0.40
0.35
0.30
0.25
0.20
0.15
0
1
2
3
4
5
6
7
Emotionally Reactive

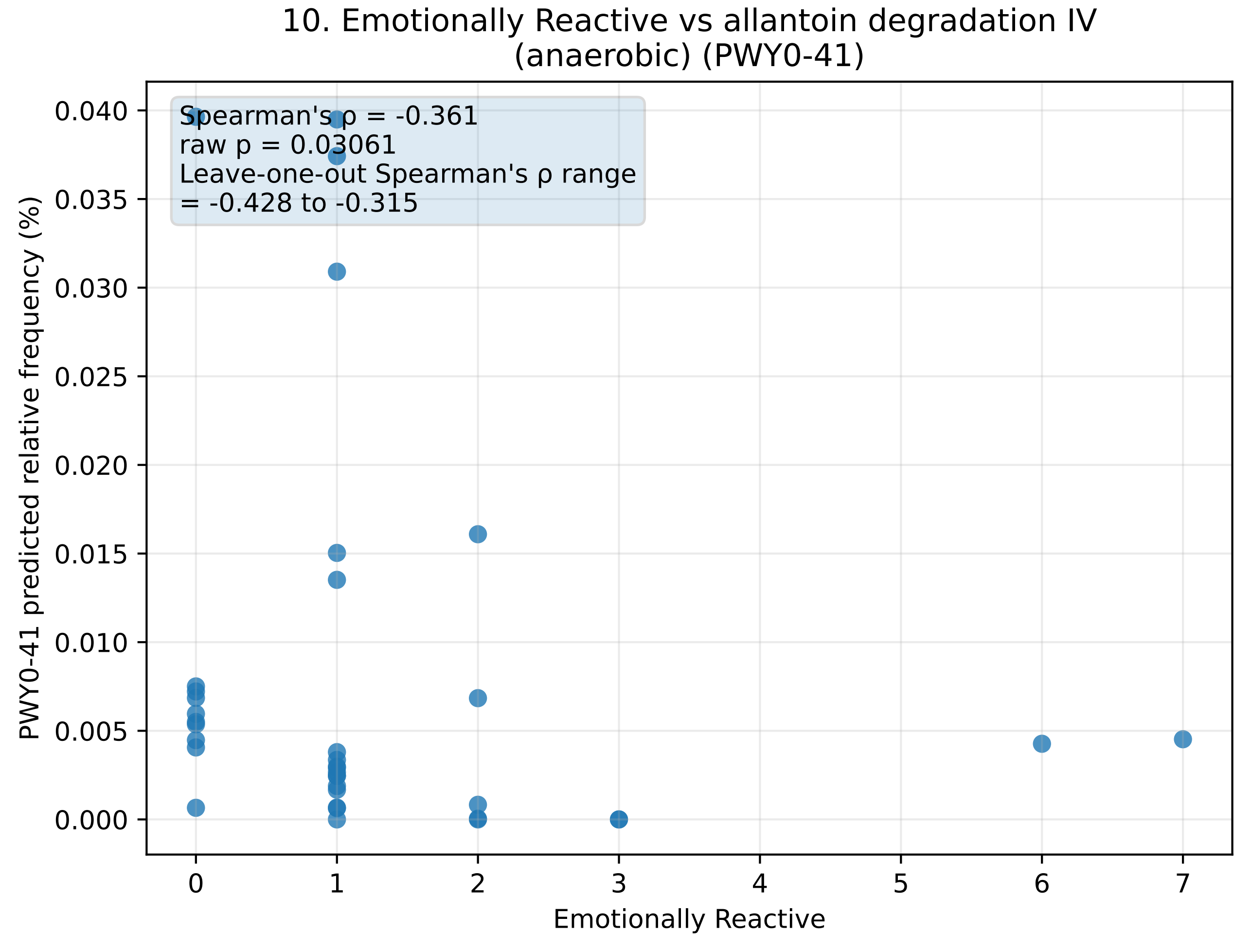
10. Emotionally Reactive vs allantoin degradation IV
(anaerobic) (PWY0-41)
Spearman's ρ = -0.361
raw p = 0.03061
Leave-one-out Spearman's ρ range
= -0.428 to -0.315
PWY0-41 predicted relative frequency (%)
0.040
0.035
0.030
0.025
0.020
0.015
0.010
0.005
0.000
0
1
2
3
4
5
6
7
Emotionally Reactive

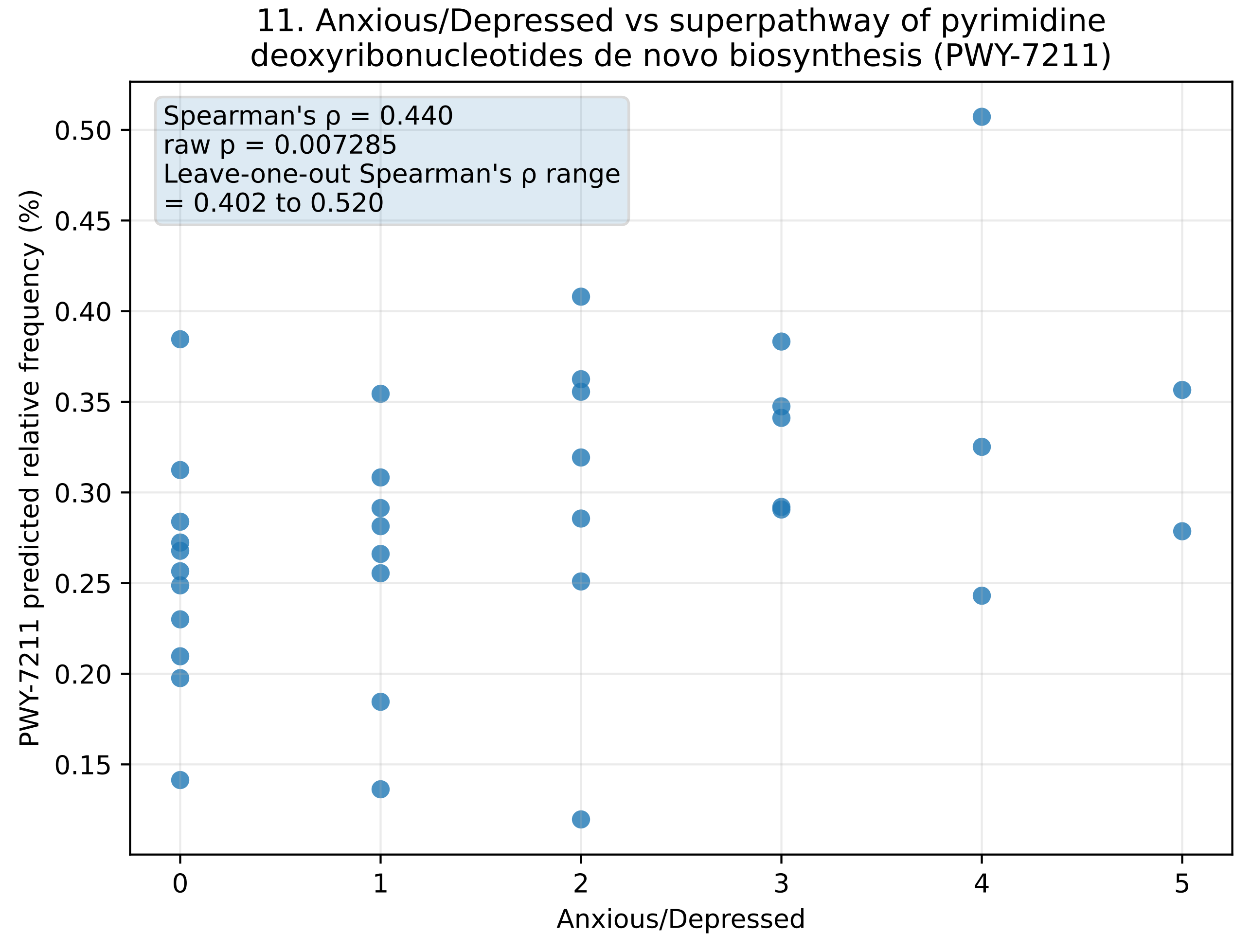
11. Anxious/Depressed vs superpathway of pyrimidine
deoxyribonucleotides de novo biosynthesis (PWY-7211)
Spearman's ρ = 0.440
raw p = 0.007285
Leave-one-out Spearman's ρ range
= 0.402 to 0.520
PWY-7211 predicted relative frequency (%)
0.50
0.45
0.40
0.35
0.30
0.25
0.20
0.15
0
1
2
3
4
5
Anxious/Depressed

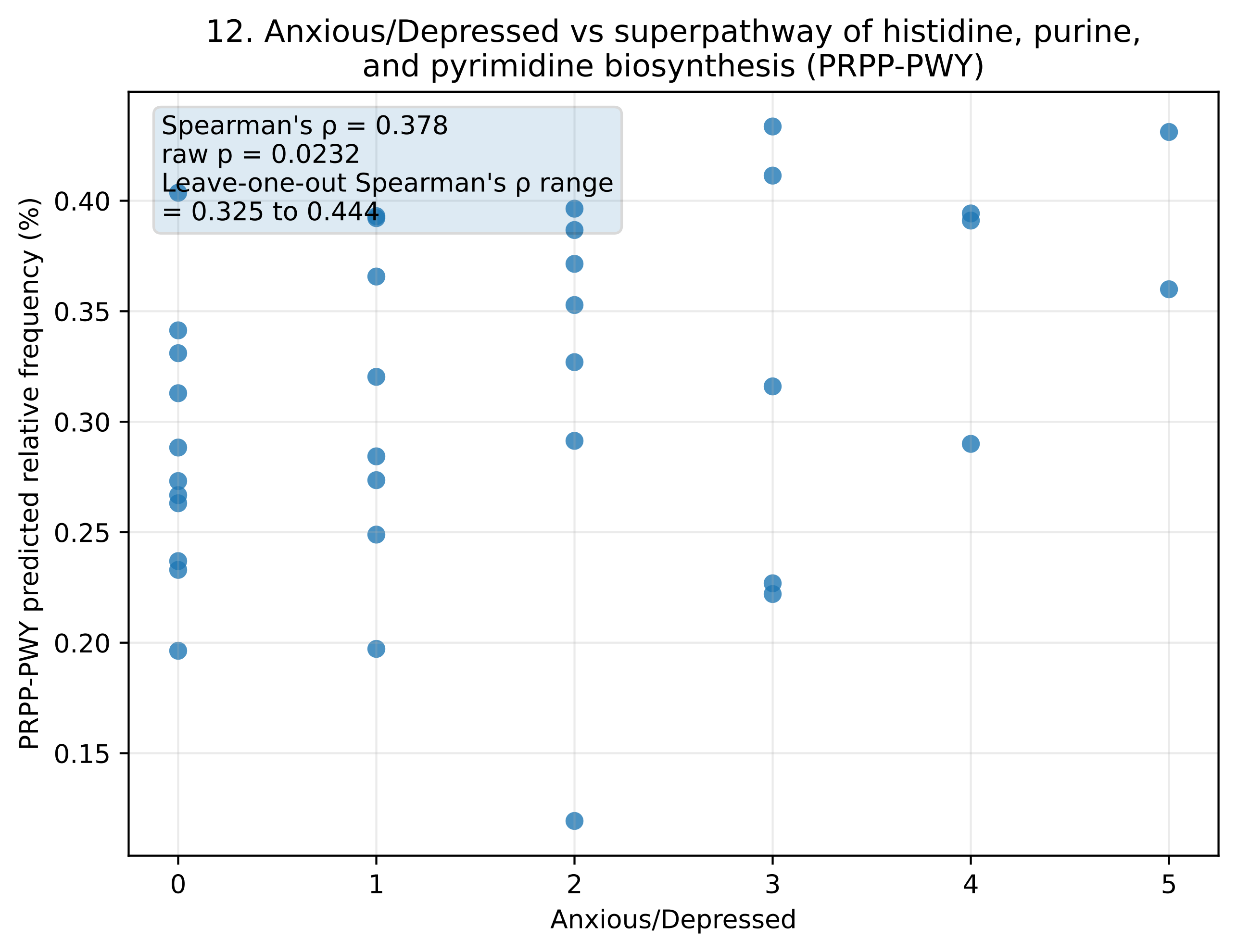
12. Anxious/Depressed vs superpathway of histidine, purine, and pyrimidine biosynthesis (PRPP-PWY)
Spearman's ρ = 0.378
raw p = 0.0232
Leave-one-out Spearman's ρ range
= 0.325 to 0.444
PRPP-PWY predicted relative frequency (%)
0.15
0.20
0.25
0.30
0.35
0.40
0
1
2
3
4
5
Anxious/Depressed

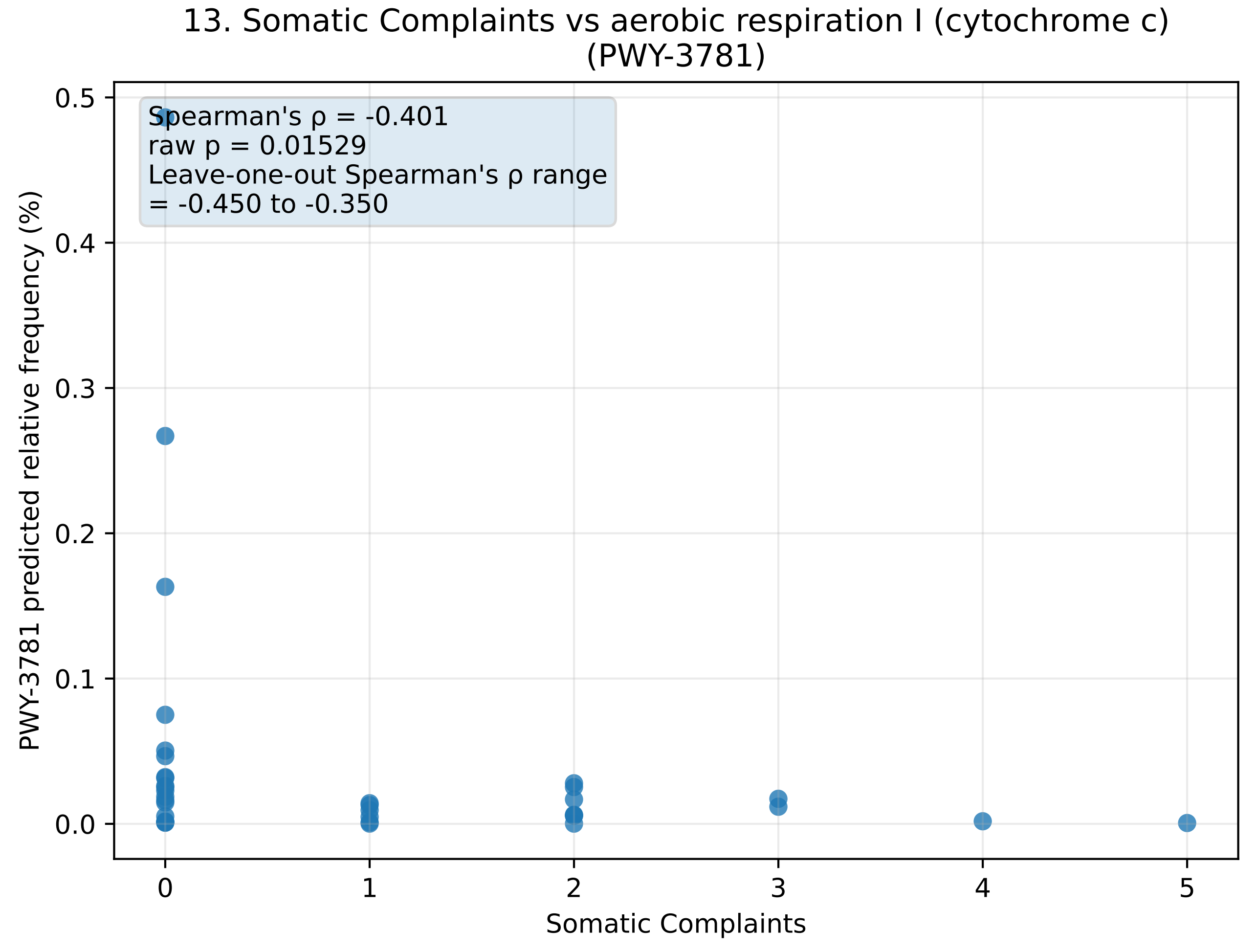
13. Somatic Complaints vs aerobic respiration I (cytochrome c)
(PWY-3781)
Spearman's ρ = -0.401
raw p = 0.01529
Leave-one-out Spearman's ρ range
= -0.450 to -0.350
PWY-3781 predicted relative frequency (%)
Somatic Complaints
0.0
0.1
0.2
0.3
0.4
0.5
0
1
2
3
4
5

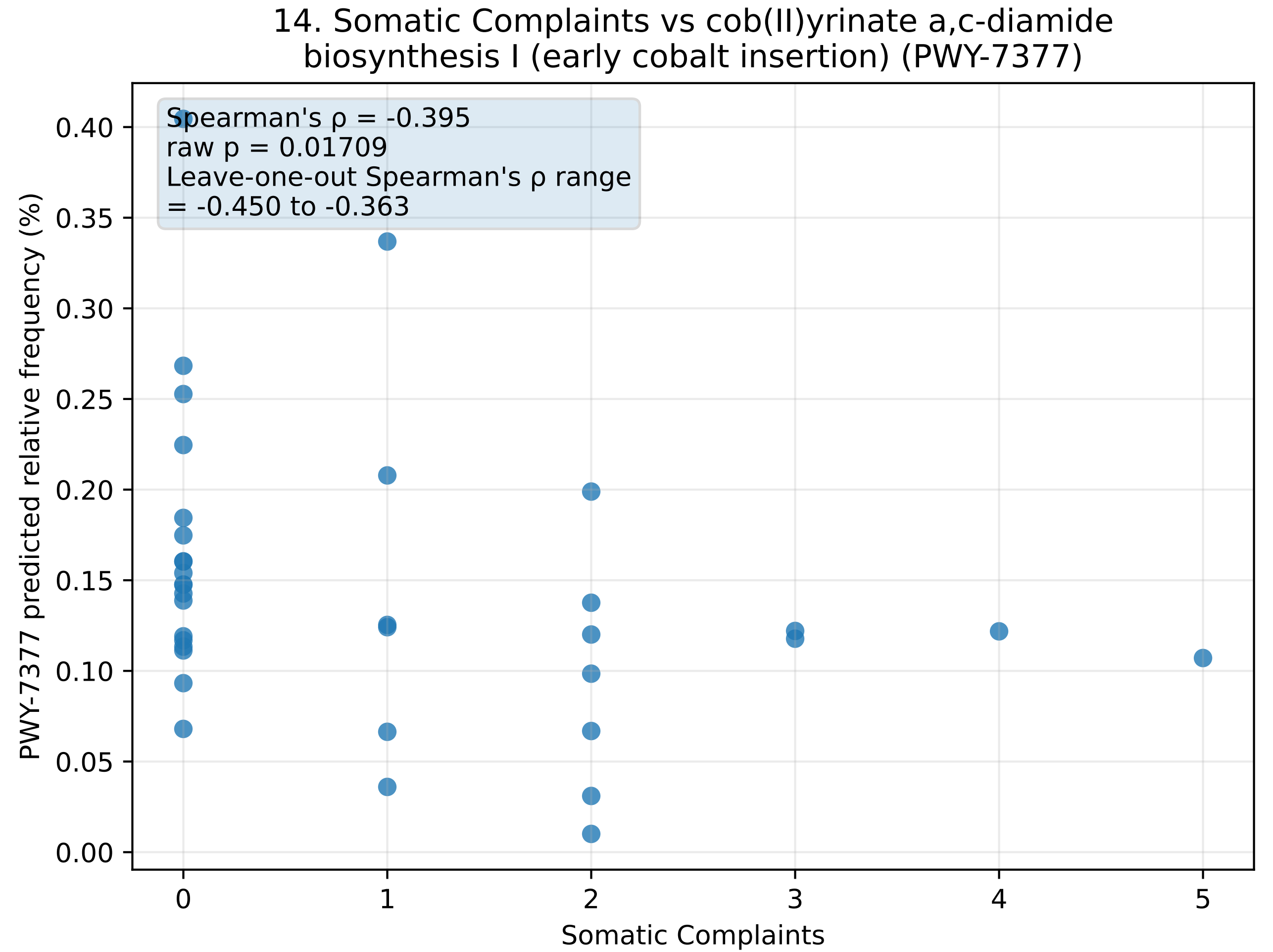
14. Somatic Complaints vs cob(II)yrinate a,c-diamide biosynthesis I (early cobalt insertion) (PWY-7377)
Spearman's ρ = -0.395
raw p = 0.01709
Leave-one-out Spearman's ρ range = -0.450 to -0.363
PWY-7377 predicted relative frequency (%)
0.00
0.05
0.10
0.15
0.20
0.25
0.30
0.35
0.40
0
1
2
3
4
5
Somatic Complaints

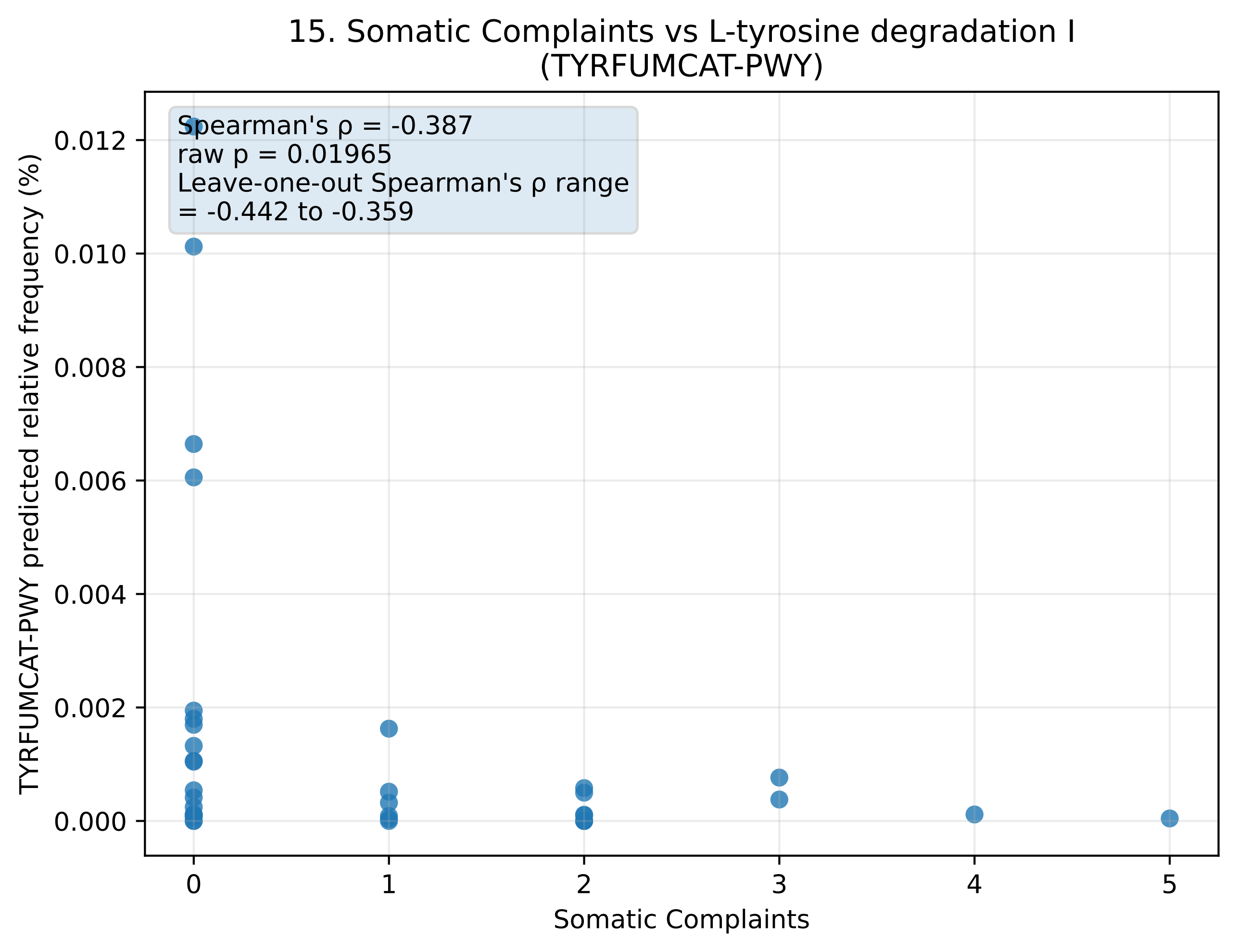
15. Somatic Complaints vs L-tyrosine degradation I
(TYRFUMCAT-PWY)
Spearman's ρ = -0.387
raw p = 0.01965
Leave-one-out Spearman's ρ range
= -0.442 to -0.359
TYRFUMCAT-PWY predicted relative frequency (%)
0.000
0.002
0.004
0.006
0.008
0.010
0.012
0
1
2
3
4
5
Somatic Complaints

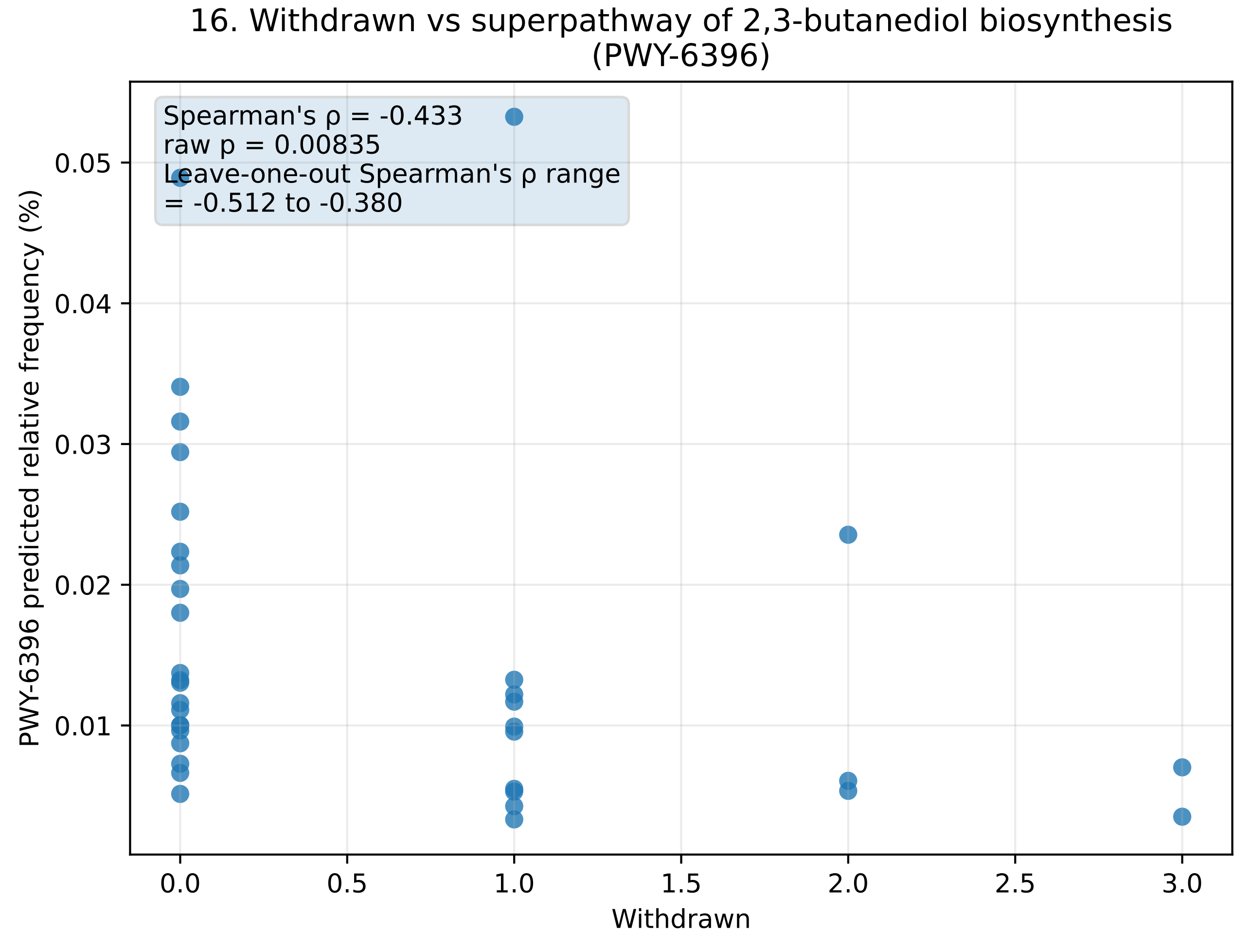
16. Withdrawn vs superpathway of 2,3-butanediol biosynthesis
(PWY-6396)
Spearman's ρ = -0.433
raw p = 0.00835
Leave-one-out Spearman's ρ range
= -0.512 to -0.380
PWY-6396 predicted relative frequency (%)
0.01
0.02
0.03
0.04
0.05
Withdrawn
0.0
0.5
1.0
1.5
2.0
2.5
3.0

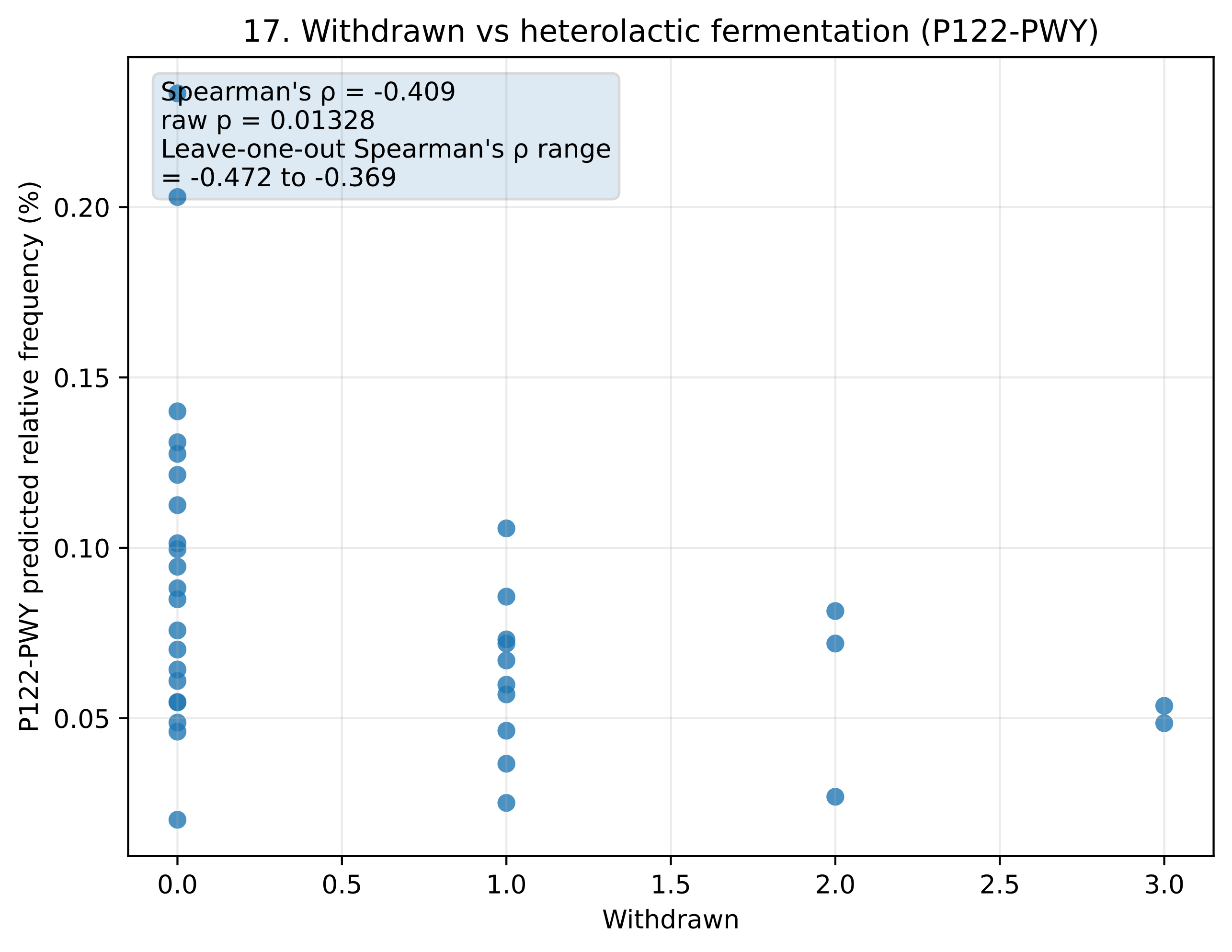
17. Withdrawn vs heterolactic fermentation (P122-PWY)
Spearman's ρ = -0.409
raw p = 0.01328
Leave-one-out Spearman's ρ range
= -0.472 to -0.369
P122-PWY predicted relative frequency (%)
0.05
0.10
0.15
0.20
0.0
0.5
1.0
1.5
2.0
2.5
3.0
Withdrawn

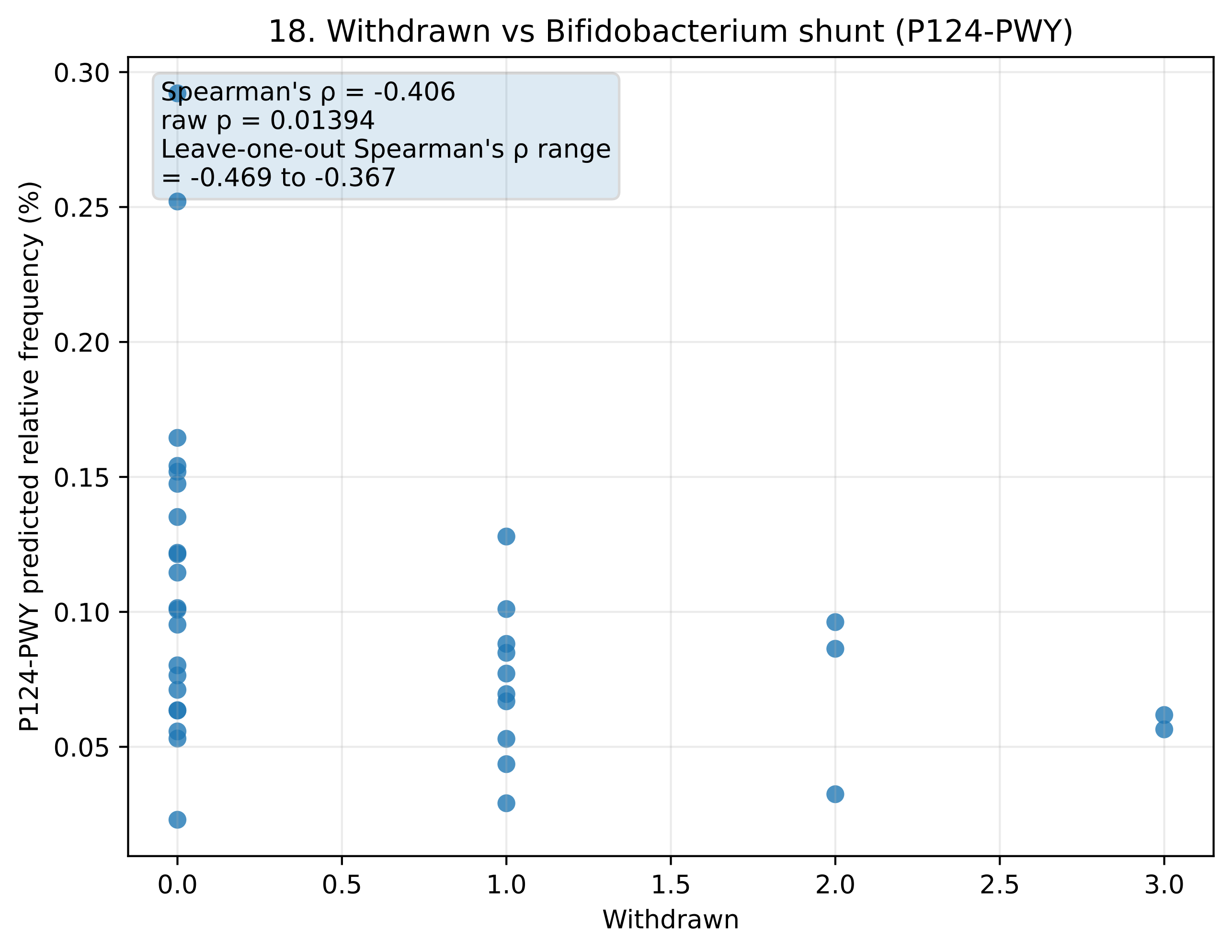
18. Withdrawn vs Bifidobacterium shunt (P124-PWY)
Spearman's ρ = -0.406
raw p = 0.01394
Leave-one-out Spearman's ρ range
= -0.469 to -0.367
P124-PWY predicted relative frequency (%)
0.30
0.25
0.20
0.15
0.10
0.05
0.0
0.5
1.0
1.5
2.0
2.5
3.0
Withdrawn

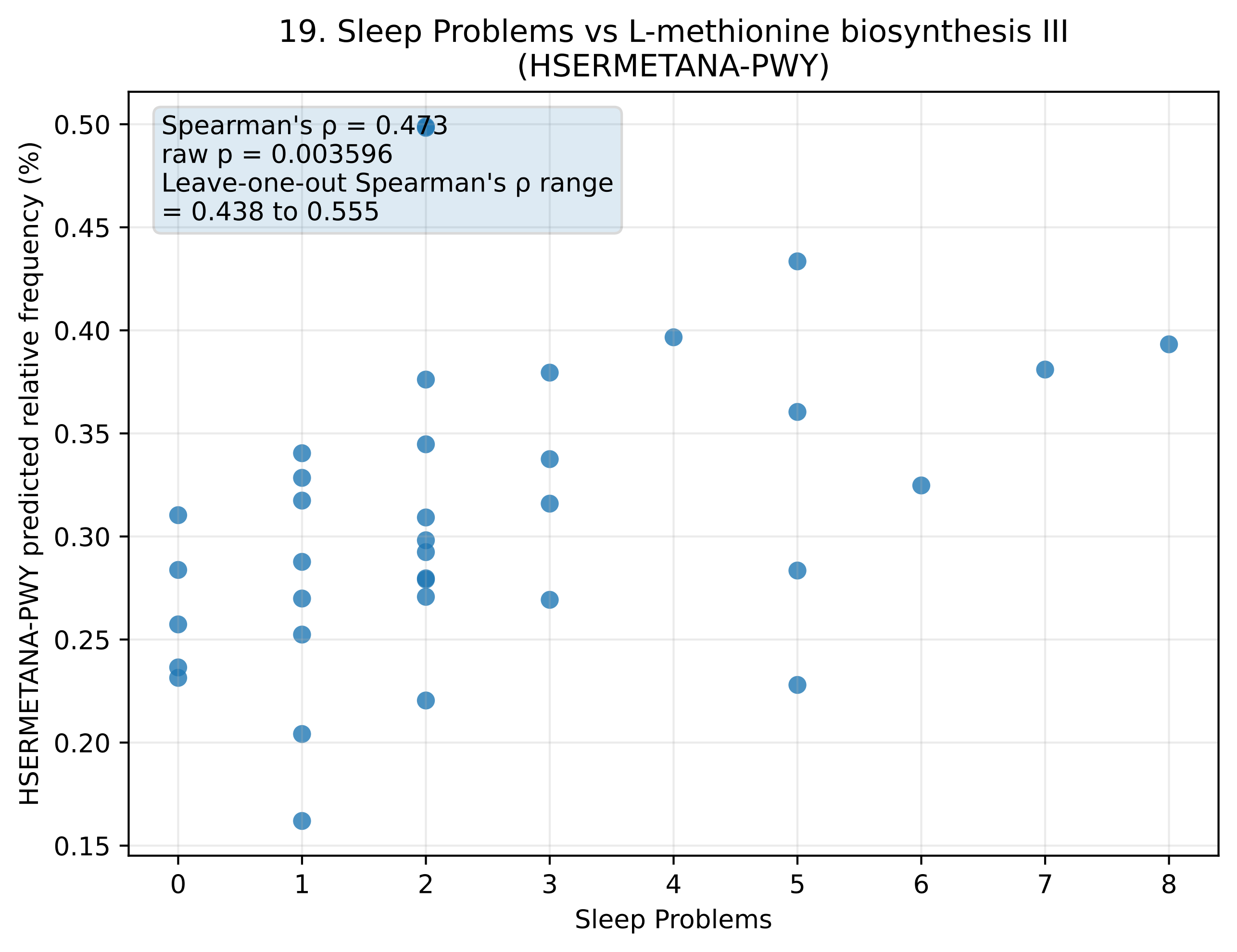
19. Sleep Problems vs L-methionine biosynthesis III
(HSERMETANA-PWY)
Spearman's ρ = 0.473
raw p = 0.003596
Leave-one-out Spearman's ρ range
= 0.438 to 0.555
HSERMETANA-PWY predicted relative frequency (%)
0.50
0.45
0.40
0.35
0.30
0.25
0.20
0.15
0
1
2
3
4
5
6
7
8
Sleep Problems

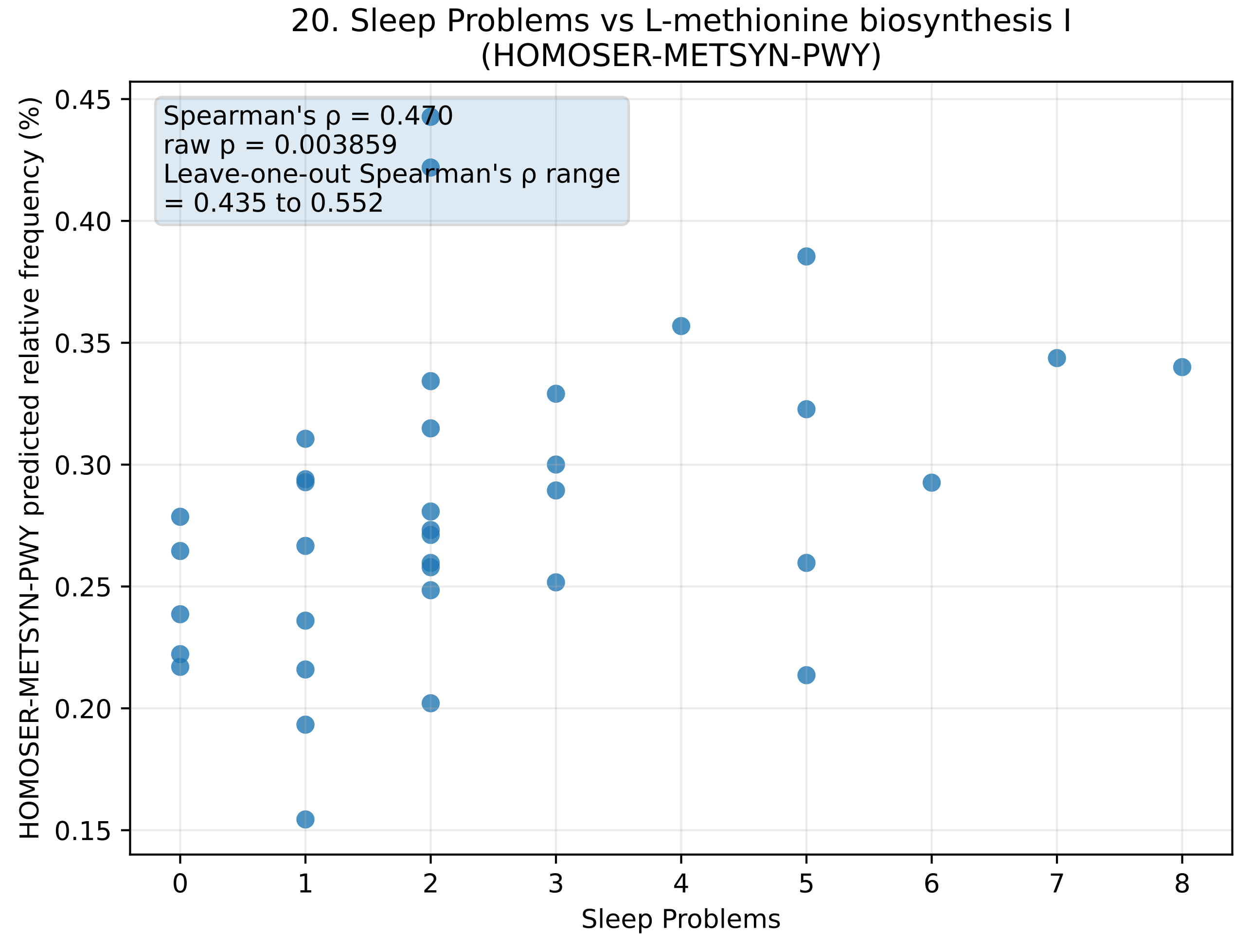
20. Sleep Problems vs L-methionine biosynthesis I
(HOMOSER-METSYN-PWY)
Spearman's ρ = 0.470
raw p = 0.003859
Leave-one-out Spearman's ρ range
= 0.435 to 0.552
HOMOSER-METSYN-PWY predicted relative frequency (%)
0.15
0.20
0.25
0.30
0.35
0.40
0.45
0
1
2
3
4
5
6
7
8
Sleep Problems

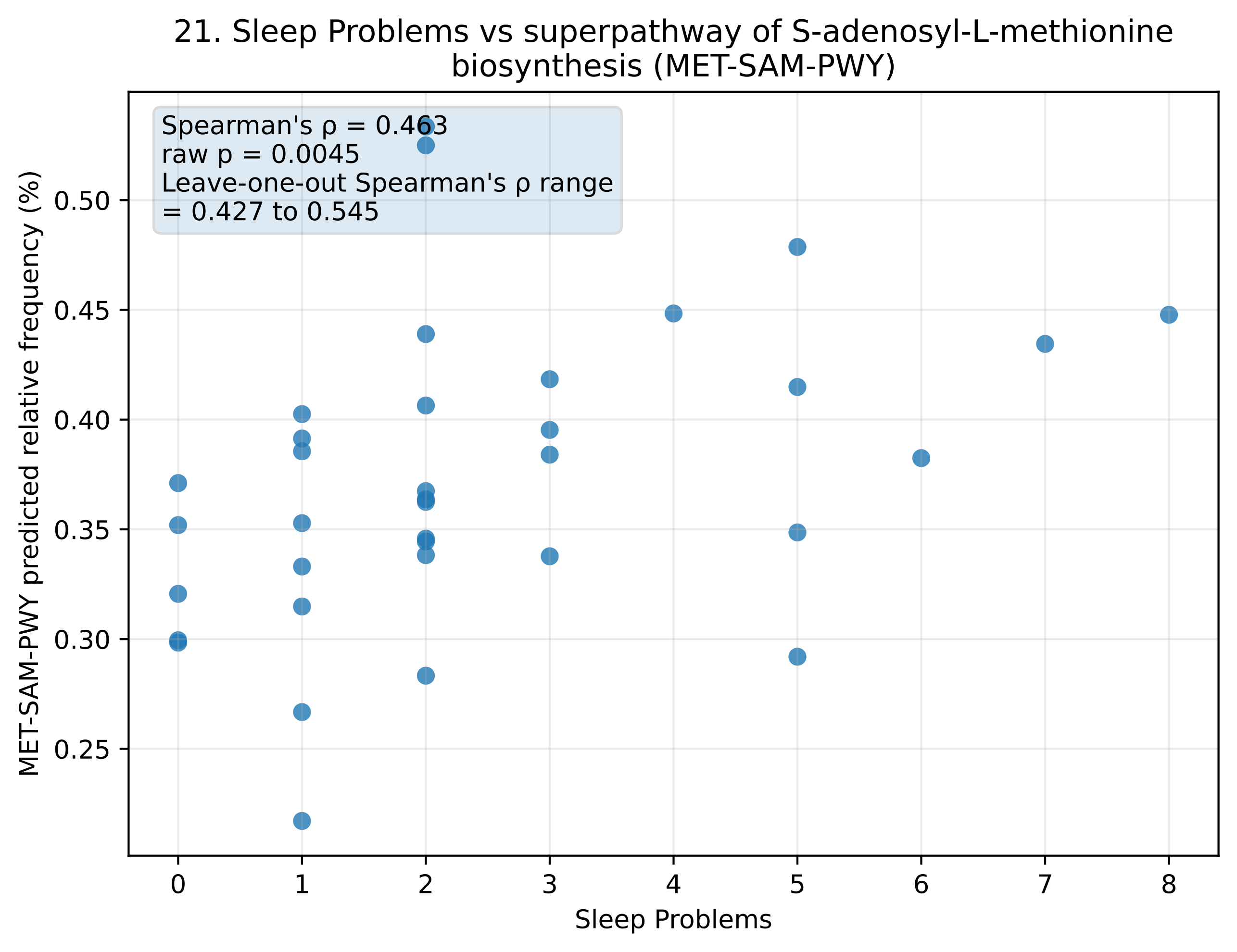
21. Sleep Problems vs superpathway of S-adenosyl-L-methionine biosynthesis (MET-SAM-PWY)
Spearman's ρ = 0.463
raw p = 0.0045
Leave-one-out Spearman's ρ range = 0.427 to 0.545
MET-SAM-PWY predicted relative frequency (%)
0.25
0.30
0.35
0.40
0.45
0.50
0
1
2
3
4
5
6
7
8
Sleep Problems

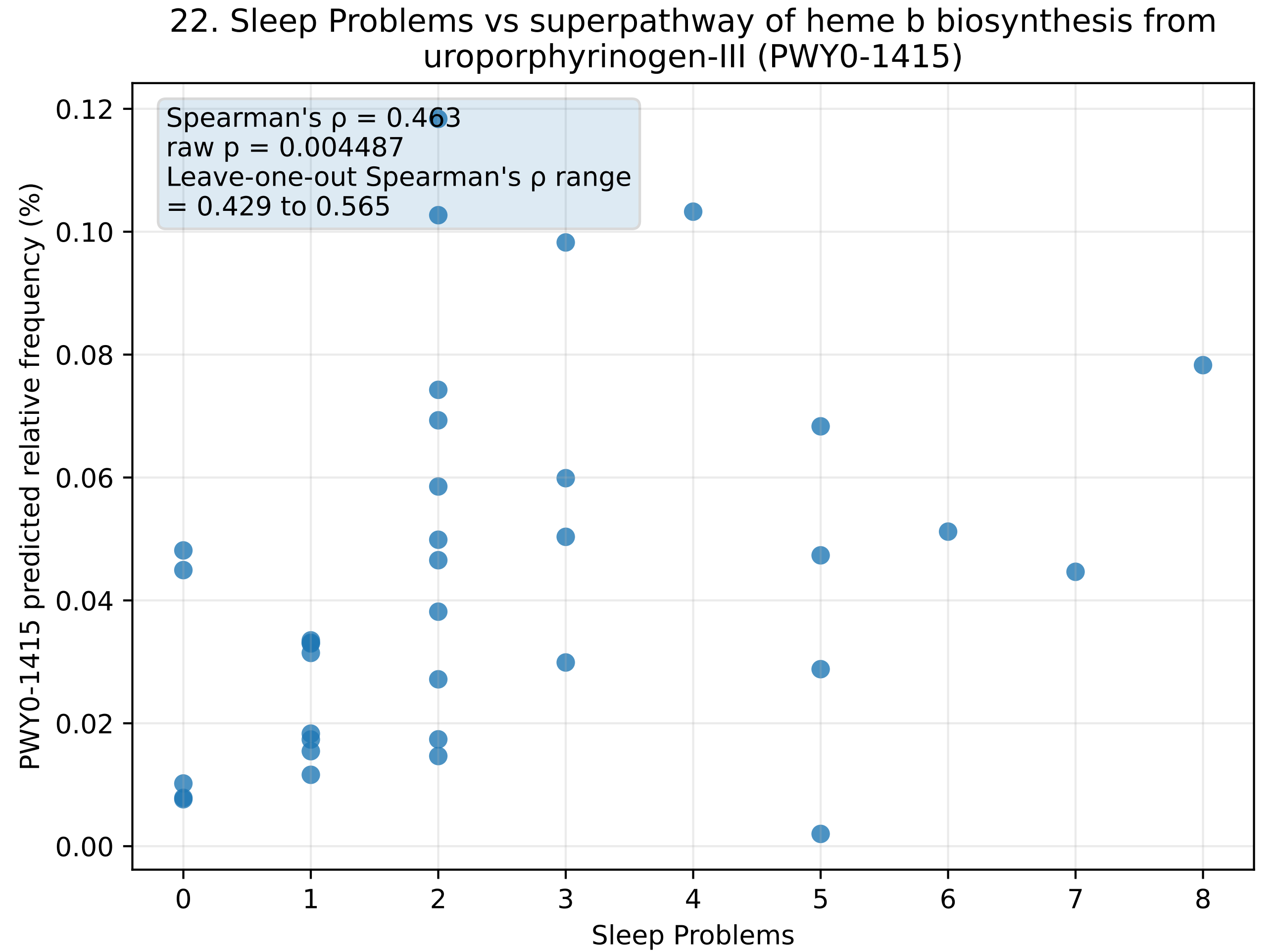
22. Sleep Problems vs superpathway of heme b biosynthesis from uroporphyrinogen-III (PWY0-1415)
Spearman's ρ = 0.463
raw p = 0.004487
Leave-one-out Spearman's ρ range
= 0.429 to 0.565
PWY0-1415 predicted relative frequency (%)
0.00
0.02
0.04
0.06
0.08
0.10
0.12
0
1
2
3
4
5
6
7
8
Sleep Problems

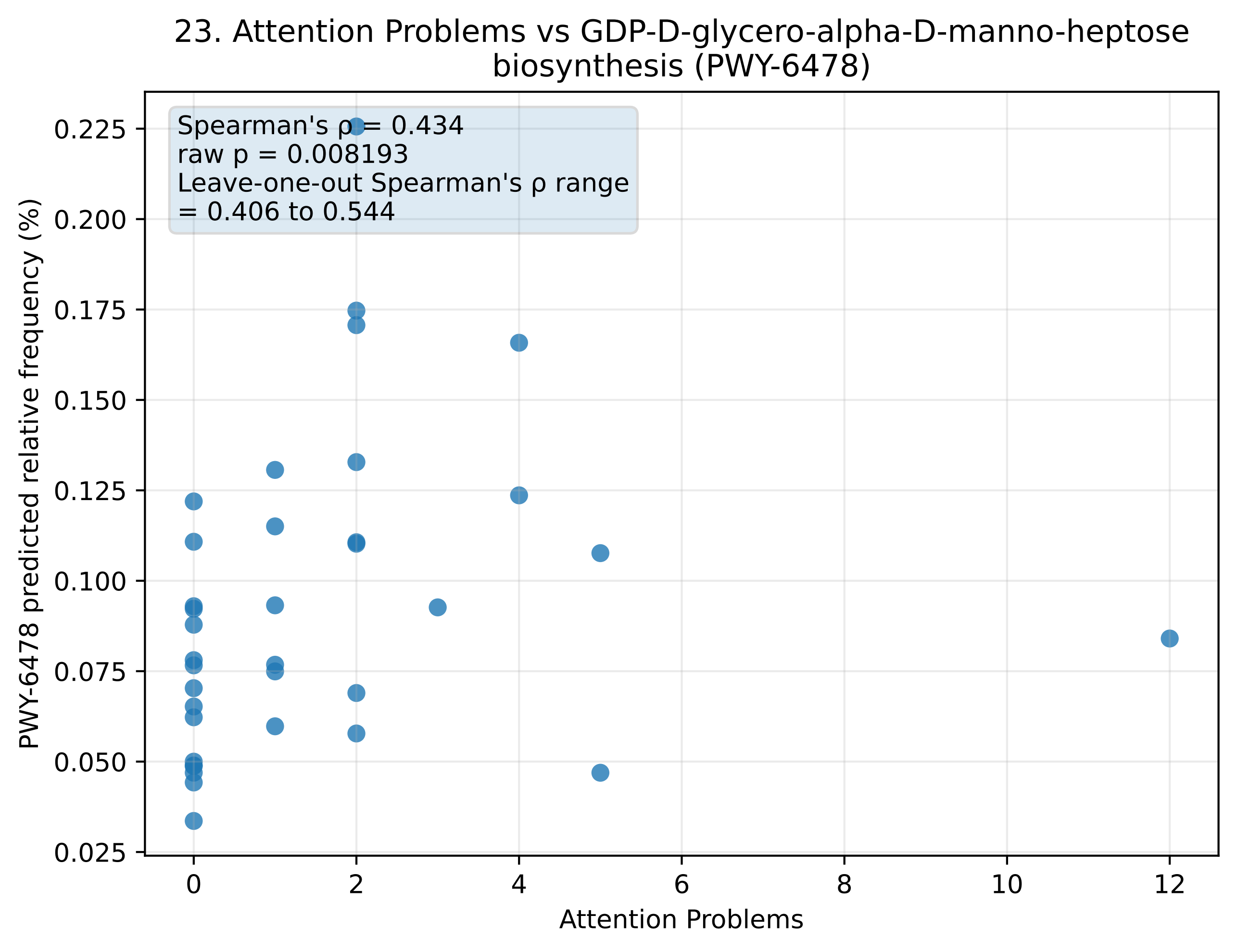

23. Attention Problems vs GDP-D-glycero-alpha-D-manno-heptose biosynthesis (PWY-6478)
Spearman's ρ = 0.434
raw p = 0.008193
Leave-one-out Spearman's ρ range = 0.406 to 0.544
PWY-6478 predicted relative frequency (%)
0.225
0.200
0.175
0.150
0.125
0.100
0.075
0.050
0.025
0
2
4
6
8
10
12
Attention Problems

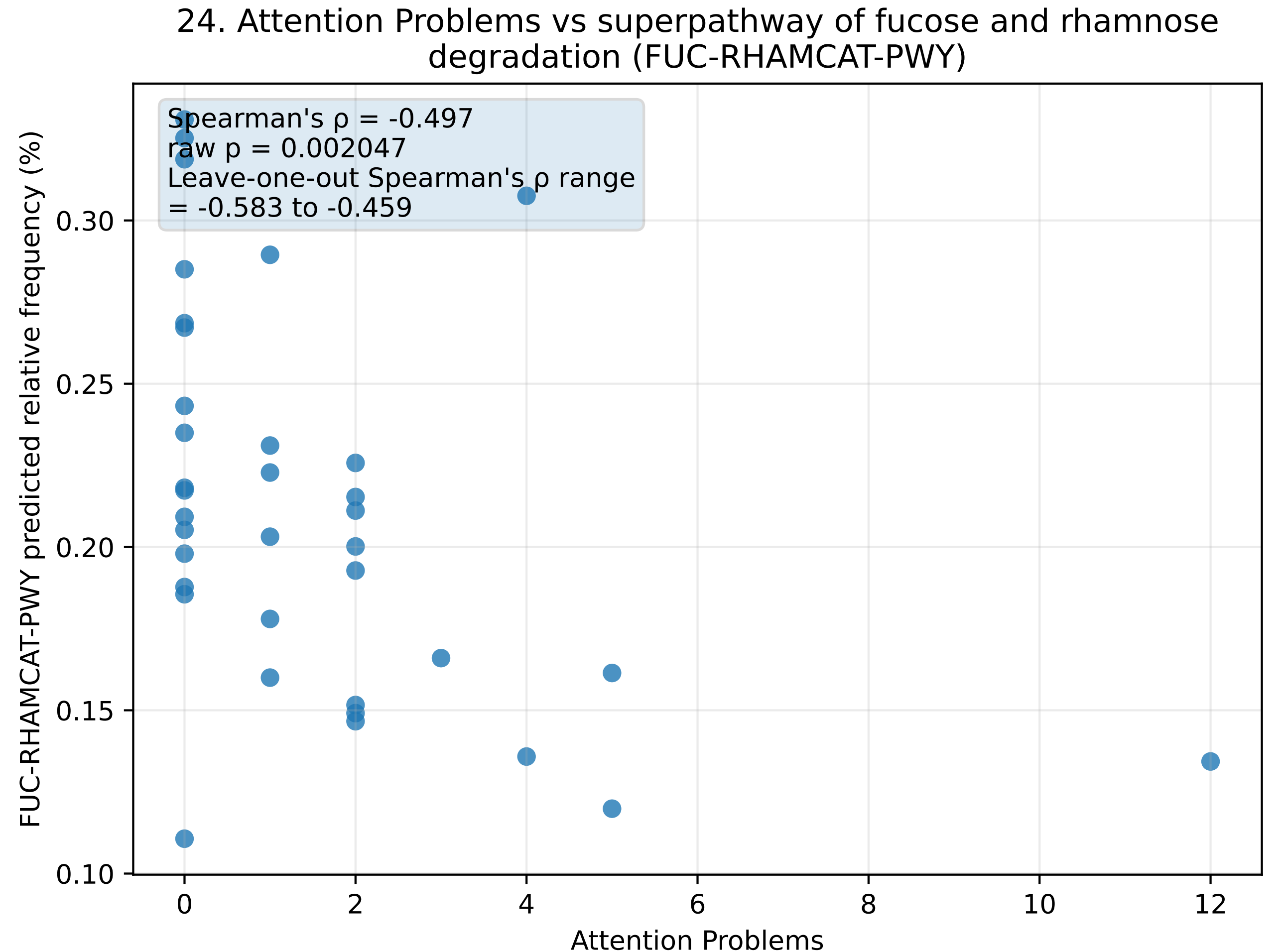
24. Attention Problems vs superpathway of fucose and rhamnose degradation (FUC-RHAMCAT-PWY)
Spearman's ρ = -0.497
raw p = 0.002047
Leave-one-out Spearman's ρ range = -0.583 to -0.459
FUC-RHAMCAT-PWY predicted relative frequency (%)
Attention Problems
0.10
0.15
0.20
0.25
0.30
0
2
4
6
8
10
12

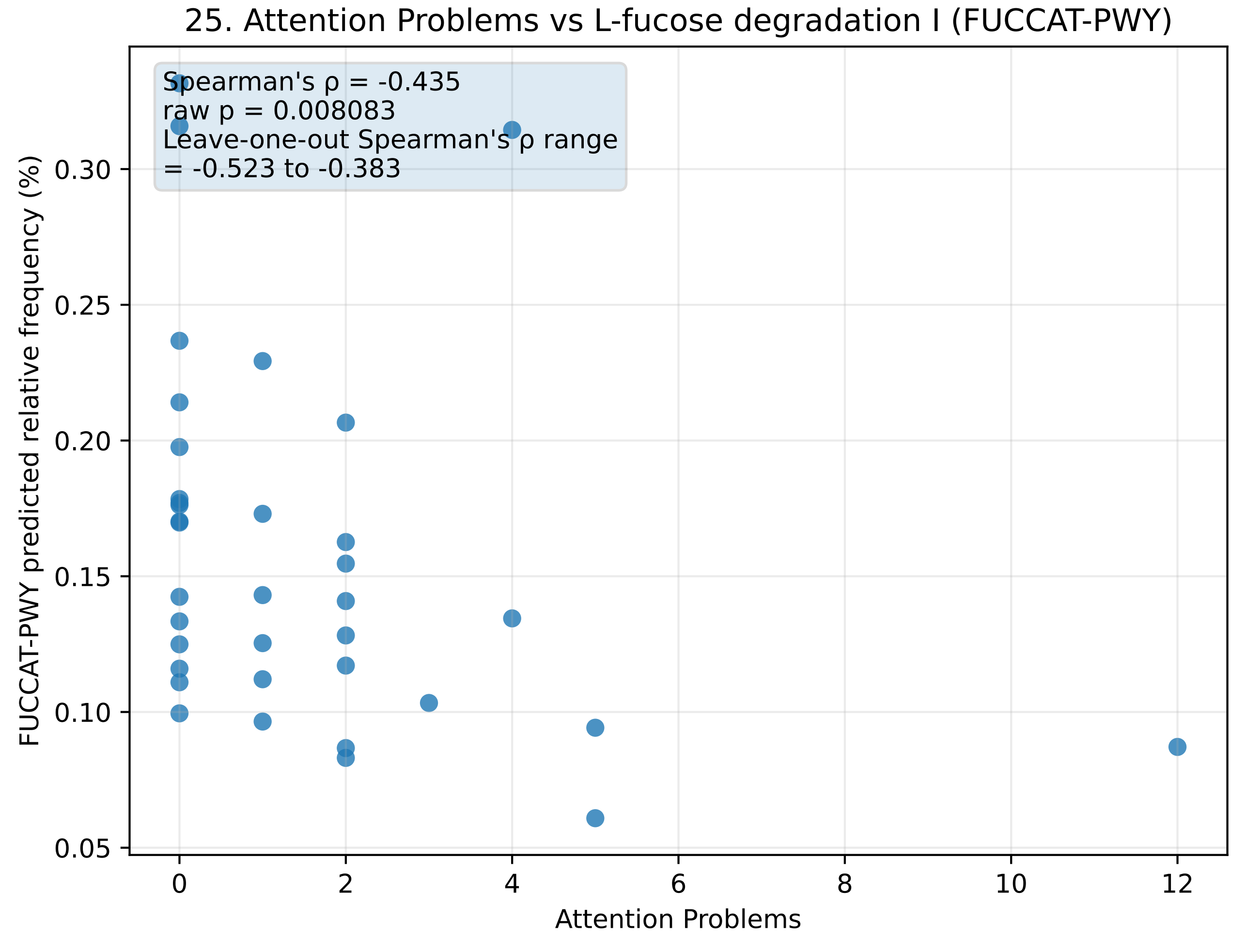
25. Attention Problems vs L-fucose degradation I (FUCCAT-PWY)
Spearman's ρ = -0.435
raw p = 0.008083
Leave-one-out Spearman's ρ range
= -0.523 to -0.383
FUCCAT-PWY predicted relative frequency (%)
0.05
0.10
0.15
0.20
0.25
0.30
0
2
4
6
8
10
12
Attention Problems

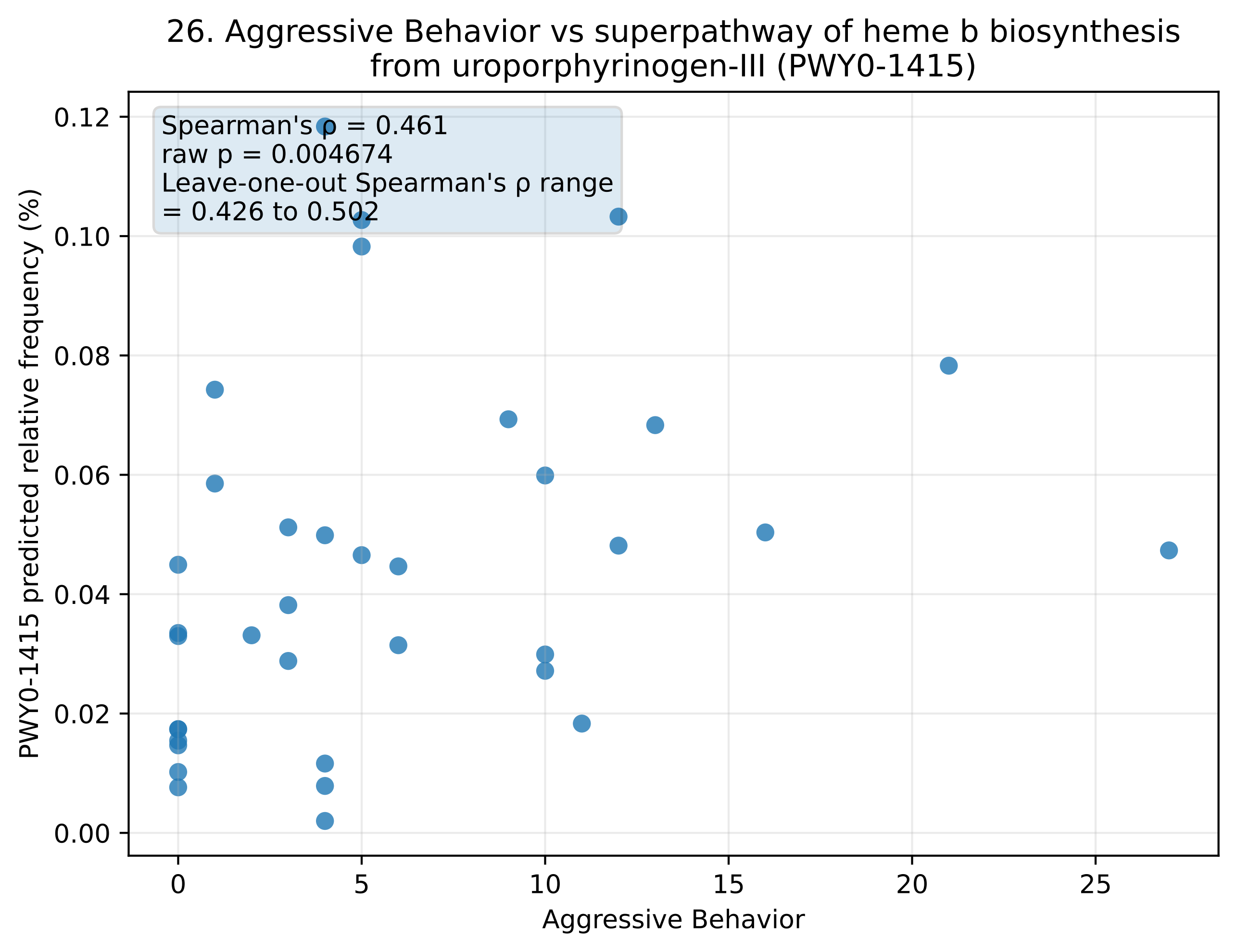
26. Aggressive Behavior vs superpathway of heme b biosynthesis from uroporphyrinogen-III (PWY0-1415)
Spearman's ρ = 0.461
raw p = 0.004674
Leave-one-out Spearman's ρ range = 0.426 to 0.502
PWY0-1415 predicted relative frequency (%)
Aggressive Behavior
0.00
0.02
0.04
0.06
0.08
0.10
0.12
0
5
10
15
20
25

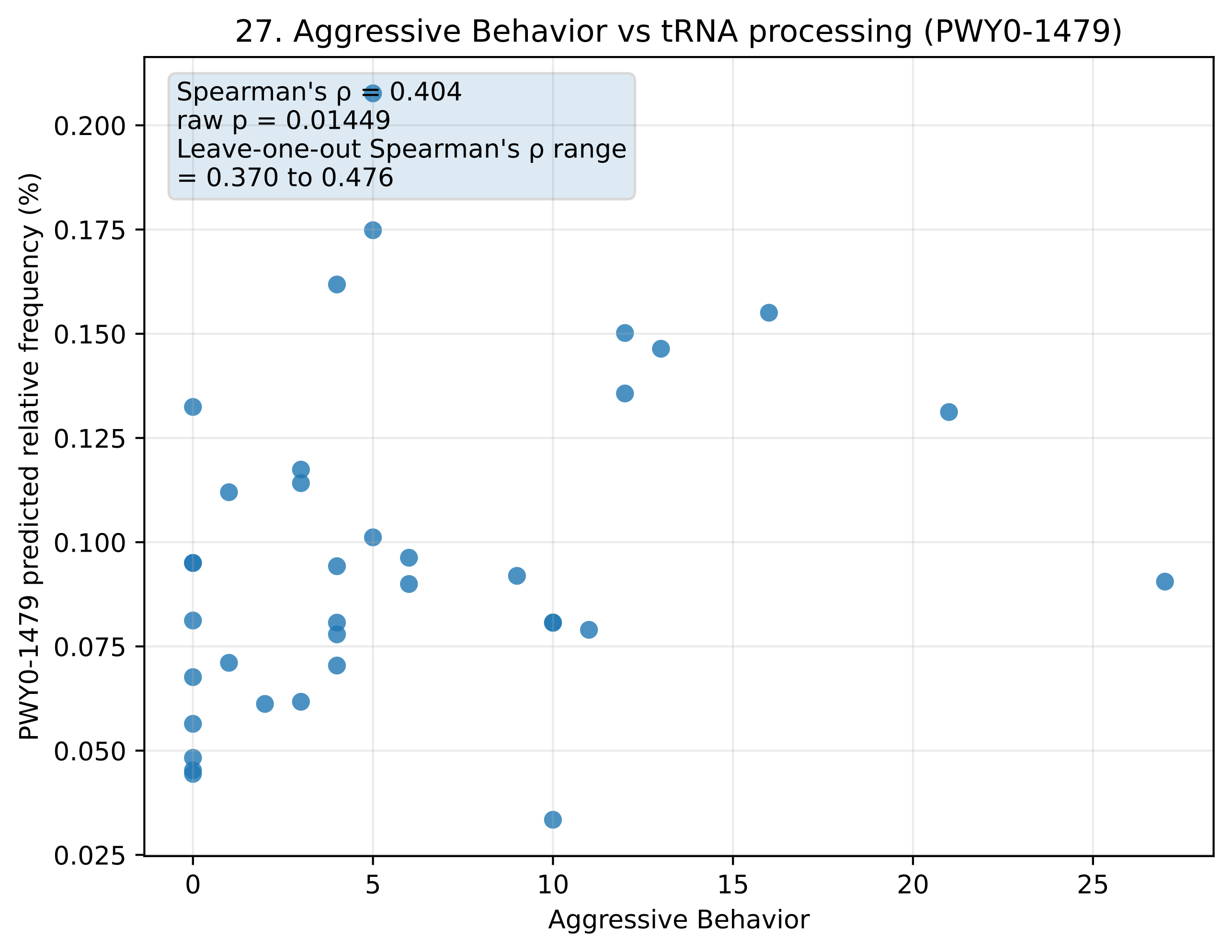
27. Aggressive Behavior vs tRNA processing (PWY0-1479)
Spearman's ρ = 0.404
raw p = 0.01449
Leave-one-out Spearman's ρ range
= 0.370 to 0.476
PWY0-1479 predicted relative frequency (%)
Aggressive Behavior
0.025
0.050
0.075
0.100
0.125
0.150
0.175
0.200
0
5
10
15
20
25

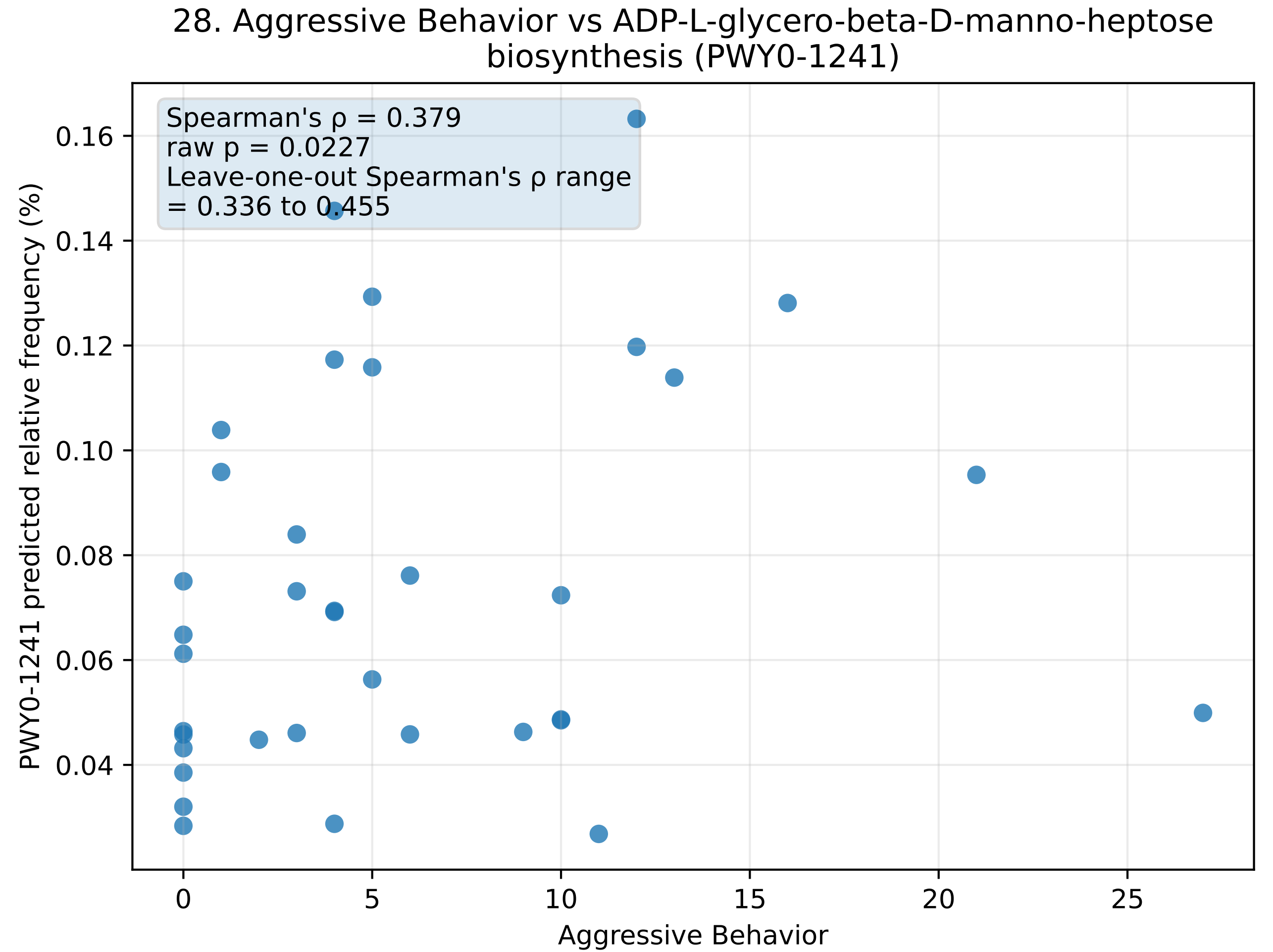
28. Aggressive Behavior vs ADP-L-glycero-beta-D-manno-heptose biosynthesis (PWY0-1241)
Spearman's ρ = 0.379
raw p = 0.0227
Leave-one-out Spearman's ρ range = 0.336 to 0.455
PWY0-1241 predicted relative frequency (%)
0.16
0.14
0.12
0.10
0.08
0.06
0.04
0
5
10
15
20
25
Aggressive Behavior
